# *PIK3CA* missense mutations promote glioblastoma pathogenesis, but do not enhance targeted PI3K inhibition

**DOI:** 10.1371/journal.pone.0200014

**Published:** 2018-07-05

**Authors:** Robert S. McNeill, Emily E. Stroobant, Erin Smithberger, Demitra A. Canoutas, Madison K. Butler, Abigail K. Shelton, Shrey D. Patel, Juanita C. Limas, Kasey R. Skinner, Ryan E. Bash, Ralf S. Schmid, C. Ryan Miller

**Affiliations:** 1 Pathobiology and Translational Science Graduate Program, University of North Carolina School of Medicine, Chapel Hill, NC, United States of America; 2 Department of Chemistry, University of North Carolina School of Medicine, Chapel Hill, NC, United States of America; 3 Department of Biology, University of North Carolina School of Medicine, Chapel Hill, NC, United States of America; 4 Department of Pharmacology, University of North Carolina School of Medicine, Chapel Hill, NC, United States of America; 5 Neurosciences Center, University of North Carolina School of Medicine, Chapel Hill, NC, United States of America; 6 Departments of Pathology and Laboratory Medicine, University of North Carolina School of Medicine, Chapel Hill, NC, United States of America; 7 Lineberger Comprehensive Cancer Center, University of North Carolina School of Medicine, Chapel Hill, NC, United States of America; 8 Department of Neurology, University of North Carolina School of Medicine, Chapel Hill, NC, United States of America; University of Alabama at Birmingham, UNITED STATES

## Abstract

**Background:**

Glioblastoma (GBM) is the most common adult primary brain tumor. Multimodal treatment is empiric and prognosis remains poor. Recurrent *PIK3CA* missense mutations (*PIK3CA*^*mut*^) in GBM are restricted to three functional domains: adaptor binding (ABD), helical, and kinase. Defining how these mutations influence gliomagenesis and response to kinase inhibitors may aid in the clinical development of novel targeted therapies in biomarker-stratified patients.

**Methods:**

We used normal human astrocytes immortalized via expression of hTERT, E6, and E7 (NHA). We selected two *PIK3CA*^*mut*^ from each of 3 mutated domains and induced their expression in NHA with (NHA^RAS^) and without mutant *RAS* using lentiviral vectors. We then examined the role of *PIK3CA*^*mut*^ in gliomagenesis *in vitro* and in mice, as well as response to targeted PI3K (PI3Ki) and MEK (MEKi) inhibitors *in vitro*.

**Results:**

*PIK3CA*^*mut*^, particularly helical and kinase domain mutations, potentiated proximal PI3K signaling and migration of NHA and NHA^RAS^ *in vitro*. Only kinase domain mutations promoted NHA colony formation, but both helical and kinase domain mutations promoted NHA^RAS^ tumorigenesis *in vivo*. *PIK3CA*^*mut*^ status had minimal effects on PI3Ki and MEKi efficacy. However, PI3Ki/MEKi synergism was pronounced in NHA and NHA^RAS^ harboring ABD or helical mutations.

**Conclusion:**

*PIK3CA*^*mut*^ promoted differential gliomagenesis based on the mutated domain. While *PIK3CA*^*mut*^ did not influence sensitivity to single agent PI3Ki, they did alter PI3Ki/MEKi synergism. Taken together, our results demonstrate that a subset of *PIK3CA*^*mut*^ promote tumorigenesis and suggest that patients with helical domain mutations may be most sensitive to dual PI3Ki/MEKi treatment.

## Introduction

Glioblastoma (GBM) is the most common primary malignant brain tumor in adults [1]. It is also the most aggressive, with a median survival of only 12–15 months [1–3]. The molecular heterogeneity of GBM has been extensively characterized [4–6]. The vast majority of GBM arise *de novo* and harbor frequent mutations in 3 “core” signaling pathways: RB, TP53, and receptor tyrosine kinase (RTK)/mitogen activated protein kinase (MAPK)/phosphoinositide 3-kinase (PI3K) [5]. GBM can be stratified into 4 molecular subtypes based on gene expression [6]. However, this knowledge has yet to impact patient management. First line therapy remains empiric and consists of surgical resection followed by radiation with concurrent and adjuvant temozolomide, a DNA damaging agent [3]. Clinical trials of inhibitors targeting the pathways frequently mutated in GBM have had disappointing results for a variety of reasons, including drug resistance and inclusion of molecularly heterogeneous patients [7, 8]. Preclinical modeling can aid in development of novel therapies by defining whether mutations associated with GBM drive disease pathogenesis and are predictive of drug response.

The PI3K pathway promotes many cancer hallmarks, including survival, proliferation, and migration/invasion [9–12]. PI3K is a heterodimeric lipid kinase composed of catalytic and regulatory subunits encoded by genes such as *PIK3CA* and *PIK3R1*, respectively [13, 14]. Pathway activation is mediated by the phosphorylation of PIP_2_ to PIP_3_ by the catalytic subunit, resulting in recruitment and activation of effector proteins, including AKT. PI3K signaling (hereafter PI3K) is antagonized by the tumor suppressor *PTEN* [13, 14]. The PI3K pathway is an attractive therapeutic target in GBM because mutually exclusive mutations in *PIK3CA*, *PIK3R1*, and *PTEN* occur in 46% of patients [15–17].

PI3K activation via *Pten* deletion, *PIK3R1* mutation, or constitutive *AKT* promotes tumorigenesis in multiple preclinical GBM models [18–23]. For example, we found that *Pten* deletion cooperates with mutant *Kras* to activate PI3K and potentiate malignant progression in immortalized mouse astrocytes [19, 24]. Similarly, constitutive *AKT* cooperated with mutant *RAS* to promote tumorigenesis in immortalized human astrocytes (NHA) [22, 25]. However, the role of *PIK3CA* mutations in gliomagenesis has not been experimentally investigated.

*PIK3CA* is altered in 10% of GBM, mostly via missense mutations [4, 15, 16]. These mutations are generally restricted to 3 functional protein domains (adaptor binding (ABD), helical, and kinase) and are predicted to activate PI3K via distinct biochemical mechanisms [26]. Some *PIK3CA*^*mut*^ found in GBM have been shown to promote tumorigenesis in non-brain tissues, particularly the most prevalent helical (E542K, E545K) and kinase (H1047R) domain mutations [27–29]. However, their role in gliomagenesis has yet to be determined. Here we defined the role of *PIK3CA*^*mut*^ in GBM pathogenesis using NHA and NHA^RAS^. Furthermore, we determined whether these mutations influenced response to single agent and combination PI3K/MEK inhibitors buparlisib and selumetinib, respectively, to elucidate the utility of *PIK3CA*^*mut*^ as a predictive biomarker.

Buparlisib (BKM120) has been proposed as a potential targeted therapy for GBM [30–33]. It is currently being investigated in a Phase II clinical trial in GBM patients (ClinicalTrials.gov, NCT01339052). While there have been no GBM clinical trials of selumetinib, we and others have shown its efficacy in preclinical models [34, 35]. Enriching future clinical trials with likely responders based on mutational profiles promises to improve the chances of clinical success.

## Materials and methods

### Supplement

Supplemental methods, figures, and tables can be found online.

### *PIK3CA* mutagenesis and lentivirus production

Third generation lentiviral gateway destination vector (pLenti-PGK-Hygro-DEST, #19066, a gift from Eric Campeau and Paul Kaufman), pENTR4 vector (pENTR4-no-ccDB, #17424, a gift from Eric Campeau and Paul Kaufman), hemagglutinin (HA)-tagged wild-type (WT) *PIK3CA* (*PIK3CA*^*WT*^, pBabe-puro-HAPIK3CA, #12522, a gift from Jean Zhao), and HA-tagged *GFP* (GFP, pDEST-Flag-HA-GFP, #22612, a gift from Wade Harper) plasmids were purchased from Addgene (Cambridge, MA) [36–38]. Wild-type PIK3CA (*PIK3CA*^*WT*^*)* and GFP were excised and inserted into pENTR4 vector by ligation. *PIK3CA*^*mut*^ (R88Q, C90Y, E542K, E545K, M1043V, H1047R) were generated by point mutagenesis of *PIK3CA*^*WT*^ using the Q5 Site-Directed Mutagenesis Kit (New England Biolabs, Ipswich, MA) per manufacturer’s instructions. *GFP*, *PIK3CA*^*WT*^, and *PIK3CA*^*mut*^ were transferred from pENTR4 to pLenti-PGK-Hygro-DEST vectors by recombination as described [36]. All mutations were confirmed by Sanger sequencing (Genewiz, South Plainfield, NJ). Lentiviral particles encoding *GFP*, *PIK3CA*^*WT*^, or individual *PIK3CA*^*mu*t^ were generated in 293FT cells (Invitrogen, Grand Island, NY) per manufacturer’s instructions.

### Cell culture

NHA and NHA^RAS^ lines were a kind gift from Russell O. Pieper [25]. Cells were maintained as adherent cultures at 37°C and 5% CO_2_ in DMEM supplemented with 5% FBS and 1% penicillin/streptomycin (complete DMEM). To generate NHA and NHA^RAS^ lines expressing GFP, *PIK3CA*^*WT*^, or *PIK3CA*^*mut*^, 135,000 and 120,000 cells respectively were plated on 60 cm^2^ plates. Lentiviruses were added two days after plating, then incubated with cells overnight in complete DMEM containing 8 μg/ml polybrene (Sigma-Aldrich, St. Louis, MO) at 37°C and 5% CO_2_. Two days post-infection, transduced cells were selected by culture in complete DMEM plus 300 μg/ml hygromycin B (Gold Biotechnology, St. Louis, MO) for 14 days. Stable gene expression was confirmed by immunoblot for the HA tag on *PIK3CA*^*wt*^ and *PIK3CA*^*mut*^. All i*n vitro* experiments were performed in DMEM with 2.5% FBS and 1% penicillin/streptomycin (low serum medium) unless otherwise stated.

### Drugs

The PI3K inhibitor (PI3Ki) buparlisib (BKM120) and the MEK inhibitor (MEKi) selumetinib (AZD6244) were purchased from MedChem Express (Monmouth Junction, NJ) or Chemietek (Indianapolis, IN) and dissolved in dimethyl sulfoxide (DMSO). In single-dose pharmacokinetics studies in human patients, maximum observed plasma concentrations for buparlisib and selumetinib were 2–5 μM (1–2 μg/mL) and 1.1–2.0 μM (0.5–0.9 μg/mL), respectively [39, 40]. Depending on experimental requirements, drugs were used at or above these clinically relevant dose ranges.

### Immunoblots

Control (parental, GFP, and *PIK3CA*^*WT*^) and *PIK3CA*^*mut*^ NHA and NHA^RAS^ cells were treated with either vehicle control (DMSO), buparlisib and/or selumetinib, or serum starved for 24 h. Proteins were isolated and immunoblots were performed as described [19, 24, 35]. Raw immunoblot images are shown in Supplemental Immunoblots. Each blot included both a molecular weight ladder and a reference standard composed of lysates of cultured TRP astrocytes. Quantification was performed using the following formula, where *x* is an individual blot and *i* is the target in question: ${relative\;intensity}_{x,i}=\frac{\frac{{target}_{i}}{{loading\;control}_{x}}}{\frac{{TRP}_{x}}{{loading\;control}_{x}}}$ [19, 24, 35]. N = 1–3 blots, mean is denoted in the corresponding figure legend. Bands annotated in red are omitted from final figures.

### Cell growth

NHA and NHA^RAS^ were plated in triplicate or quadruplicate in 96-well tissue culture plates and absorbance (cell growth) was assessed using CellTiter 96 Aqueous One Cell Proliferation Assay (MTS, Promega, Madison, WI) according to manufacturer’s instructions. Relative absorbance was measured daily as described and fit to an exponential growth equation to calculate rate constants (k) and doubling times [ln(2)/k]. Differences in growth rate constants (k) were compared using the extra-sum-of-squares F test [35].

### Cell migration

Migration rate across a cell-free gap was determined using culture inserts according to manufacturer’s instructions (Ibidi, Munich, Germany). Briefly, cells were imaged every 2 hours for 12 hours after creation of a cell free gap using a VistaVision inverted microscope equipped with a 4X objective and a DV-300 camera (VWR, Radnor, PA). Gap closure rates were calculated from 2–12 hours using linear regression and compared via ANCOVA.

### Colony formation in soft agar

Colony formation was determined as described with minor modifications [25, 41, 42]. Briefly, cells were suspended in a mixture of DMEM/0.35% agarose (Denville Scientific INC., Holliston, MA) supplemented with 2.5% FBS and 14,000 cells were plated per well in 6-well plates. Cells were maintained for 4 weeks, fixed, and stained with 0.005% crystal violet in 70% ethanol. Plates were imaged on a Typhoon Trio (GE Healthcare) and colonies ≥ 30 μm^2^ were counted.

### Mouse use

This study was carried out in strict accordance with the recommendation of the Guide for the Care and Use of Laboratory Animals of the National Institute of Health. The Institutional Animal Care and Use Committee of the University of North Carolina (Chapel Hill, NC) approved this study (Protocol #16–112). Animals were housed in a SPF facility in IVC cages with enrichment of nestlets and a shelter on corn cob bedding at a density of 2–5 animals per cage. Animals were kept on a 12-hour light/dark cycle at a temperature of 21^o^ +/- 2^o^ Celsius and were monitored daily by experienced laboratory staff following experimental initiation.

### Orthotopic xenografts

Control and *PIK3CA*^*mut*^ NHA^RAS^ lines were harvested by trypsinization, counted, and suspended in serum-free DMEM with 5% methyl cellulose. Male and female adult athymic (*Foxn1*^nu/nu^) nude mice (Charles River, Wilmington, MA; mean age ~3 months; N = 5–10 per group, mean = 9) were anesthetized with Avertin (250 mg/kg) and 2 x 10^5^ GFP, *PIK3CA*^*WT*^, *PIK3CA*^*R88Q*^, *PIK3CA*^*E542K*^, or *PIK3CA*^*H1047R*^ NHA^RAS^ cells were injected into the right basal ganglia of mice (N = 5–10 per group, mean = 9) using the coordinates 1, -2, and -4 mm (A, L, D) from bregma as previously described [19, 24]. Subjects received bupivacaine for local nerve block and a single dose of ketorolac for post-surgical analgesia. Animals were monitored daily for the onset of neurological symptoms (lethargy, hunching, seizures, paralysis, loss of righting reflex) and euthanized via CO_2_ asphyxiation within 24 hours of onset, immediately followed by brain tissue harvest. Symptoms were frequently severe at first observation due to rapid tumor progression. Animals did not die without euthanasia. Survival was determined by Kaplan-Meier analyses and was compared by log-rank tests.

### Drug response

Dose response assays using MTS were performed and IC_50_ calculated as described [24, 35]. Synergy between MEKi and PI3Ki was determined by BLISS using Combenefit v1.31 [43].

### Statistics

GraphPad Prism (La Jolla, CA) was used for statistical analyses. Error bars are SEM unless otherwise stated. P≤0.05 were considered significant.

## Results

*PIK3CA*^*mut*^ are frequent in GBM and are heterogeneously distributed across multiple encoded protein domains, including ABD, helical, and kinase (**S1A Fig**) [15, 16]. *PTEN* deletion or activating AKT mutations cooperate with activated MAPK signaling (hereafter MAPK) to promote tumorigenesis in preclinical glioma models [19, 22]. However, the role of *PIK3CA*^*mut*^ in has not been examined in these models. To this end, we examined the 2 most frequent (hotspot) helical (E542K, E545K) and kinase (M1043V, H1047R) domain *PIK3CA*^*mut*^ found in GBM and other cancer types (**S1A–S1C Fig**). ABD mutations are less prevalent in most cancers. Of these, R88Q is the most common and only recurrent mutation in GBM [4, 15]. We therefore evaluated it, as well as a second, C90Y. We transduced each of 6 *PIK3CA*^*mut*^ into NHA and NHA^RAS^ via lentiviral vectors. Parental, GFP, or *PIK3CA*^*WT*^-transduced lines served as controls.

### *PIK3CA*^mut^ induce PI3K *in vitro*

**Fig 1:**
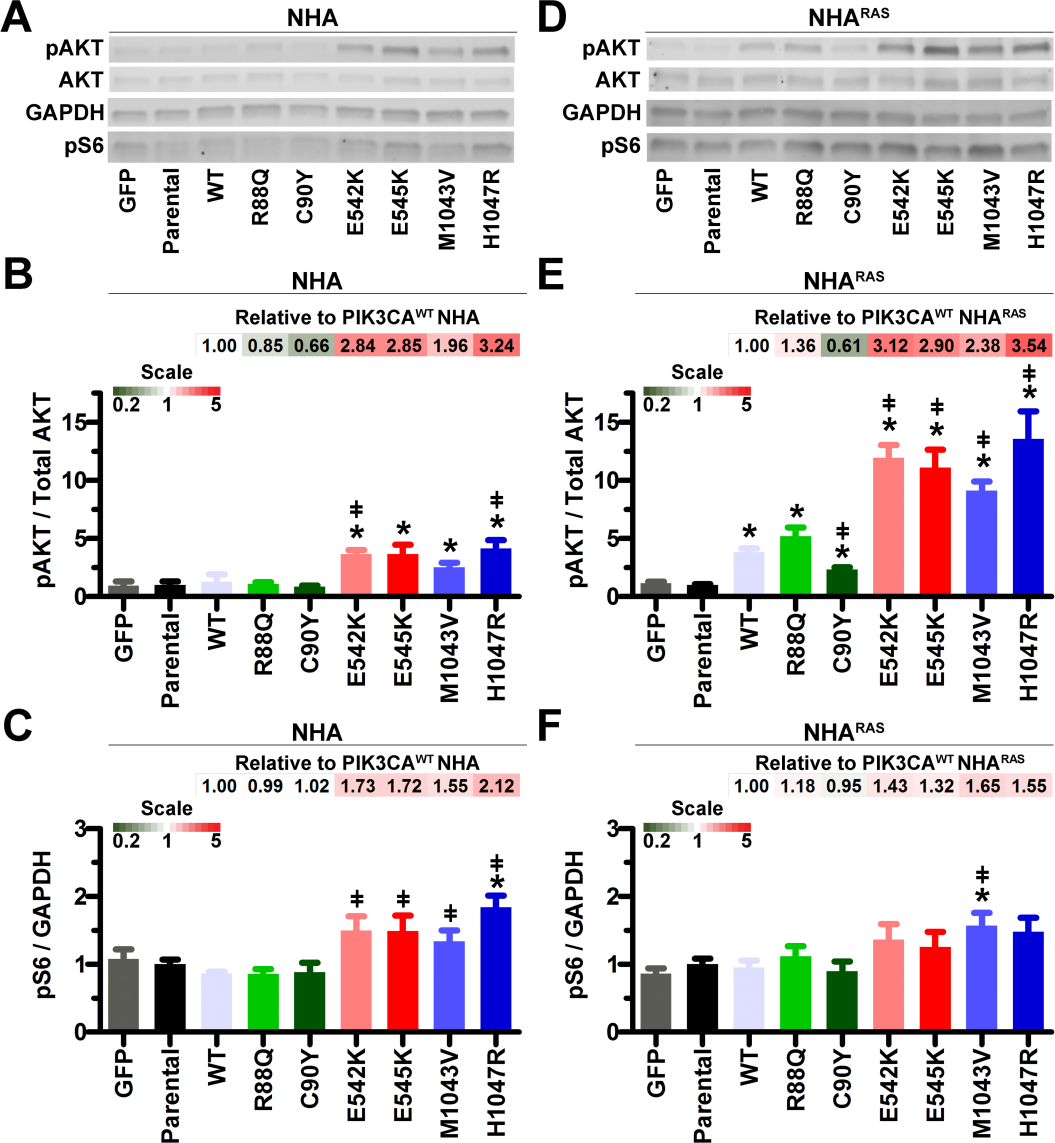
Helical and kinase *PIK3CA*^*mut*^ activate proximal PI3K. Representative immunoblots (**A**) and quantification showed that proximal PI3K (pAKT) was increased by helical and kinase mutants in NHA (**B**). Distal PI3K (pS6) was only increased by H1047R (**C**) (*, P≤0.02). E542K and H1047R increased proximal PI3K and all helical and kinase mutants increased distal PI3Kcompared to *PIK3CA*^*WT*^ NHA (ǂ, P≤0.04). Representative immunoblots (**D**) and quantification showed that proximal PI3K was increased by *PIK3CA*^*WT*^ and all mutants (**E**). Helical and kinase mutants also increased proximal PI3K compared to *PIK3CA*^*WT*^ *NHA*^*RAS*^ (ǂ, P≤0.007). Only M1043V increased distal PI3K compared to parental (*, P = 0.03) and *PIK3CA*^*WT*^ (ǂ, P = 0.03) NHA^RAS^ (**F**). Bar graph data are set relative to parental lines (N = 4 biologic replicates). Fold changes in pAKT and pS6 relative to *PIK3CA*^*WT*^ are shown as heatmaps.

Expression of *PIK3CA*^*WT*^ and all 6 *PIK3CA*^*mut*^ was similar in both NHA and NHA^RAS^ (**S2 Fig**), suggesting that phenotypic differences would be attributable to *PIK3CA*^*mut*^ examined. Neither *PIK3CA*^*WT*^ nor ABD *PIK3CA*^*mut*^ significantly activated proximal (pAKT) or distal (pS6) PI3K in serum-starved NHA (**Fig 1A–1C**). In contrast, E542K and H1047R significantly induced proximal and all 4 helical/kinase mutations induced distal PI3K.

We previously showed that mutant *Kras* cooperates with *Pten* deletion to activate PI3K in immortalized mouse astrocytes [19]. Therefore, we also determined the effects of *PIK3CA*^*mut*^ on PI3K in NHA^RAS^. *PIK3CA*^*WT*^ and all *PIK3CA*^*mut*^ increased proximal PI3K compared to parental NHA^RAS^ (**Fig 1D and 1E**). Furthermore, helical and kinase *PIK3CA*^*mut*^ potentiated proximal PI3K more than *PIK3CA*^*WT*^. However, distal PI3K was only increased by M1043V in NHA^RAS^ (**Fig 1D and 1F**). Taken together, these results suggest that mutant *RAS* cooperated with ectopic expression of both *PIK3CA*^*WT*^ and *PIK3CA*^*mut*^ to increase proximal PI3K.

There is extensive cross-talk between PI3K and MAPK pathways [44]. We therefore determined the effects of *PIK3CA*^*mut*^ on MAPK. Neither *PIK3CA*^*WT*^ nor any of the *PIK3CA*^*mut*^ significantly altered MAPK (pERK1/2) in NHA and NHA^RAS^ (**S3 Fig**). Thus, *PIK3CA*^*mut*^ activated PI3K without affecting the MAPK pathway.

### *PIK3CA*^mut^ induce NHA proliferation *in vitro*

**Fig 2:**
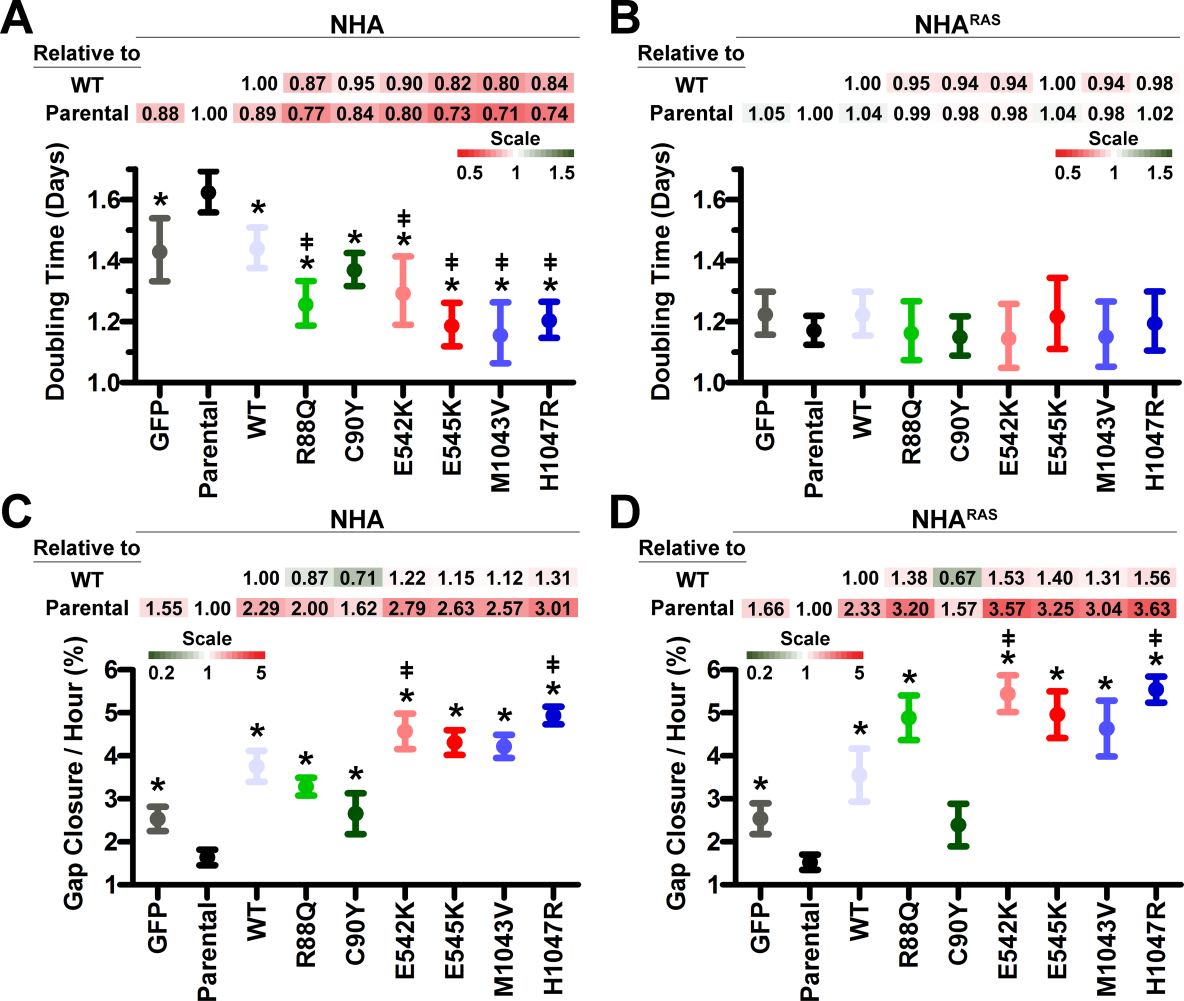
*PIK3CA*^*mut*^ potentiate proliferation and migration *in vitro*. MTS assays showed that *PIK3CA*^*WT*^ and all *PIK3CA*^*mut*^ decreased doubling times of NHA (**A**), but not NHA^RAS^ (**B**) (*, P≤0.02 vs parental, **S5A and S5B Fig**). *PIK3CA*^*mut*^, except C90Y, decreased doubling times compared to *PIK3CA*^*WT*^ NHA (^ǂ^, P≤0.03). Growth rates were analyzed by comparing k values. Error bars are 95% confidence intervals. *PIK3CA*^*WT*^ and *PIK3CA*^*mut*^, except C90Y, increased migration of both NHA (**C**) and NHA^RAS^ (**D**) (*, P≤0.04, **S5C and S5D Fig**). E542K and H1047R also potentiated migration compared to *PIK3CA*^*WT*^ NHA and NHA^RAS^ (^ǂ^, P≤0.005). Fold changes in doubling times and migration rates relative to parental and *PIK3CA*^*WT*^ lines are shown as heatmaps.

*PIK3CA*^*mut*^_,_ particularly helical and kinase mutants, activated PI3K, suggesting that they may also promote cell growth (**Fig 1**). MTS assays using high-serum (10% FBS) showed that *PIK3CA*^*WT*^ and a subset of *PIK3CA*^*mut*^ slightly increased growth rate (≤15%) of rapidly-proliferating NHA (doubling times ≤ 1 day; **S4 Fig**). We therefore hypothesized that growth factor concentrations in high-serum media were masking the effects of *PIK3CA*^*mut*^ on NHA growth. Indeed, MTS assays using low-serum (2.5% FBS) revealed increased proliferation of both GFP and *PIK3CA*^*WT*^ NHA compared to parental cells (**Fig 2A and S5A Fig**). While all *PIK3CA*^*mut*^ except C90Y increased proliferation compared to parental and *PIK3CA*^*WT*^ NHA, proliferation rates were similar in all NHA^RAS^ lines (**Fig 2B and S5B Fig**). Taken together, these data suggest that *PIK3CA*^*mut*^ promote astrocyte growth in the absence, but not presence, of mutant *RAS*.

### *PIK3CA*^mut^ induce migration *in vitro*

Complete surgical resection of GBM is precluded by its diffuse brain infiltration [45]. The PI3K pathway has an established role in migration [46]. We previously showed that *Pten* deletion increased migration of immortalized mouse astrocytes [19]. We therefore determined the effects of *PIK3CA*^*mut*^ on migration of NHA and NHA^RAS^ using an *in vitro* gap closure assay. GFP, *PIK3CA*^*WT*^ and all *PIK3CA*^*mut*^ showed increased migration compared to parental NHA (**Fig 2C and S5C Fig**). *PIK3CA*^*WT*^ and *PIK3CA*^*mut*^ except C90Y migrated faster than GFP NHA (P≤0.04). Similarly, *PIK3CA*^*mut*^ except C90Y migrated faster than GFP and parental NHA^RAS^ (P≤0.006, **Fig 2D and S5D Fig**). Moreover, E542K and H1047R *PIK3CA*^*mut*^ migrated faster than NHA and NHA^RAS^ overexpressing *PIK3CA*^*WT*^ (**Fig 2C and 2D**).

### *PIK3CA*^mut^ potentiate cellular transformation and tumorigenesis

**Fig 3:**
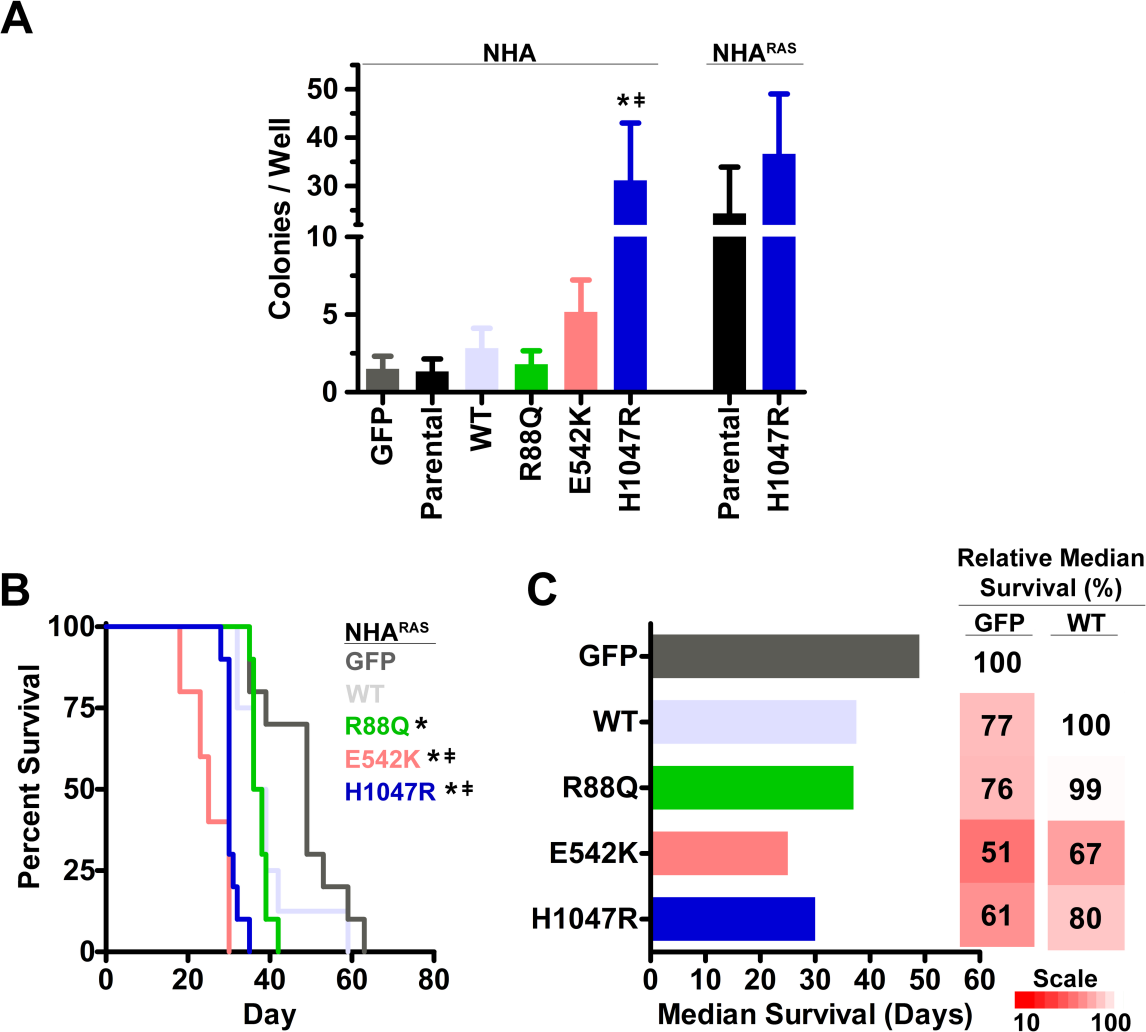
Helical and kinase *PIK3CA*^*mut*^ potentiate cellular transformation and tumorigenesis. Only H1047R increased colony formation compared to parental (*, P = 0.03) and *PIKCA*^*WT*^ (ǂ, P = 0.04) NHA (**A**). H1047R did not affect colony formation of NHA^RAS^ (P = 0.5). Orthotopic xenografts of GFP, *PIK3CA*^*WT*^, and *PIK3CA*^*mut*^ NHA^RAS^ (**BC**). Median survival of mice with R88Q, E542K, or H1047R *PIK3CA*^*mut*^ NHA^RAS^ was decreased compared to GFP control tumors (*, P≤0.003). E542K and H1047R *PIK3CA*^*mut*^ also decreased survival compared to *PIK3CA*^*WT*^ (ǂ, P≤0.002) and R88Q *PIK3CA*^*mut*^ (P<0.0001). Fold changes in median survival relative to GFP and *PIK3CA*^*WT*^ NHA^RAS^ are shown as heatmaps.

Anchorage-independent growth (colony formation in soft agar) is an established marker of cellular transformation [25, 41]. NHA^RAS^, but not NHA, form colonies *in vitro* and develop high-grade tumors in orthotopic mouse xenograft models [25]. We therefore first determined whether *PIK3CA*^*mut*^ promote NHA colony formation by selecting the most potent mutant in each domain (R88Q, E542K, H1047R) based on their effect on proximal PI3K, proliferation, and migration in NHA (**Figs 1 and 2**). Only H1047R induced colony formation relative to GFP and parental NHA (**Fig 3A**). Since this was the only mutation to potentiate NHA colony formation, we also tested its effect in NHA^RAS^. However, no significant increase in colony formation was evident (**Fig 3A**).

We next performed orthotopic xenografts of GFP, *PIK3CA*^*WT*^, and *PIK3CA*^*mut*^ in NHA^RAS^ to determine whether *PIK3CA*^*mut*^ potentiate tumorigenesis *in vivo*. Mice with R88Q, E542K, or H1047R *PIK3CA*^*mut*^ NHA^RAS^ tumors died more quickly than mice with control GFP tumors (P≤0.003). Additionally, mice with E542 or H1047R *PIK3CA*^*mut*^ tumors succumbed to disease more quickly than mice with tumors that overexpressed either *PIK3CA*^*WT*^ (P≤0.002) or R88Q *PIK3CA*^*mut*^ (P<0.0001; **Fig 3B and 3C**). Upon histopathological examination, tumor morphology was consistent across genotypes (**S6 Fig**). Taken together, these results indicate that both the mutated domain and concomitant mutant *RAS* influence the role of *PIK3CA*^*mut*^ in gliomagenesis *in vitro* and *in vivo*.

### PI3Ki efficacy is similar regardless of *PIK3CA*^*mut*^ status

**Fig 4:**
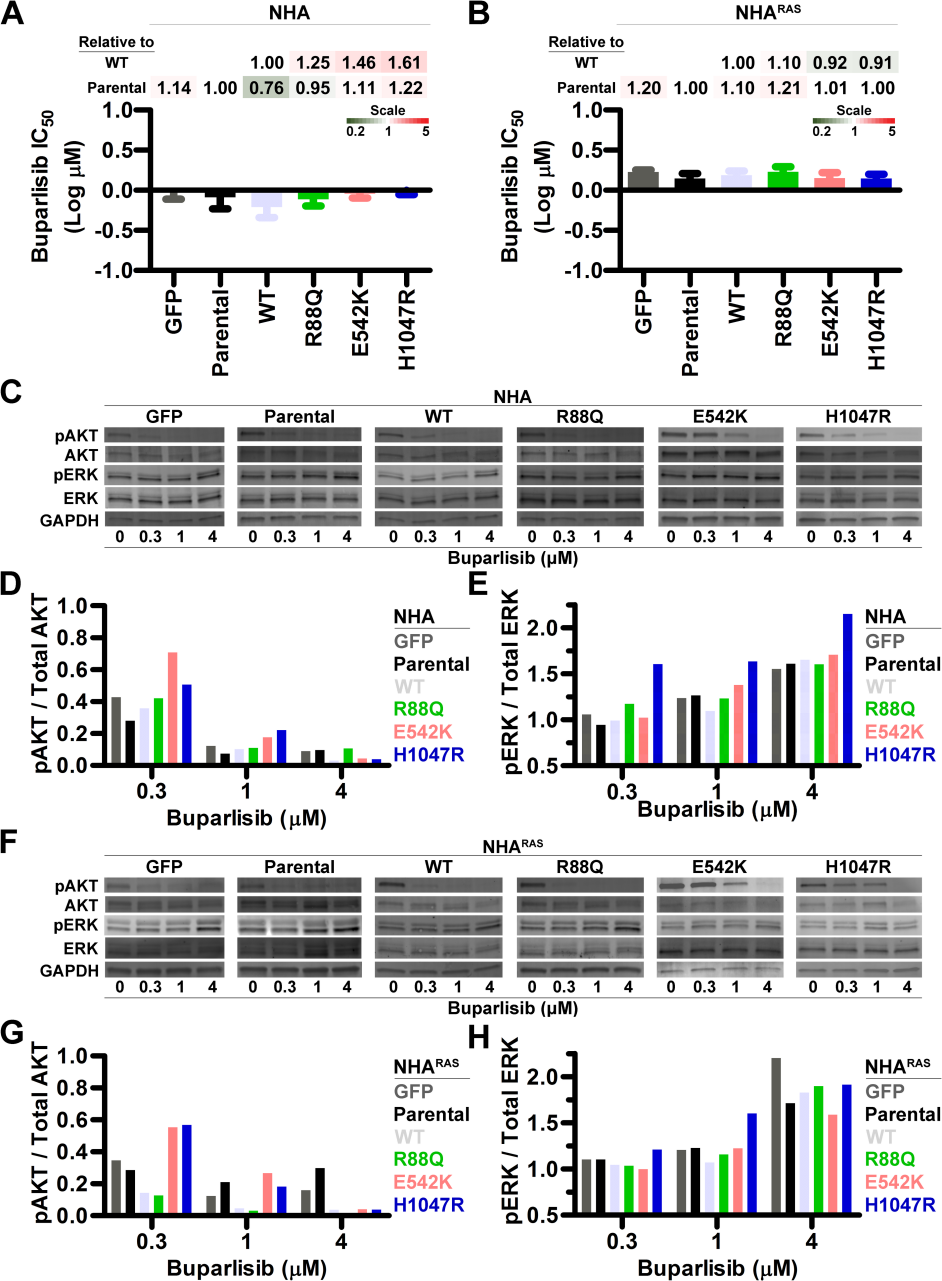
PI3Ki inhibits growth and ablates PI3K regardless of *PIK3CA*^*mut*^ status. Buparlisib IC_50_ were similar regardless of *PIK3CA*^*mut*^ status in NHA (**A**) and NHA^RAS^ (**B**) (**S7 Fig**). Fold changes in IC_50_ relative to parental and *PIK3CA*^*WT*^ are shown as heatmaps. Representative immunoblots of control and *PIK3CA*^*mut*^ NHA (**C**) and NHA^RAS^ (**F**) treated with buparlisib for 24 h. Immunoblot quantification (**DEGH**) demonstrated dose-dependent decreases in proximal PI3K (**DG**), with corresponding increases in MAPK (**EH**) in NHA (**DE**) and NHA^RAS^ (**GH**) lines (**S10 Fig**). Western blots were performed in either biological duplicates or triplicates.

Neuro-oncology is transitioning towards precision medicine, wherein tumor mutation profiles are utilized to tailor targeted treatments [8, 47]. Knowing which oncogenic driver mutations influence targeted inhibitor response is a prerequisite. To this end, we determined the effects of *PIK3CA*^*mut*^ on efficacy of the PI3Ki buparlisib *in vitro*. Buparlisib induced a dose-dependent decrease in growth of control and *PIK3CA*^*mut*^ NHA and NHA^RAS^ (**S7A and S7B Fig**). High nanomolar IC_50_ were evident regardless of the specific *PIK3CA*^*mut*^ (**Fig 4A**) but tended to be slightly higher in NHA^RAS^ (**Fig 4B and S7C Fig**). Buparlisib also induced G_2_/M cell cycle arrest in NHA^RAS^ lines regardless of *PIK3CA*^*mut*^ status (**S8 Fig**).

### PI3Ki ablate PI3K and potentiate MAPK

*While PIK3CA*^*mut*^ did not alter PI3Ki efficacy in NHA and NHA^RAS^ *in vitro* (**Fig 4A and 4B**), they differentially activated PI3K (**Fig 1**). We therefore investigated whether *PIK3CA*^*mut*^ influence PI3Ki-induced changes in pathway signaling. Buparlisib inhibited proximal (**Fig 4C and 4D**) and distal (**S9 Fig**) PI3K dose-dependently in control and *PIK3CA*^*mut*^ NHA. We and others have shown that PI3Ki induce alternate MAPK activation in preclinical GBM models [35, 44, 48]. Indeed, buparlisib induced dose-dependent increases in MAPK in NHA, regardless of their *PIK3CA*^*mut*^ status (**Fig 4C and 4E**).

Mutant *RAS* cooperated with *PIK3CA*^*WT*^ and *PIK3CA*^*mut*^ to potentiate activation of proximal PI3K (**Fig 1**), so we investigated whether *RAS* status influences PI3Ki-induced changes in PI3K and MAPK signaling. Buparlisib induced dose-dependent PI3K inhibition and MAPK activation in both control and *PIK3CA*^*mut*^ NHA^RAS^ (**Fig 4F–4H and S9C and S9D and S10 Figs**). Proximal PI3K inhibition was least pronounced in helical and kinase *PIK3CA*^*mut*^ lines at low buparlisib concentrations, demonstrating that higher PI3Ki doses are required to ablate PI3K in the presence of *PIK3CA*^*mut*^ in NHA^RAS^. These results also indicate that *PIK3CA*^*mut*^ status does not influence alternate MAPK activation.

### MEKi efficacy is independent of *PIK3CA*^*mut*^ in NHA

**Fig 5:**
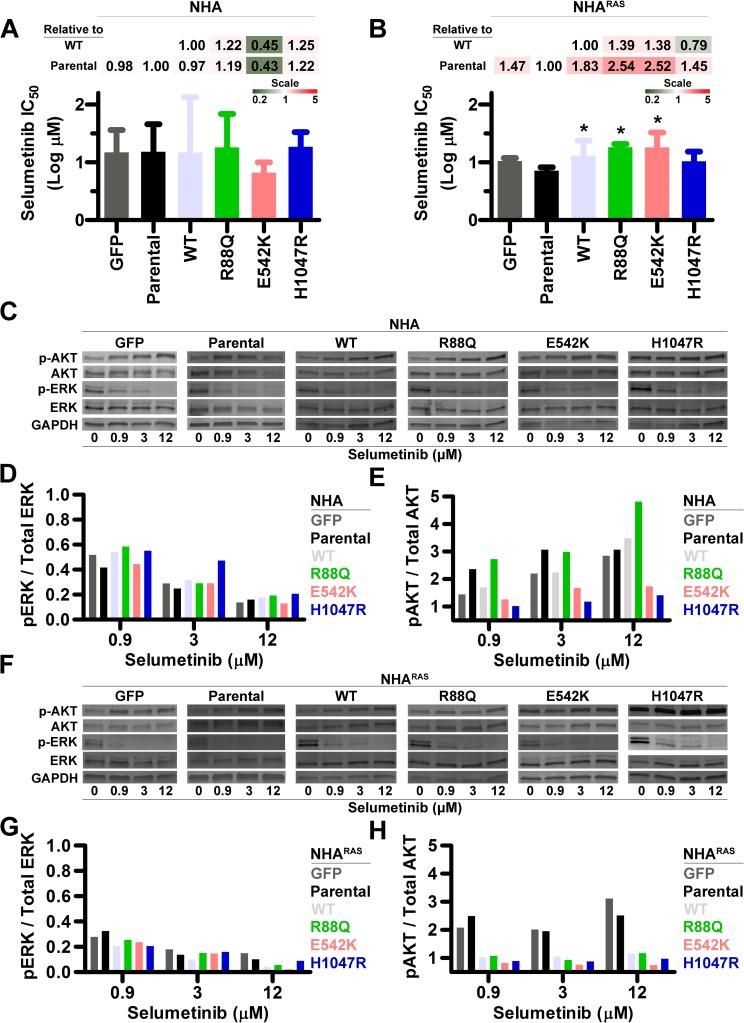
MEKi inhibits growth and ablates MAPK regardless of *PIK3CA*^*mut*^ status. Selumetinib IC_50_ were similar regardless of *PIK3CA*^*mut*^ status in NHA (**A**), but slightly higher in most *PIK3CA*^*mut*^ NHA^RAS^ compared to parental cells (*, P≤0.03) (**B**) (**S11 Fig**). Fold changes in IC_50_ relative to parental and *PIK3CA*^*WT*^ lines are shown as heatmaps. Representative immunoblots of control and *PIK3CA*^*mut*^ NHA (**C**) and NHA^RAS^ (**F**) treated with selumetinib for 24 h. Immunoblot quantification (**DEGH**) demonstrated dose-dependent decreases in MAPK in NHA (**D**) and NHA^RAS^ (**G**) lines. Although proximal PI3K was induced in control and *PIK3CA*^*mut*^ NHA (**E**), it was only potentiated in GFP and parental NHA^RAS^ (**H**) (**S12 Fig**). Western blots were performed either 1 or 2 times per experiment (Mean = 1.8).

Because PI3Ki promoted MAPK regardless of *PIK3CA*/*RAS* status, we determined efficacy of the MEKi selumetinib in control and *PIK3CA*^*mut*^ NHA and NHA^RAS^ lines *in vitro*. Selumetinib caused gradual, dose-dependent decreases in growth (**S11A and S11B Fig**) and had similar IC_50_ in both parental NHA and NHA^RAS^. While *PIK3CA*^*mut*^ status influenced IC_50_ in neither NHA (**Fig 5A**) nor most NHA^RAS^ lines, C90Y and H1047R were slightly more resistant than parental NHA^RAS^. (**Fig 5B and S11C Fig**). Thus, *PIK3CA*^*mut*^ and mutant *RAS* had little to no effect on MEKi sensitivity.

### *PIK3CA*^WT^ and *PIK3CA*^*mut*^ influence MEKi-induced PI3K activation in NHA^RAS^

Selumetinib inhibited MAPK in both control and *PIK3CA*^*mut*^ NHA (**Fig 5C**) and induced dose-dependent decreases in pERK regardless of *PIK3CA*^*mut*^ status (**Fig 5C and 5D**). We and others have shown that MEKi induces alternate PI3K activation in preclinical GBM models [35, 44, 49]. We extended these findings here, showing that selumetinib potentiated proximal PI3K 1.4-5-fold in control and *PIK3CA*^*mut*^ NHA (**Fig 5C and 5E**). Induction in NHA was least pronounced with E542K and H1047R, the mutations that most potentiated tumorigenesis in NHA^RAS^ (**Fig 3B and 3C**).

Mutant *RAS* cooperated with *PIK3CA*^*WT*^ and *PIK3CA*^*mut*^ to promote activation of proximal PI3K (**Fig 1**). We therefore investigated whether *PIK3CA*^*mut*^ influence MEKi-induced changes in MAPK and PI3K in NHA^RAS^. *PIK3CA*^*mut*^ status did not affect MAPK inhibition in selumetinib-treated NHA^RAS^ lines (**Fig 5F and 5G and S12A and S12B Fig**). Selumetinib induced alternate activation of proximal PI3K in GFP and parental NHA^RAS^ but ablated it in *PI3KCA*^*WT*^ and all *PIK3CA*^*mut*^ NHA^RAS^ (**Fig 5F and 5H and S12A and S12C Fig**). Taken together, these results indicate that ectopic *PIK3CA* expression in combination with mutant *RAS* prevents MEKi-induced alternate PI3K activation.

### Dual PI3Ki/MEKi treatment is synergistic in *PIK3CA*^*mut*^ NHA and NHA^RAS^

**Fig 6:**
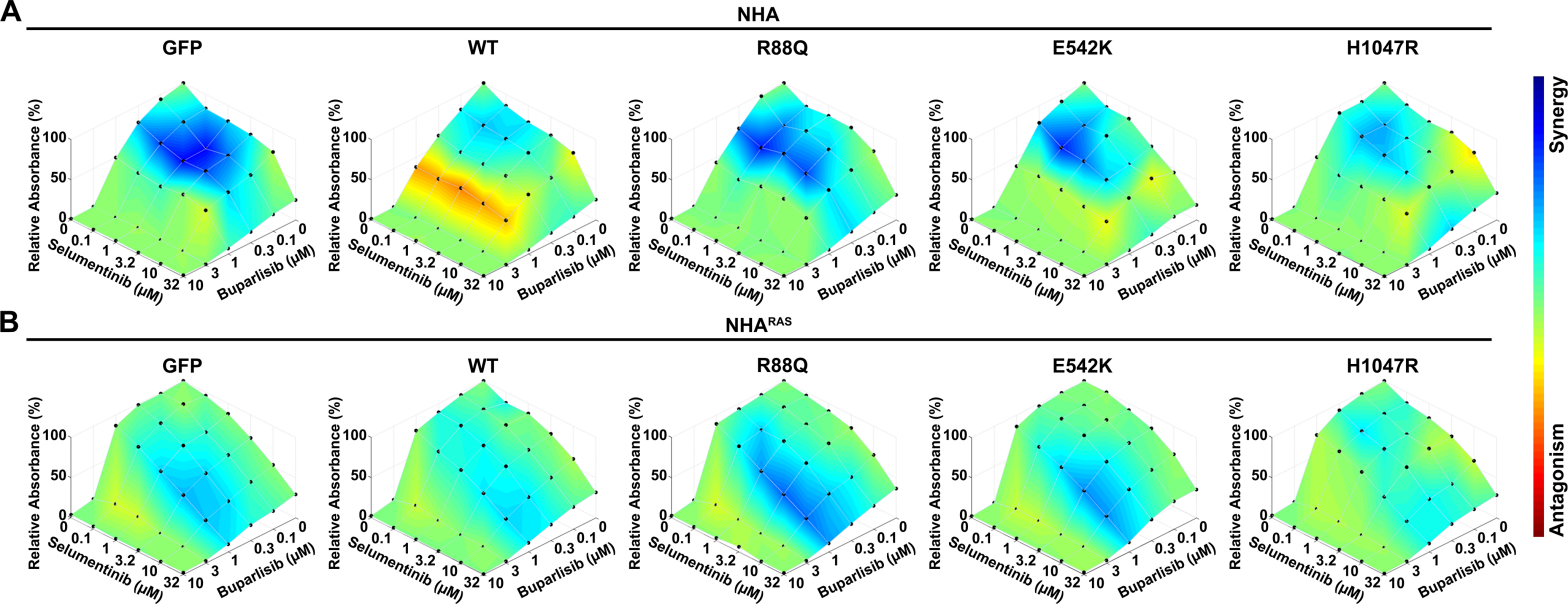
PI3Ki/MEKi synergism *in vitro* is influenced by *PIK3CA*^*mut*^ and mutant *RAS*. Buparlisib and selumetinib inhibited growth and were synergistic in control and PIK3CA^mut^ NHA (**A**) and NHA^RAS^ (**B**). BLISS showed that synergy was most pronounced with high nanomolar buparlisib and low micromolar/high nanomolar selumetinib in NHA lines. In contrast, synergistic concentrations in NHA^RAS^ lines were generally most pronounced at both low micromolar buparlisib and selumetinib.

We and others have shown that dual PI3Ki/MEKi efficacy is increased relative to treatment with either alone [35, 44, 48, 49]. However, the effects of GBM-associated mutations on PI3Ki/MEKi synergism remain unclear. To this end, we determined whether *PIK3CA*^*mut*^ influence the effects of dual PI3Ki/MEKi treatment on NHA and NHA^RAS^ growth *in vitro*. Buparlisib plus selumetinib synergistically inhibited growth in control and *PIK3CA*^*mut*^ NHA (**Fig 6A**). Synergy was highest in R88Q and E542K NHA relative to *PIK3CA*^*WT*^ and H1047R.

*PIK3CA*^*WT*^ and *PIK3CA*^*mut*^ marginally decreased MEKi efficacy in NHA^RAS^ (**Fig 5B and S11B and S11C Fig**). They also cooperated with mutant *RAS* to prevent MEKi-induced potentiation of proximal PI3K (**Fig 5F–5H and S12 Fig**). Therefore, both *PIK3CA*^*WT*^ and *PIK3CA*^*mut*^ may alter PI3Ki/MEKi synergism when combined with mutant *RAS*. Dual buparlisib/selumetinib treatment synergistically inhibited growth of all NHA^RAS^ lines (**Fig 6B**). However, synergism was most pronounced at higher drug concentrations in NHA^RAS^ relative to NHA lines. Furthermore, synergy was highest in R88Q, E542K, and M1043V mutant NHA^RAS^ (**S13 Fig**). Taken together, these data suggest that mutant *RAS* and *PIK3CA* alter PI3Ki/MEKi synergism.

## Discussion

### *PIK3CA*^*mut*^ differentially activate PI3K and promote gliomagenesis

The vast majority of GBM harbor mutations in core PI3K pathway genes and/or upstream RTK [4]. Activation of PI3K via *Pten* deletion, *PIK3R1* mutations, or constitutively active *AKT* mutants promotes tumorigenesis in glioma models [19–22]. Here, we determined the effects of ABD, helical, and kinase *PIK3CA*^*mut*^ on gliomagenesis. Both helical and kinase *PIK3CA*^*mut*^ potentiated PI3K, proliferation, and migration of NHA compared to parental and *PIK3CA*^*WT*^ lines (**Figs 1 and 2**), but only H1047R kinase mutation potentiated NHA colony formation (**Fig 3A**).

We and others have shown that PI3K activation via *Pten* deletion or constitutively active AKT cooperates with MAPK activation to potentiate gliomagenesis [19, 22–24]. We extended these findings here by demonstrating that mutant *RAS* promoted *PIK3CA*^*WT*^- and *PIK3CA*^*mut*^-induced proximal PI3K (**Fig 1**). Unlike in NHA, *PIK3CA*^*mut*^ did not increase proliferation of NHA^RAS^, likely due to the rapid proliferation rate of parental cells (**Fig 2A and 2B**). H1047R *PIK3CA*^*mut*^ also did not potentiate colony formation of NHA^RAS^, likely because NHA^RAS^ cells are more aggressive and form colonies more readily and at higher density than NHA cells (**Fig 3A**). However, the E542K and H1047R *PIK3CA*^*mut*^ potentiated malignancy of NHA^RAS^ *in vivo* compared to GFP and *PIK3CA*^*WT*^ (**Fig 3B and 3C**). These results are consistent with previous findings that constitutively active AKT does not enhance proliferation or colony formation of NHA^RAS^ *in vitro* but promotes tumorigenesis *in vivo* [22]. These data suggest that E542K and H1047R *PIK3CA*^*mut*^ promote *in vivo* gliomagenesis equally in the presence, but not absence, of mutant *RAS*.

In contrast to helical and kinase *PIK3CA* mutations, ABD *PIK3CA*^*mut*^ did not increase PI3K, migration, or colony formation of NHA more than *PIK3CA*^*WT*^ (**Figs 1–3**). Similar results were obtained with ABD *PIK3CA*^*mut*^ in NHA^RAS^. Moreover, R88Q *PIK3CA*^*mut*^ did not promote tumorigenesis of NHA^RAS^ more than *PIK3CA*^*WT*^ *in vivo* (**Fig 3B and 3C**). Taken together, these results demonstrate that the phenotypic consequences of ABD *PIK3CA*^*mut*^ and ectopic over-expression of *PIK3CA*^*WT*^ are similar. Furthermore, they suggest that ABD *PIK3CA*^*mut*^ may be passenger mutations in GBM. However, ectopic expression of *PIK3CA*^*WT*^ and *PIK3CA*^*mut*^ may not fully recapitulate the effects of *PIK3CA*^*mut*^ when expressed under its endogenous promoter. Furthermore, other cooperating mutations and/or cellular origin may influence the role of *PIK3CA*^*mut*^ in gliomagenesis. Future work will be required to investigate the role of *PIK3CA*^*mut*^ in other genetic and cellular contexts.

### *PIK3CA*^*mut*^ do not influence PI3Ki efficacy

The precision medicine initiative seeks to direct treatment with targeted inhibitors based on tumor mutation profiles [8, 47]. However, this requires an understanding of how oncogenic mutations influence drug response. Mutational activation of kinases can cause oncogene addiction, in which tumor cells become reliant upon the activated signaling pathway(s), and are thus highly sensitive to their inhibition [50]. Additionally, kinase mutations can alter drug affinity, thereby altering efficacy [51]. Buparlisib inhibits purified PIK3CA^WT^ and the most common PIK3CA^mut^, E542K, E545K, and H1047R, with similar IC_50_ [52, 53]. Because these helical and kinase domain *PIK3CA*^*mut*^ activated PI3K and promoted gliomagenesis, we hypothesized that they would also increase PI3Ki efficacy. However, higher buparlisib doses were required to ablate PI3K in cells expressing *PIK3CA*^*mut*^, particularly those in the helical and kinase domains, and these mutations did not influence PI3Ki efficacy *in vitro* (**Fig 4**). These results suggest that *PIK3CA*^*mut*^ neither induce oncogene addiction nor enhance PI3Ki sensitivity. Whether they influence efficacy of isoform-specific PI3Ki or inhibitors of downstream kinases, such as AKT and mTOR, remains to be determined.

### *PIK3CA*^*mut*^ influence MEKi-induced PI3K activation and PI3Ki/MEKi synergism

We previously found that buparlisib induced widespread kinome changes, including MAPK activation, in immortalized murine astrocytes with *Pten* deletion and mutant *Kras* [35]. We expanded these findings here by demonstrating that buparlisib potentiated MAPK regardless of *RAS*/*PIK3CA* mutation status (**Fig 4C–4H**). *PIK3CA*^*mut*^ also had minimal to no effect on sensitivity of NHA and NHA^RAS^ to MEKi *in vitro* (**Fig 5A and 5B**).

We and others have shown that MEKi promote PI3K in preclinical GBM models [35, 44, 49]. We found that selumetinib increased proximal PI3K in control and *PIK3CA*^*mut*^ NHA, and in GFP and parental NHA^RAS^ (**Fig 5C–5H**). Interestingly, this increase was not apparent in *PIK3CA*^*WT*^ and *PIK3CA*^*mut*^ NHA^RAS^ (**Fig 5F and 5H and S12A and S12C Fig**). The mechanism by which ectopic *PIK3CA* expression in combination with mutant *RAS* alters MEKi response is unclear. A mutually inhibitory crosstalk between PI3K and MAPK is mediated by p70S6K in glioma stem cells [44]. MAPK inhibition induces PI3K in non-GBM cell lines via removal of a negative feedback loop on RTK [54]. Similarly, selumetinib induces widespread kinome changes in breast cancer models, including increased expression and activity of multiple RTK [55]. Taken together, these results suggest that *PIK3CA*^*WT*^ and *PIK3CA*^*mut*^ may cooperate with mutant *RAS* to alter MEKi-induced dynamic kinome changes, particularly as it pertains to PI3K activation.

Dual PI3Ki/MEKi treatment is effective in multiple preclinical GBM models [35, 44, 48, 49]. It remains unclear whether the underlying genetics of these models influence drug synergism. We therefore determined if *PIK3CA*^*mut*^ affected PI3Ki/MEKi synergism in the presence and absence of mutant *RAS*. Consistent with other GBM models, we found that dual buparlisib/selumetinib treatment was synergistic in NHA and NHA^RAS^ lines (**Fig 6**). However, *RAS*/*PIK3CA*^*mut*^ status influenced drug response. Higher concentrations of buparlisib and selumetinib were required to maximize synergism in NHA^RAS^ lines compared to NHA. Furthermore, synergy was generally greater in R88Q and E542K NHA and NHA^RAS^ compared to those with either *PIK3CA*^*WT*^ or H1047R. Taken together, these results suggest that GBM patients with helical *PIK3CA*^*mut*^ may be most sensitive to dual PI3Ki/MEKi treatment. Given that ABD *PIK3CA*^*mut*^ showed no significant tumorigenic effects *in vitro* and *in vivo*, their utility in predicting PI3Ki/MEKi synergy remains questionable.

## Conclusion

Defining the role of frequently occurring mutations in GBM pathogenesis and drug response can aid identification of predictive biomarkers. Our results demonstrate that *PIK3CA*^*mut*^ differentially promote gliomagenesis and do not predict PI3Ki sensitivity but do impact PI3Ki/MEKi synergism.

## Supporting information

S1 Fig Frequency and distribution of *PIK3CA*^*mut*^. Lollipop plot of *PIK3CA* missense (green), in-frame deletion (brown), and truncating mutations (black) in GBM (**A**) and all published TCGA datasets (**B**). *PIK3CA* missense mutations investigated here are indicated. PIK3CA point mutations were evident in 10.3% of GBM cases from the TCGA (N = 273), with each mutation investigated here representing ≤1% of total mutations. Data were downloaded from cBioPortal (http://www.cbioportal.org/) on March 10, 2017. Ribbon diagram of *PIK3CA* with mutations investigated highlighted (**C**) (R88Q = light green; C90Y = dark green; E542K = pink; E545K = red; M1043V = purple; H1047R = blue). Model was generated in PyMOL. (Schrödinger, New York City, NY) using a script downloaded from cBioPortal.^13,14^
(TIF)

S2 Fig *PIK3CA*^*WT*^ and *PIK3CA*^*mut*^ are expressed at similar levels. Representative immunoblots (**AC**) and quantification (**BD**) of HA-tagged PIK3CA showed that *PIK3CA*^*WT*^ and *PIK3CA*^*mut*^ were expressed at similar levels in NHA (**BC**) and NHA^RAS^ (**CD**) (ANOVA, P≥0.3). Bar graph data were set relative to *PIK3CA*^*WT*^ lines (N = 3–4 biologic replicates, Mean = 3.5).
(TIF)

S3 Fig *PIK3CA*^*mut*^ do not alter MAPK. Representative immunoblots (**AC**) and quantification (**BD**) showed that *PIK3CA*^mut^ did not alter MAPK (phosphorylation of ERK1/2, pERK) in either NHA (**AB**) or NHA^RAS^ (**CD**) (P≥0.93). Bar graph data were set relative to parental lines (N = 3–4 biologic replicates, Mean = 3.5). Fold changes in pERK relative to PIK3CA^WT^ lines are shown as heatmaps.
(TIF)

S4 Fig *PIK3CA*^*mut*^ have minor effects on growth in high-serum culture. MTS assays (**A**) showed that *PIK3CA*^*WT*^ and a subset of *PIK3CA*^*mut*^ slightly increased growth (reduced doubling times) compared to parental NHA (*, P≤0.03) when grown in media containing high (10%) FBS. (**B**). R88Q growth was slightly slower than *PIK3CA*^*WT*^ NHA (ǂ, P = 0.01). Statistical analyses of growth rates were performed by comparing k values. Fold changes in doubling times relative to parental and *PIK3CA*^*WT*^ lines are shown as heatmaps. Error bars in **B** are 95% confidence intervals.
(TIF)

S5 Fig Influence of *PIK3CA*^*mut*^ on proliferation, but not migration, is dependent on mutant *RAS in vitro*. Growth of control and *PIK3CA*^*mut*^ NHA (**A**) and NHA^RAS^ (**B**) (**Fig 2A and 2B**). Growth was determined by assessing changes in relative absorbance daily by MTS. Migration of control and *PIK3CA* mutant NHA (**C**) and NHA^RAS^ (**D**) across a gap (**Fig 2C and 2D**).
(TIF)

S6 Fig Histopathologic features of malignancy are consistent across genotypes. Hematoxylin and eosin staining of tumors from *PIK3CA*^*WT*^ (**A**), R88Q (**B**), E542K (**C**), and H1047R (**D**) *PIK3CA*^*mut*^ mice revealed malignant histopathologic features typical of human gliomas, including cytologic and nuclear atypia, tumor giant cells, and mitotic figures (white arrows). Scale bar = 100 μm.
(TIF)

S7 Fig PI3Ki inhibits growth independent of *PIK3CA*^*mut*^ status *in vitro*. MTS assays showed that buparlisib caused dose-dependent decreases in growth of control and *PIK3CA*^*mut*^ NHA (**A**) and NHA^RAS^ (**B**). Buparlisib IC_50_ were similar between control and all 6 *PIK3CA*^*mut*^ NHA^RAS^ (**C**).
(TIF)

S8 Fig PI3Ki induces G_2_/M cell cycle arrest in NHA^RAS^ cells regardless of *PIK3CA*^*mut*^ status. Micromolar doses of buparlisib induced G_2_/M cell cycle arrest within 48 h in control and PIK3CA^mut^ NHA^RAS^.
(TIF)

S9 Fig PI3Ki inhibits distal PI3K regardless of *PIK3CA*^*mut*^ status. Representative immunoblots of control and *PIK3CA*^*mut*^ NHA (**A**) and NHA^RAS^ (**C**) 24 h after buparlisib treatment. Immunoblot quantification demonstrated dose-dependent inhibition of distal PI3K in all NHA (**B**) and NHA^RAS^ (**D**) lines (N = 1–3 biologic replicates, Mean = 1.7).
(TIF)

S10 Fig PI3Ki inhibits proximal PI3K signaling and induces MAPK signaling in all control and *PIK3CA*^*mut*^ NHA^RAS^. Representative immunoblots (**A**) and quantification of proximal PI3K (**B**) and MAPK (**C**) showed that within 24 h, buparlisib induced dose-dependent inhibition of PI3K signaling, with concurrent induction of MAPK in parental, GFP, PIK3CA^WT^, and all 6 PIK3CA^mut^ NHA^RAS^ lines (N = 2–3 biologic replicates, Mean = 2.7).
(TIF)

S11 Fig MEKi inhibits *in vitro* growth in all *PIK3CA*^*mut*^ lines. MTS assays showed that selumetinib caused dose-dependent decreases in growth of control and *PIK3CA*^*mut*^ NHA (**A**) and NHA^RAS^ (**B**). Selumetinib IC_50_ were slightly increased by *PIK3CA*^*WT*^ and all *PIK3CA*^*mut*^, except C90Y and H1047R, compared to parental NHA^RAS^ (*, P≤0.03) (**C**).
(TIF)

S12 Fig MEKi induction of proximal PI3K signaling is abrogated in *PIK3CA*^*WT*^ and *PIK3CA*^*mut*^ NHA^RAS^. Representative immunoblots (**A**) and quantification of MAPK (**B**) and proximal PI3K (**C**) showed that selumetinib caused dose-dependent inhibition of MAPK regardless of *PIKCA*^*WT*^ or *PIK3CA*^*mut*^ status, but concurrent induction of proximal PI3K only occurred in parental and GFP NHA^RAS^ (N = 2–3 biologic replicates, Mean = 2.7).
(TIF)

S13 Fig PI3K/MEKi synergism is differentially influenced by *PIK3CA*^*mut*^ *in vitro*. Buparlisib and selumetinib were synergistic in parental and *PIK3CA*^*mut*^ NHA^RAS^ (**Fig 6B**)
(TIF)

S14 Fig Supporting information supplement. (DOCX)

S15 Fig Supplementary immunoblots. (PPTX)
