# Supplementary figures and images for: PIK3CA missense mutations promote glioblastoma pathogenesis, but do not enhance targeted PI3K inhibition
Source: PLoS One. 2018 Jul 5;13(7):e0200014. doi: 10.1371/journal.pone.0200014 (PMC6033446; doi:10.1371/journal.pone.0200014)

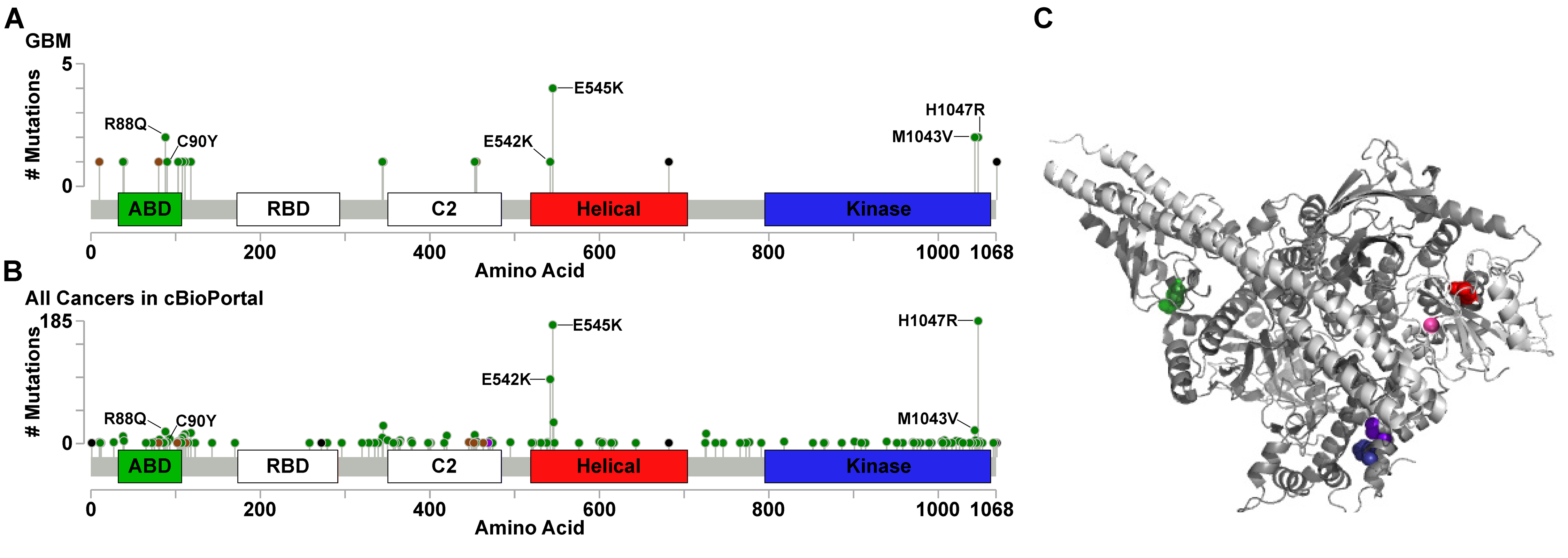

Supplement: S1 Fig — Lollipop plot of PIK3CA missense (green), in-frame deletion (brown), and truncating mutations (black) in GBM (A) and all published TCGA datasets (B). PIK3CA missense mutations investigated here are indicated. PIK3CA point mutations were evident in 10.3% of GBM cases from the TCGA (N = 273), with each mutation investigated here representing ≤1% of total mutations. Data were downloaded from cBioPortal (http://www.cbioportal.org/) on March 10, 2017. Ribbon diagram of PIK3CA with mutations investigated highlighted (C) (R88Q = light green; C90Y = dark green; E542K = pink; E545K = red; M1043V = purple; H1047R = blue). Model was generated in PyMOL. (Schrödinger, New York City, NY) using a script downloaded from cBioPortal.13,14 (TIF) [file pone.0200014.s001.tif]

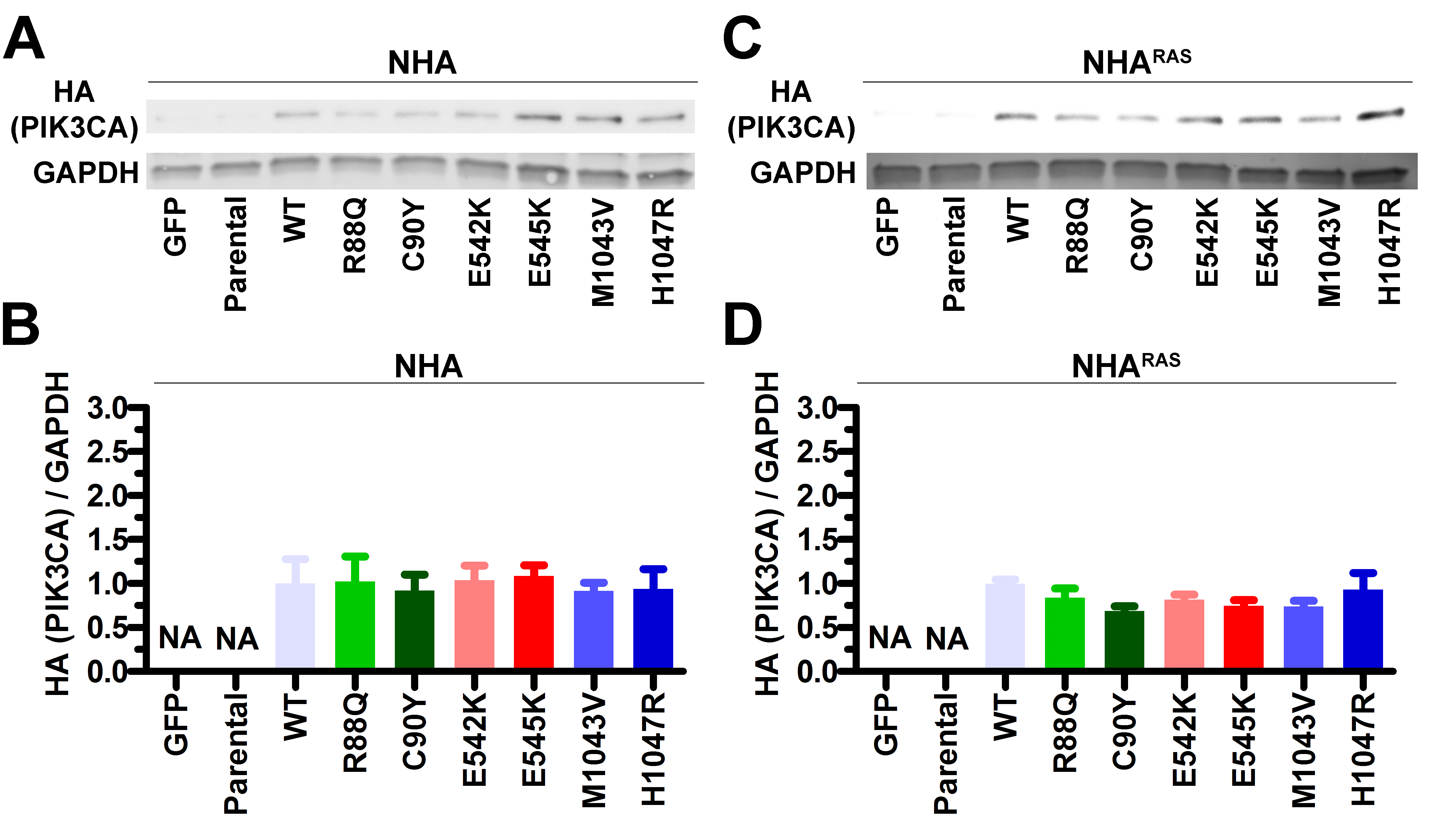

Supplement: S2 Fig — Representative immunoblots (AC) and quantification (BD) of HA-tagged PIK3CA showed that PIK3CAWT and PIK3CAmut were expressed at similar levels in NHA (BC) and NHARAS (CD) (ANOVA, P≥0.3). Bar graph data were set relative to PIK3CAWT lines (N = 3–4 biologic replicates, Mean = 3.5). (TIF) [file pone.0200014.s002.tif]

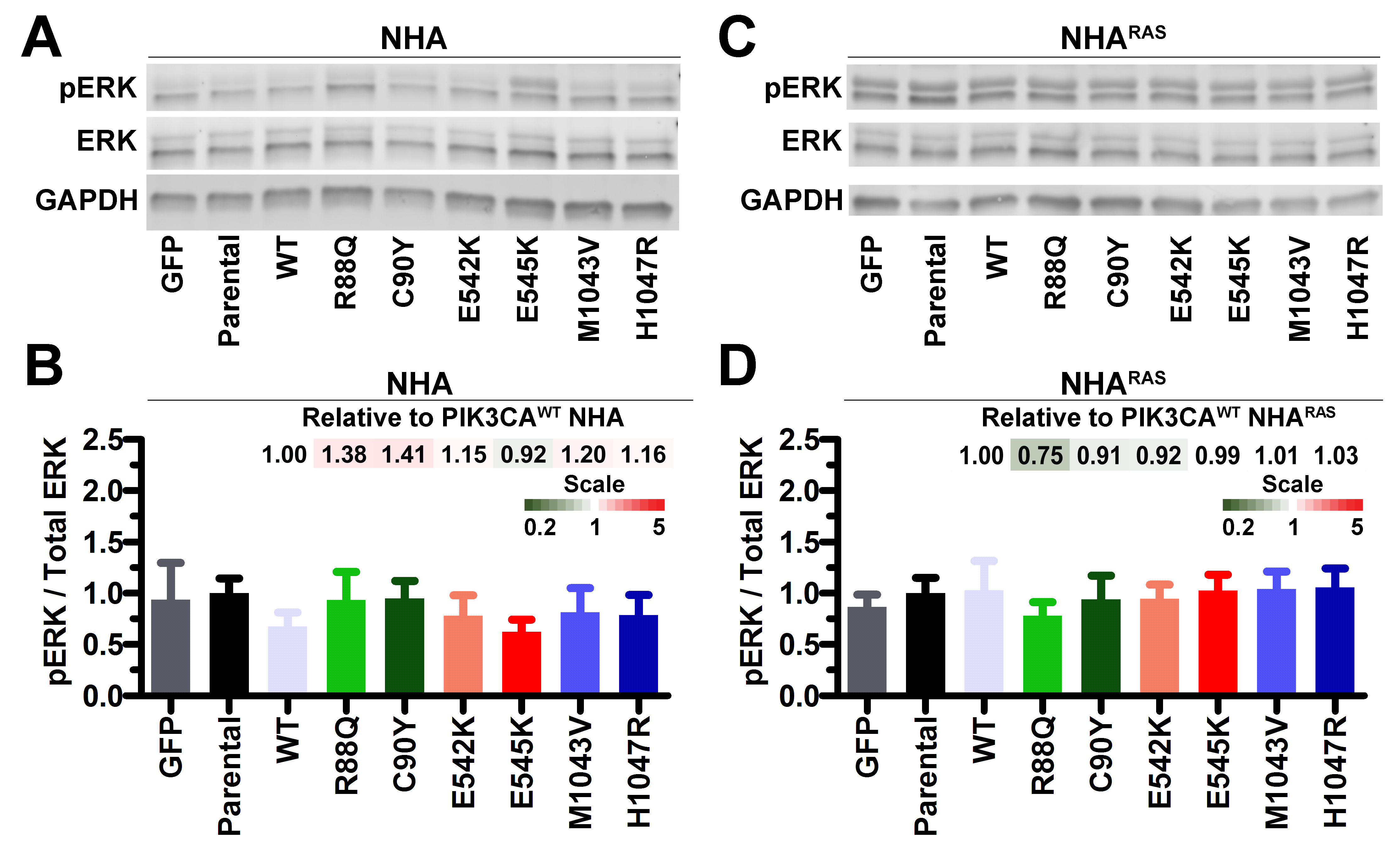

Supplement: S3 Fig — Representative immunoblots (AC) and quantification (BD) showed that PIK3CAmut did not alter MAPK (phosphorylation of ERK1/2, pERK) in either NHA (AB) or NHARAS (CD) (P≥0.93). Bar graph data were set relative to parental lines (N = 3–4 biologic replicates, Mean = 3.5). Fold changes in pERK relative to PIK3CAWT lines are shown as heatmaps. (TIF) [file pone.0200014.s003.tif]

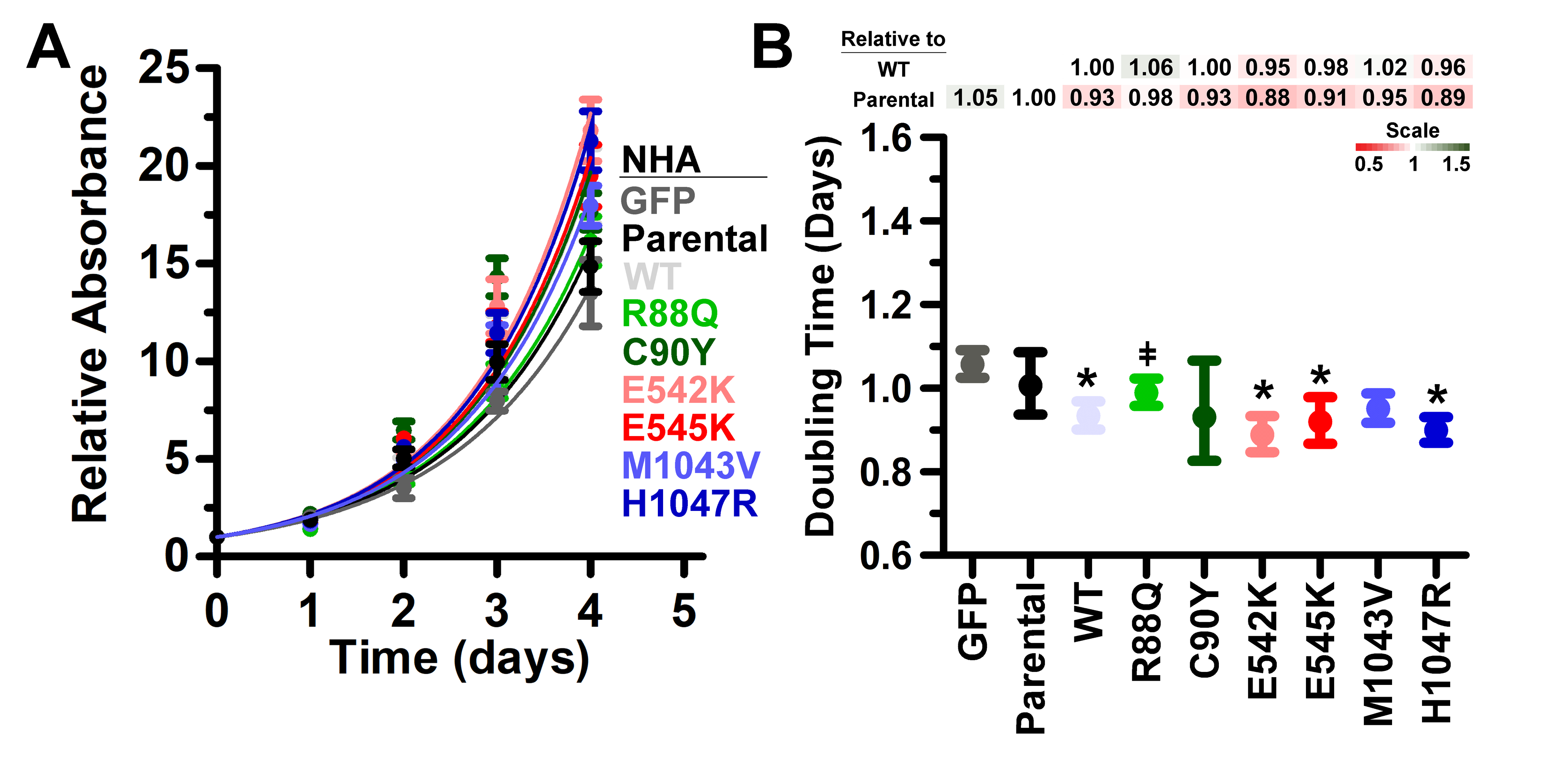

Supplement: S4 Fig — MTS assays (A) showed that PIK3CAWT and a subset of PIK3CAmut slightly increased growth (reduced doubling times) compared to parental NHA (*, P≤0.03) when grown in media containing high (10%) FBS. (B). R88Q growth was slightly slower than PIK3CAWT NHA (ǂ, P = 0.01). Statistical analyses of growth rates were performed by comparing k values. Fold changes in doubling times relative to parental and PIK3CAWT lines are shown as heatmaps. Error bars in B are 95% confidence intervals. (TIF) [file pone.0200014.s004.tif]

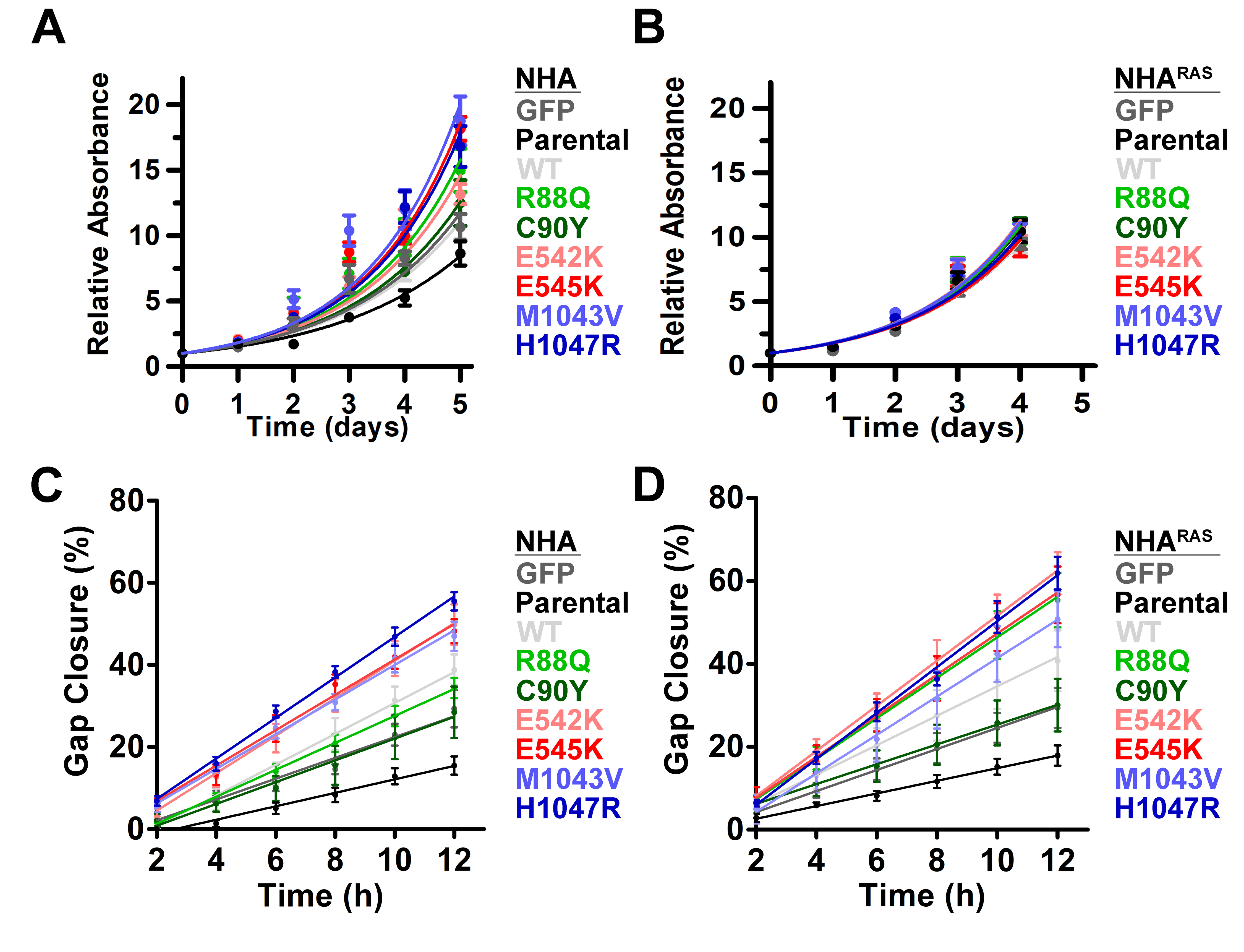

Supplement: S5 Fig — Growth of control and PIK3CAmut NHA (A) and NHARAS (B) (Fig 2A and 2B). Growth was determined by assessing changes in relative absorbance daily by MTS. Migration of control and PIK3CA mutant NHA (C) and NHARAS (D) across a gap (Fig 2C and 2D). (TIF) [file pone.0200014.s005.tif]

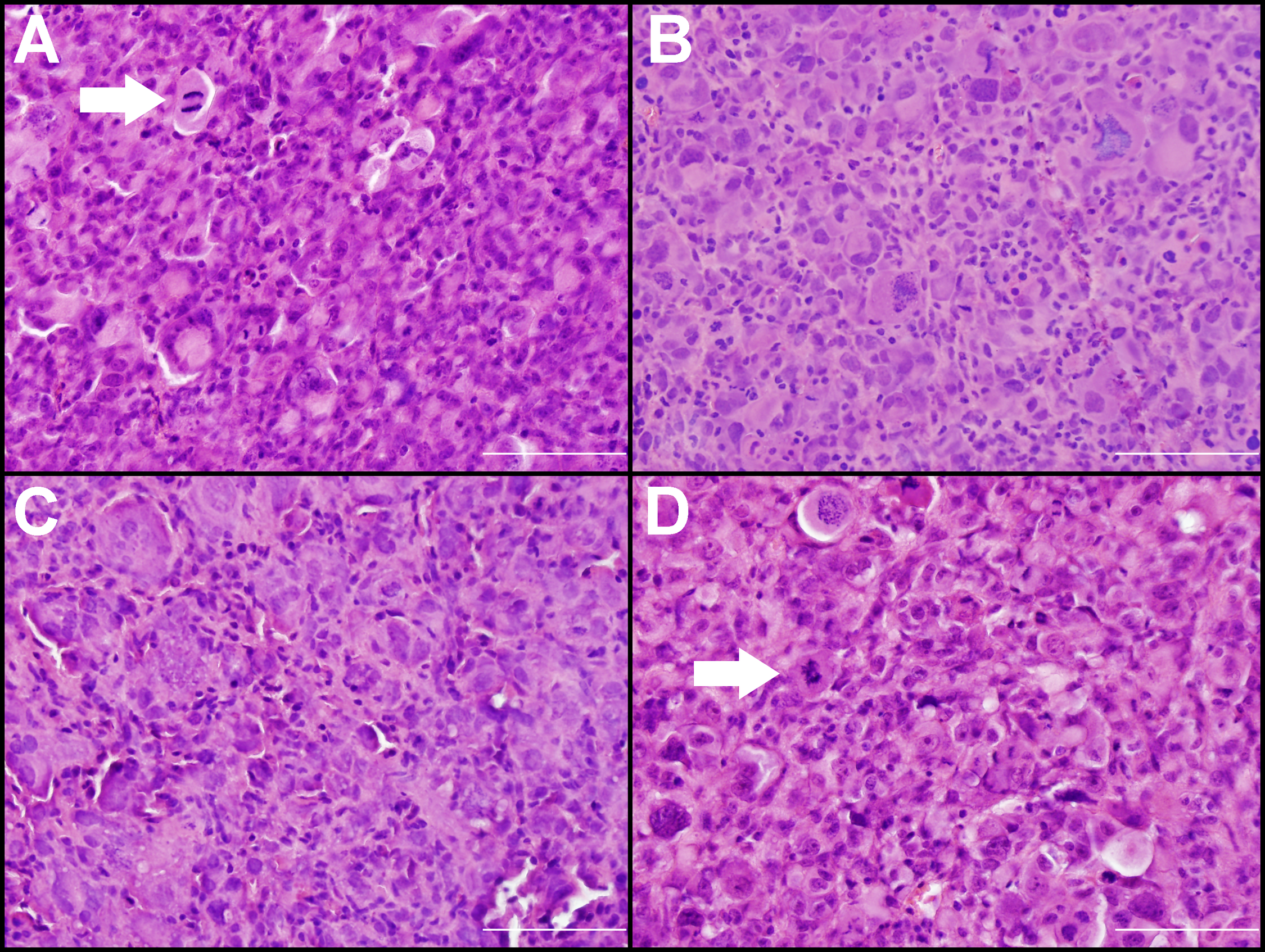

Supplement: S6 Fig — Hematoxylin and eosin staining of tumors from PIK3CAWT (A), R88Q (B), E542K (C), and H1047R (D) PIK3CAmut mice revealed malignant histopathologic features typical of human gliomas, including cytologic and nuclear atypia, tumor giant cells, and mitotic figures (white arrows). Scale bar = 100 μm. (TIF) [file pone.0200014.s006.tif]

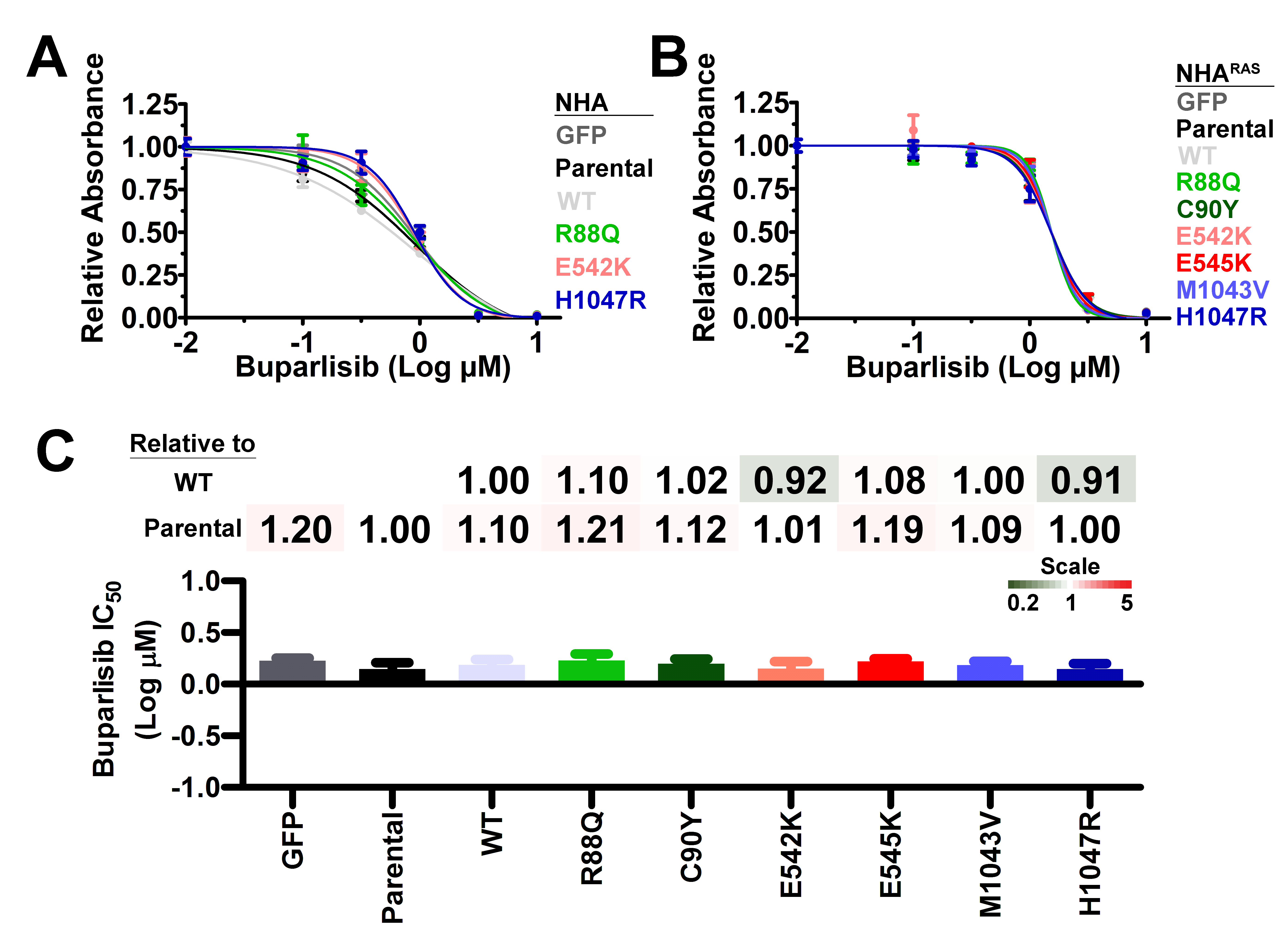

Supplement: S7 Fig — MTS assays showed that buparlisib caused dose-dependent decreases in growth of control and PIK3CAmut NHA (A) and NHARAS (B). Buparlisib IC50 were similar between control and all 6 PIK3CAmut NHARAS (C). (TIF) [file pone.0200014.s007.tif]

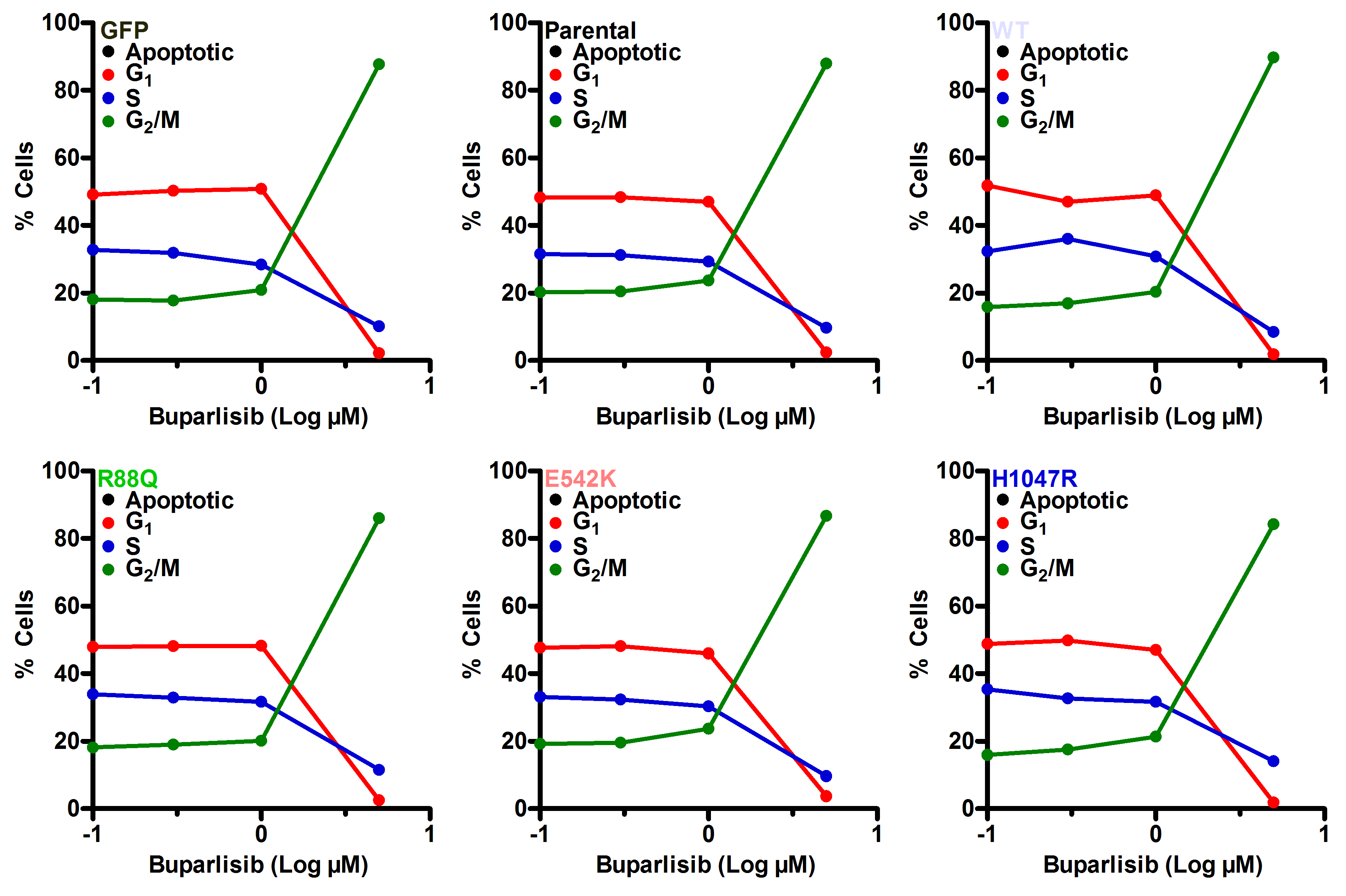

Supplement: S8 Fig — Micromolar doses of buparlisib induced G2/M cell cycle arrest within 48 h in control and PIK3CAmut NHARAS. (TIF) [file pone.0200014.s008.tif]

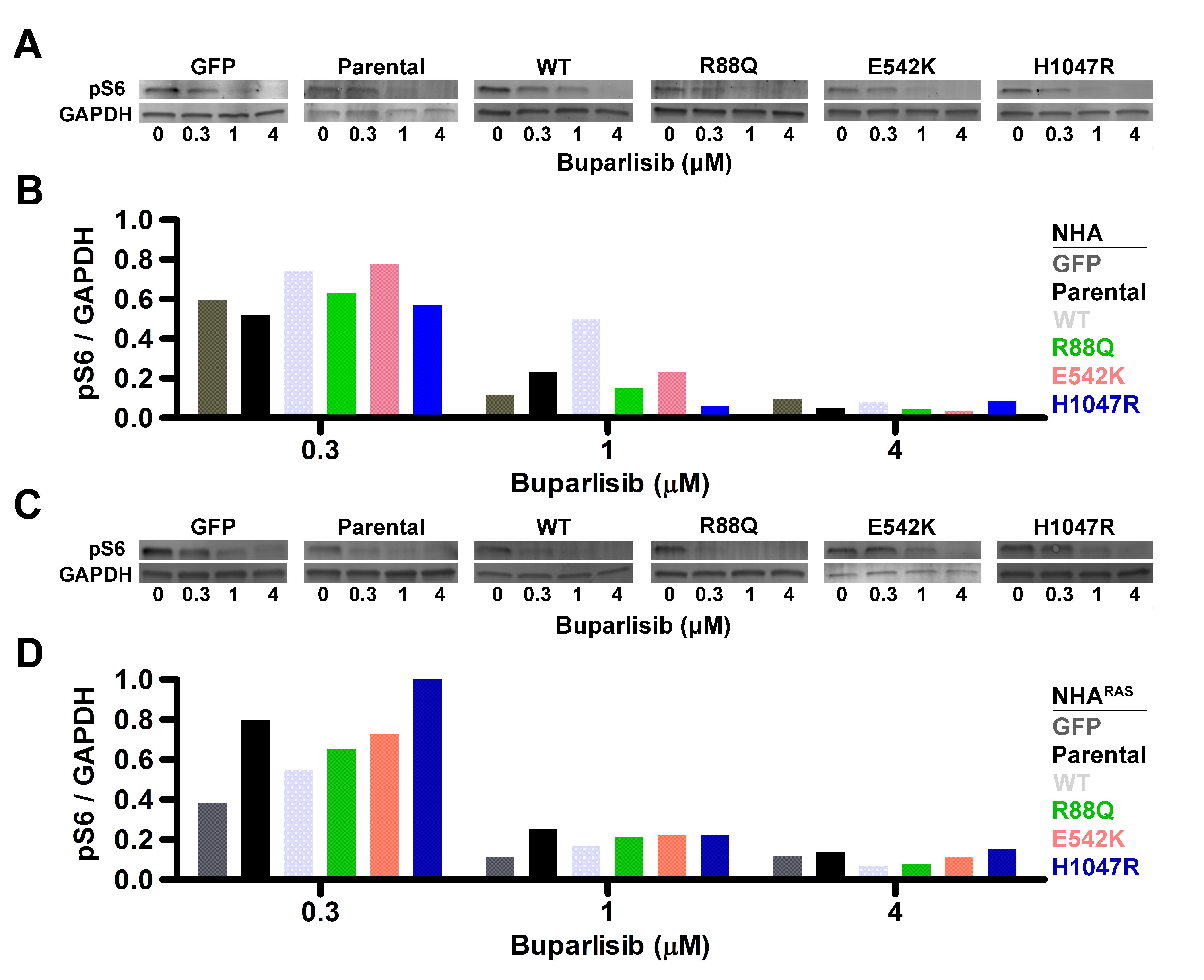

Supplement: S9 Fig — Representative immunoblots of control and PIK3CAmut NHA (A) and NHARAS (C) 24 h after buparlisib treatment. Immunoblot quantification demonstrated dose-dependent inhibition of distal PI3K in all NHA (B) and NHARAS (D) lines (N = 1–3 biologic replicates, Mean = 1.7). (TIF) [file pone.0200014.s009.tif]

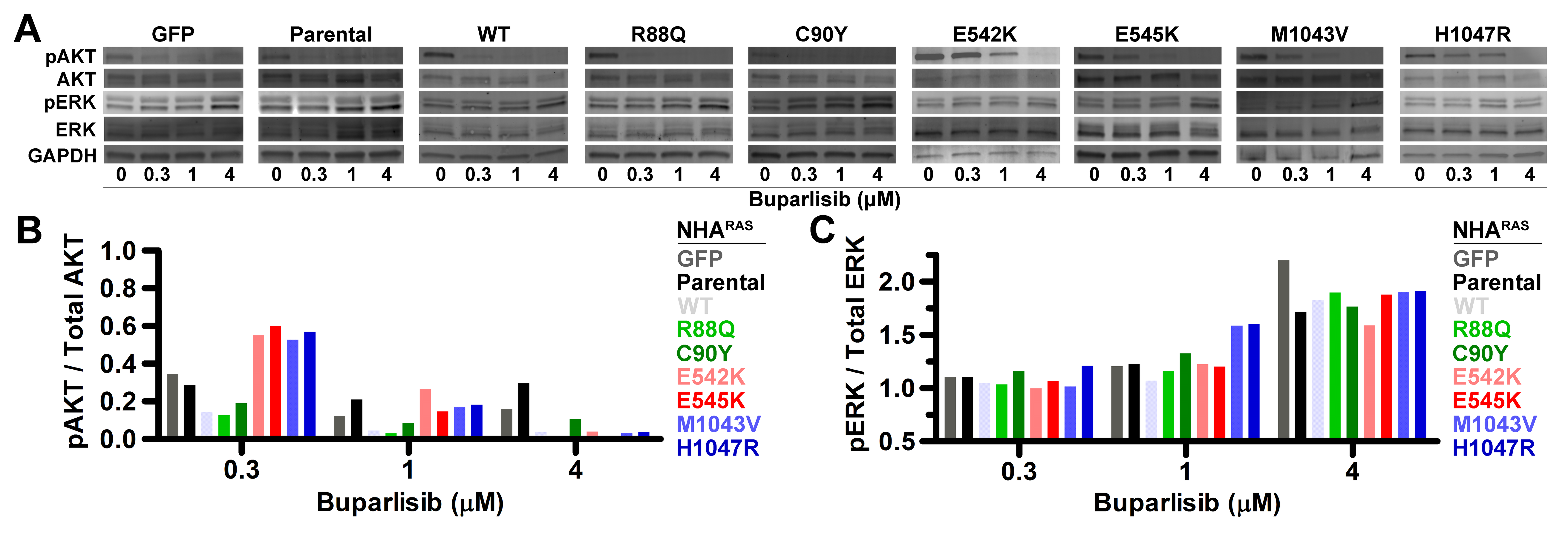

Supplement: S10 Fig — Representative immunoblots (A) and quantification of proximal PI3K (B) and MAPK (C) showed that within 24 h, buparlisib induced dose-dependent inhibition of PI3K signaling, with concurrent induction of MAPK in parental, GFP, PIK3CAWT, and all 6 PIK3CAmut NHARAS lines (N = 2–3 biologic replicates, Mean = 2.7). (TIF) [file pone.0200014.s010.tif]

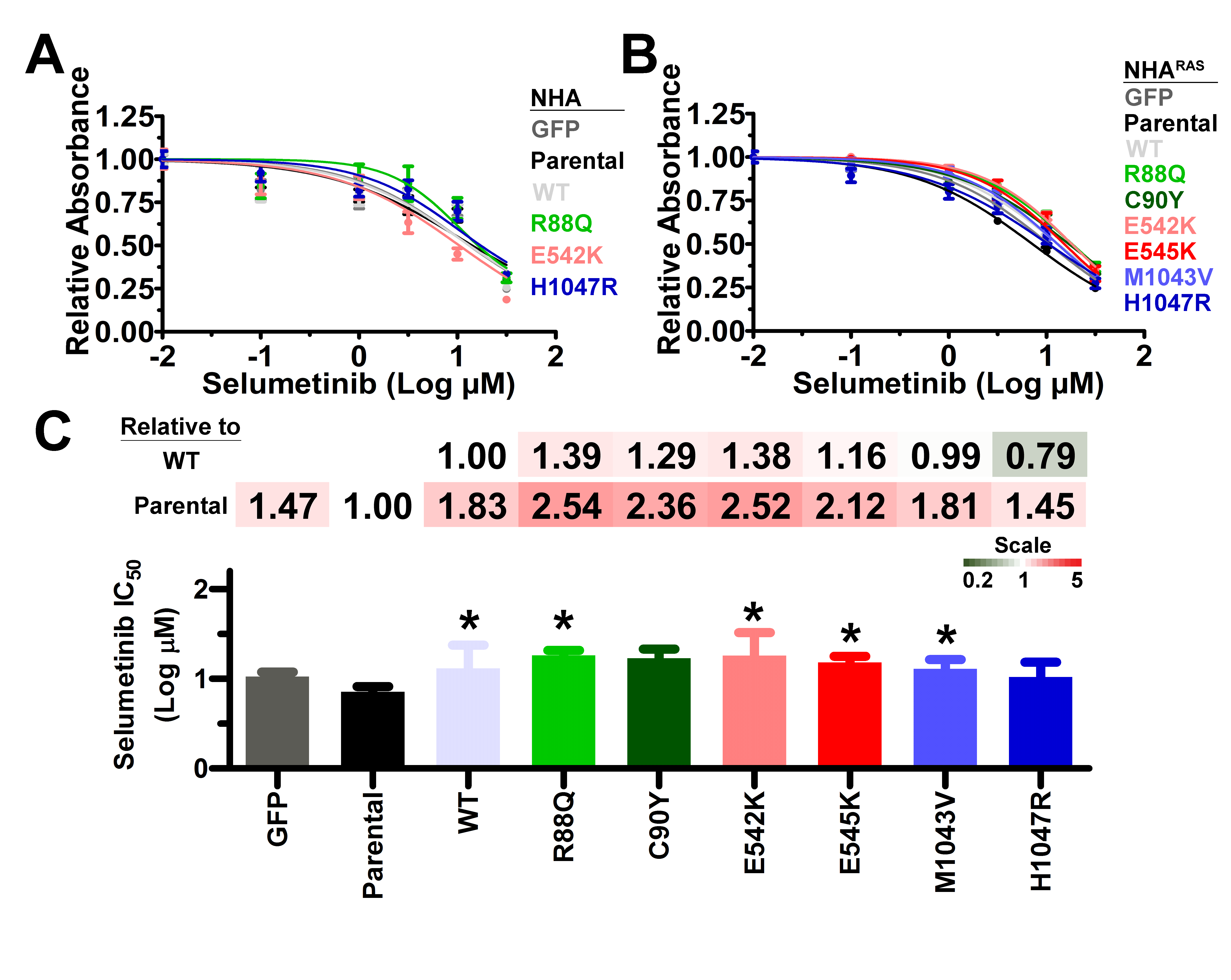

Supplement: S11 Fig — MTS assays showed that selumetinib caused dose-dependent decreases in growth of control and PIK3CAmut NHA (A) and NHARAS (B). Selumetinib IC50 were slightly increased by PIK3CAWT and all PIK3CAmut, except C90Y and H1047R, compared to parental NHARAS (*, P≤0.03) (C). (TIF) [file pone.0200014.s011.tif]

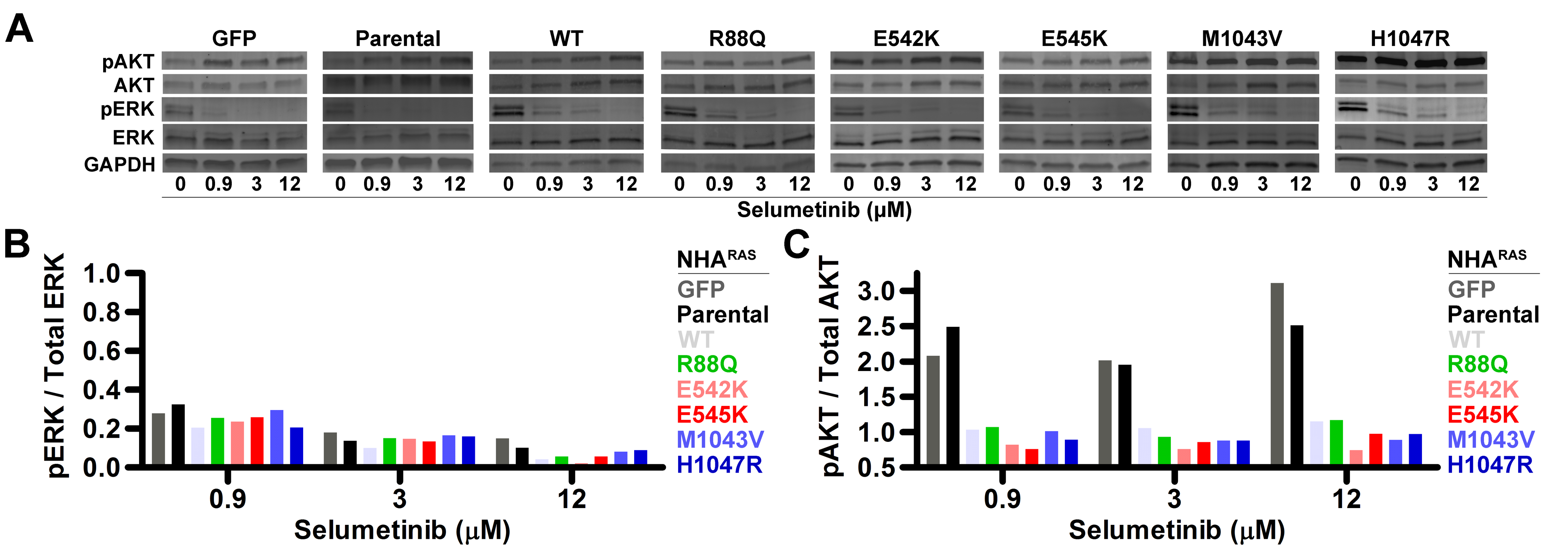

Supplement: S12 Fig — Representative immunoblots (A) and quantification of MAPK (B) and proximal PI3K (C) showed that selumetinib caused dose-dependent inhibition of MAPK regardless of PIKCAWT or PIK3CAmut status, but concurrent induction of proximal PI3K only occurred in parental and GFP NHARAS (N = 2–3 biologic replicates, Mean = 2.7). (TIF) [file pone.0200014.s012.tif]

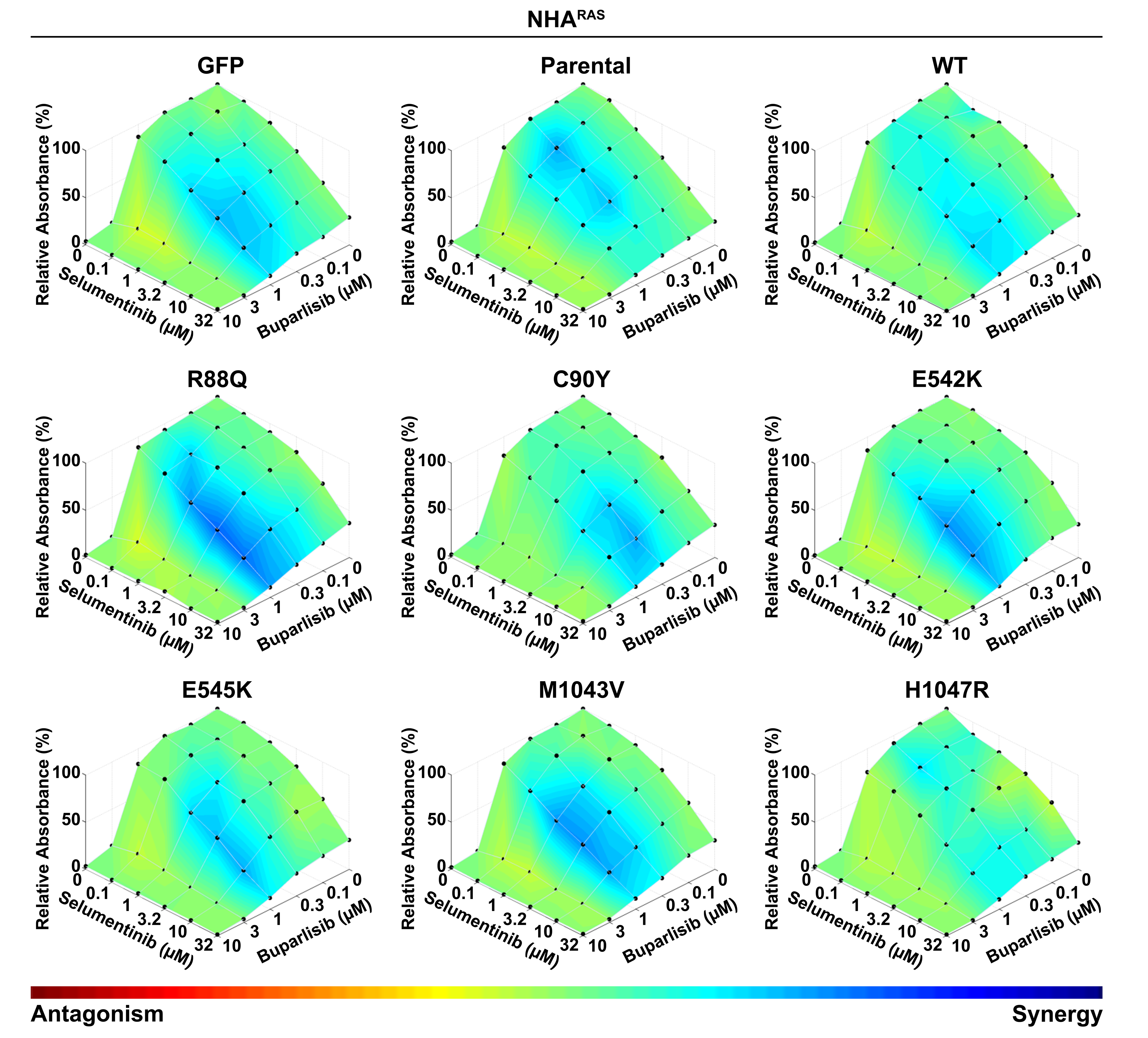

Supplement: S13 Fig — Buparlisib and selumetinib were synergistic in parental and PIK3CAmut NHARAS (Fig 6B) (TIF) [file pone.0200014.s013.tif]
