# Supplementary material for: PIK3CA missense mutations promote glioblastoma pathogenesis, but do not enhance targeted PI3K inhibition
Source: PLoS One. 2018 Jul 5;13(7):e0200014. doi: 10.1371/journal.pone.0200014 (PMC6033446; doi:10.1371/journal.pone.0200014)
Supplement: S14 Fig — (DOCX) [file pone.0200014.s014.docx]

***PIK3CA* missense mutations promote glioblastoma pathogenesis, but do not enhance PI3K inhibitor efficacy**

Robert S. McNeill, Emily E. Stroobant, Erin Smithberger, Demitra A. Canoutas, Madison K. Butler, Abigail K. Shelton, Shrey D. Patel, Juanita C. Limas, Kasey R. Skinner, Ryan E. Bash, Ralf S. Schmid, and C. Ryan Miller

**Supplemental Methods**

***PIK3CA* mutagenesis**

*PIK3CA^WT^* was excised from the pBabe backbone by sequential restriction digests with SalI and BamHI (New England BioLabs, Ipswich, MA). The pENTR4 vector was digested with BamHI and EcoRV, then *PIK3CA^WT^* was inserted by ligation. Alternatively, both pDEST-Flag-HA-GFP and pENTR4 vectors were digested with NcoI and EcoRI (New England BioLabs) followed by insertion of *GFP* into the pENTR4 vector by ligation. Six *PIK3CA* missense mutations (*PIK3CA^mut^*: R88Q, C90Y, E542K, E545K, M1043V, or H1047R) were generated by point mutagenesis of *PIK3CA^WT^* within the pENTR4 vector using the Q5 Site-Directed Mutagenesis Kit (New England Biolabs) according to manufacturer’s instructions. *GFP*, *PIK3CA^WT^*, and *PIK3CA^mut^* were transferred from their pENTR4 vectors to pLenti-PGK-Hygro-DEST vectors by recombination as previously described [1]. Subsequent point mutagenesis of *PIK3CA^WT^-* and *PIK3CA^mut^*-pLenti-PGK-Hygro-DEST vectors were performed using the Q5 Site-Directed Mutagenesis Kit to substitute an adenine to a guanine within an upstream ATG start codon in the NcoI restriction site from the pENTR4 vector. All point mutations were confirmed by Sanger sequencing (Genewiz, South Plainfield, NJ).

**Lentivirus production**

Lentiviral particles encoding *GFP*, *PIK3CA^WT^*, or individual *PIK3CA^mu^*^t^ were generated using 293FT cells (Invitrogen, Grand Island, NY) according to manufacturer’s instructions. Briefly, 5 x 10^5^ cells were plated on 10 cm plates in Dulbecco’s Modified Eagle’s Medium (DMEM) supplemented with 10% FBS, 1% penicillin/streptomycin, 2 mM GlutaMAX (Invitrogen), 0.1 mM MEM Non-Essential Amino Acids (Invitrogen), and 1 mM sodium pyruvate (Invitrogen) (293FT media). Medium was replaced the next day with fresh 293FT medium without penicillin/streptomycin and 9 μg *GFP*‑, *PIK3CA^wt^*-, or *PIK3CA^mut^*-pDEST, plus 9 μg pMDL/pRRE (Addgene, #12251, a gift from Didier Trono), 3.6 μg pRSV-Rev (Addgene, #12253, a gift from Didier Trono), and 1.89 μg pMD2.G (Addgene, #12259, a gift from Didier Trono) plasmids were transfected into these cells using Lipofectamine 2000 (Invitrogen) according to the manufacturer’s instructions [2]. Medium was exchanged with 293FT medium supplemented with 1 M HEPES (Invitrogen) after 24 hours. Supernatants containing lentiviruses were collected and fresh 293FT medium supplemented with 1M HEPES was added to cells 48 h and 72 h post-transfection. Viral supernatants from each time point were filtered through a 45 μm PES filter and stored at 4 ºC until all time points could be pooled, aliquoted, and stored at -80 ºC.

**Cell culture**

NHA and NHA^RAS^ lines were a kind gift from Dr. Russell O. Pieper [3]. All cells were maintained as adherent cultures at 37 ºC and 5% CO_2_ in DMEM supplemented with 5% FBS and 1% penicillin/streptomycin (complete DMEM). All *in vitro* experiments were performed in DMEM supplemented with 2.5% FBS and 1% penicillin/streptomycin (low-serum medium) unless otherwise stated. To create NHA and NHA^RAS^ lines expressing GFP, *PIK3CA^WT^*, or *PIK3CA^mut^*, 135,000 and 120,000 cells respectively were plated on 60 cm^2^ plates. Lentiviral vectors were added two days after plating and then incubated with cells overnight in complete DMEM containing 8 μg/ml polybrene (Sigma-Aldrich, St. Louis, MO) at 37ºC and 5% CO_2_. Two days post-infection, transduced cells were selected by culture in complete DMEM supplemented with 300 μg/ml hygromycin B (Gold Biotechnology, St. Louis, MO) for 14 days. Stable gene expression was confirmed by immunoblot for the HA tag on *PIK3CA^wt^* and *PIK3CA^mut^*.

**Drugs**

The PI3K inhibitor (PI3Ki) buparlisib (BKM120) and the MEK inhibitor (MEKi) selumetinib (AZD6244) were purchased from MedChem Express (Monmouth Junction, NJ) and Chemietek (Indianapolis, IN) and dissolved in dimethyl sulfoxide (DMSO).

**Immunoblots**

Control (parental, GFP, and *PIK3CA^WT^*) and *PIK3CA^mut^* NHA and NHA^RAS^ were plated on 60 or 100 cm^2^ plates. At ~60% confluence, cells were washed with Hank’s Balanced Salt Solution (HBSS), then serum-starved in DMEM supplemented with 0.5% FBS and 1% penicillin/streptomycin. Alternatively, cells were washed with HBSS, low-serum medium was added, then treated with vehicle control (DMSO), buparlisib, or selumetinib. Twenty-four h after serum starvation or drug treatment, cells were mechanically harvested and snap frozen. Cell pellets were lysed, and protein concentration was quantified using Pierce BCA Protein Assay Kit (Thermo Scientific, Waltham, MA) according to manufacturer’s instructions. Immunoblots were performed as previously described [4]. Briefly, protein samples (20 μg) were separated by gradient (4-15% or 8-16%) gel electrophoresis (SDS-PAGE, Bio-Rad, Hercules, CA) and transferred to PDVF membranes (EMD Millipore Corp, Billerica, MA). Membranes were probed with primary antibodies against GAPDH (EMD Millipore Corp, Billerica, MA, MAB374), AKT (#2920S), phosphorylated (p)AKT (Ser473, #4060), pS6 (Ser240/244, #2215), ERK1/2 (#4696), or phosphorylated (p)ERK1/2 (Thr202/Tyr204, #4370) all from Cell Signaling Technology (Danvers, MA). Blots were then probed with species-specific Alexa 488, 555, or 647 conjugated secondary antibodies (Life Technologies, Grand Island, NY) and imaged on a Typhoon Trio (GE Healthcare, Pittsburgh, PA). Alternatively, membranes were probed with an anti-HA-peroxidase antibody (Roche Applied Science, Penzberg, Germany, #12013819001) and imaged using enhanced chemiluminescence (Clarity Western ECL Substrate, Bio-Rad) on an Image Quant LAS 4000 (GE Healthcare). Band intensities were quantified using ImageJ (NIH, Bethesda, MD). Phospho-proteins were normalized to their respective total protein or GAPDH. Normalized band intensities from serum-starved immunoblots were set relative to an external standard and then normalized to parental and *PIK3CA^WT^* cells. Normalized band intensities from drug-treated samples were set relative to vehicle control cells. Differences between groups were determined by one-way ANOVA. Pairwise comparisons were performed by unpaired t-tests.

**Cell growth**

NHA and NHA^RAS^ lines were plated in triplicate or quadruplicate in 96-well plates at 1000 cells/well and 675 cells/well respectively. Changes in absorbance (cell growth) was assessed using CellTiter 96 Aqueous One Solution Cell Proliferation Assay (MTS, Promega, Madison, WI) according to manufacturer’s instructions. Briefly, absorbance at 490 nm was measured daily for 4-5 days using an Emax 96-well plate reader (Molecular Devices, Chicago, IL) equipped with SoftMax Pro 5 software. Baseline absorbance (low-serum media + MTS) was subtracted from absorbance of experimental samples, which were then set relative to day 0. Data (mean ± SEM) from 2-4 independent experiments (mean=3) were fit to an exponential growth equation and the rate constant k and doubling time [ln(2)/k] were calculated. Differences in growth rates (k) were compared using the extra-sum-of-squares F test.

**Cell migration**

Migration rate across a cell-free gap was determined using culture inserts (Ibidi, Munich, Germany) according to manufacturer’s instructions. Briefly, inserts were attached to individual wells of a 6-well plate. 26,000 cells were seeded into each well of the insert and allowed to adhere overnight. The following morning inserts were lifted to create a cell free gap that was imaged at 3 non-overlapping locations every 2 hours for 12 hours using a 4X objective on a VistaVision inverted microscope (Model #82026-630, VWR, Radnor, PA) equipped with a DV-300 digital camera. The area of the gap between the cell fronts was analyzed using ImageJ (NIH). Gap area was normalized to time 0 at each image location. Data from hours 2-12 were fit via linear regression and rates of gap closure were compared using analysis of covariance (ANCOVA).

**Colony formation in soft agar**

Colony formation was determined in 6-well treated plates as previously described with minor modifications [3, 5]. Briefly, a base layer of a low-serum DMEM/0.5% low melting temperature agarose (Denville Scientific INC., Holliston, MA) mixture was added to each well and allowed to solidify. Cells were harvested by trypsinization, placed in a mixture of low-serum DMEM/0.35% low melting temperature agarose, and plated (14,000 per well) in duplicate or triplicate. Cells were maintained at 37 ºC and 5% CO_2_ for 4 weeks, then fixed and stained with 0.005% crystal violet in 70% ethanol for ≥1 hr. Wells were washed with PBS, and colonies were imaged on a Typhoon Trio (GE Healthcare). Colonies ≥ 30 μm^2^ were automatically counted using ImageJ (NIH). Mean (± SEM) colonies per well was calculated from 2 independent experiments and data were compared using unpaired t-tests.

**Drug response**

Dose response assays were performed as previously described [4]. Briefly, cells were plated in triplicate in 96-well plates and maintained at 37˚C and 5% CO_2_. The following day they were treated with vehicle control (DMSO) or increasing concentrations of buparlisib and/or selumetinib. Effect~~s~~ of drugs on cell growth was determined with CellTiter 96 Aqueous One Solution Cell Proliferation Assay (MTS, Promega) according to manufacturer’s instructions. Briefly, absorbance at 490 nm was measured 5 days post-treatment as described above. Baseline absorbance was subtracted from experimental absorbance, which was then set relative to vehicle control. Data (mean ± SEM) were pooled from 2-4 (mean=3) independent experiments and fit to a non-linear, log [inhibitor] versus response curves with variable slopes, and IC_50_ were calculated. Differences in IC_50_ were compared using the extra-sum-of-squares F test. Synergism between MEKi and PI3Ki was determined by the BLISS method in Combenefit v1.31 [6].

**Cell cycle**

Control and *PIK3CA^mut^* NHA^RAS^ were plated in 12-well plates and treated with buparlisib or vehicle control the following day. Cell cycle analysis was performed 2 days post-treatment on a Guava EasyCyte Plus using Guava Cell Cycle Reagent according to the manufacturer’s instructions (EMD Millipore, Billerica, MA). Cell cycle distribution was determined using ModFit LT v3.2 (Verity, Topsham, ME). Percent cells in G_0_/G_1_, S, and G_2_/M were calculated from one experiment.

**Statistics**

Statistical analyses were performed in GraphPad Prism (La Jolla, CA) unless otherwise stated. P≤0.05 were considered significant. Error bars are SEM unless otherwise stated.

**References**

1. Campeau E, Ruhl VE, Rodier F, Smith CL, Rahmberg BL, Fuss JO, et al. (2009) A versatile viral system for expression and depletion of proteins in mammalian cells. PLoS One 4: e6529. doi: 10.1371/journal.pone.0006529. PMID: 19657394

2. Dull T, Zufferey R, Kelly M, Mandel RJ, Nguyen M, Trono D, et al. (1998) A third-generation lentivirus vector with a conditional packaging system. J Virol 72: 8463-8471. PMID: 9765382

3. Sonoda Y, Ozawa T, Hirose Y, Aldape KD, McMahon M, Berger MS, et al. (2001) Formation of intracranial tumors by genetically modified human astrocytes defines four pathways critical in the development of human anaplastic astrocytoma. Cancer Res 61: 4956-4960. PMID: 11431323

4. Schmid RS, Simon JM, Vitucci M, McNeill RS, Bash RE, Werneke AM, et al. (2016) Core pathway mutations induce de-differentiation of murine astrocytes into glioblastoma stem cells that are sensitive to radiation but resistant to temozolomide. Neuro Oncol 18: 962-973. doi: 10.1093/neuonc/nov321. PMID: 26826202

5. Borowicz S, Van Scoyk M, Avasarala S, Karuppusamy Rathinam MK, Tauler J, Bikkavilli RK, et al. (2014) The soft agar colony formation assay. J Vis Exp: e51998. doi: 10.3791/51998. PMID: 25408172

6. Di Veroli GY, Fornari C, Wang D, Mollard S, Bramhall JL, Richards FM, et al. (2016) Combenefit: an interactive platform for the analysis and visualization of drug combinations. Bioinformatics 32: 2866-2868. doi: 10.1093/bioinformatics/btw230. PMID: 27153664
