# Supplementary material for: PIK3CA missense mutations promote glioblastoma pathogenesis, but do not enhance targeted PI3K inhibition
Source: PLoS One. 2018 Jul 5;13(7):e0200014. doi: 10.1371/journal.pone.0200014 (PMC6033446; doi:10.1371/journal.pone.0200014)
Supplement: S15 Fig — (PPTX) [file pone.0200014.s015.pptx]

## Slide 1
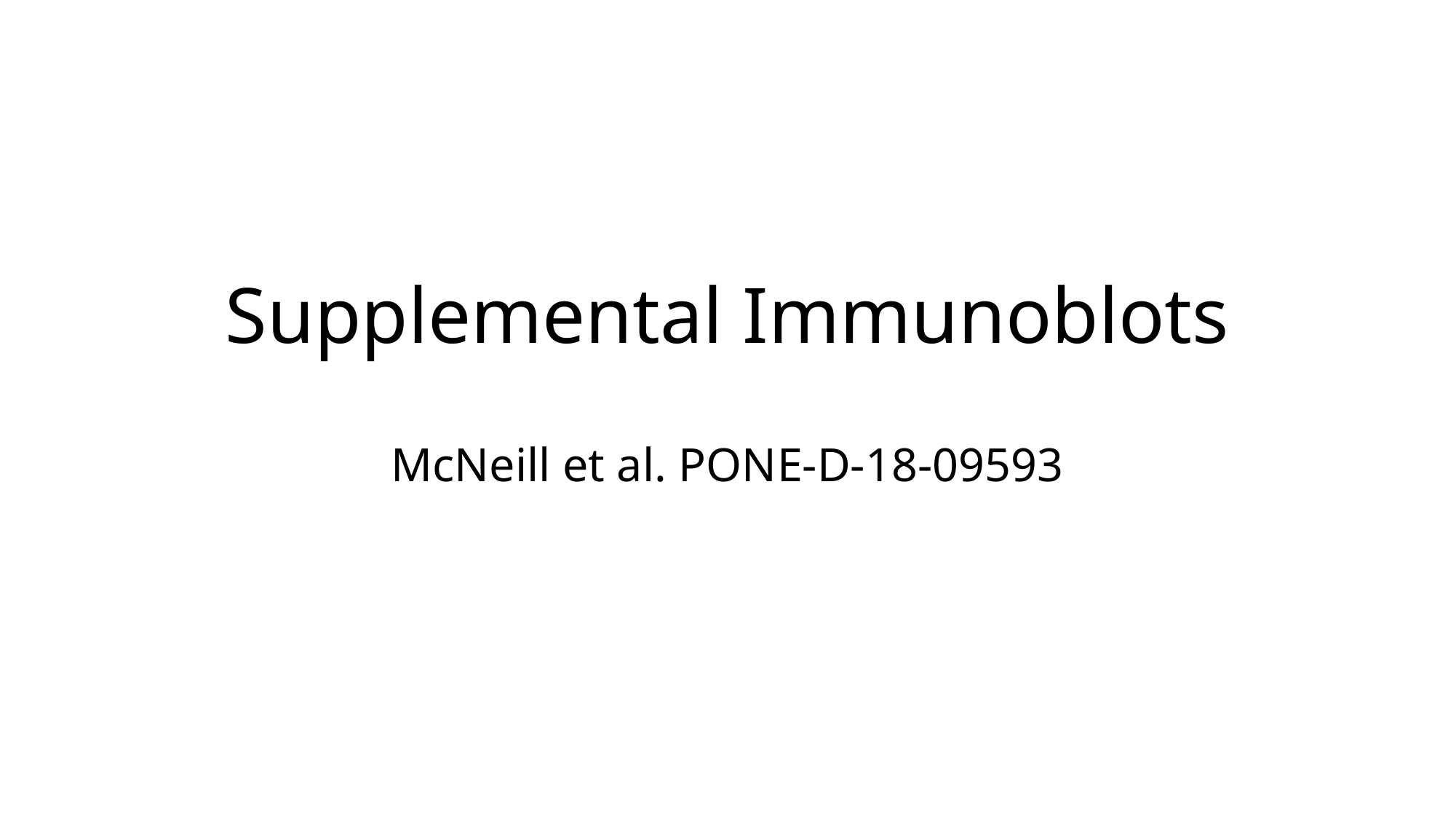

# Supplemental Immunoblots McNeill et al. PONE-D-18-09593

## Slide 2
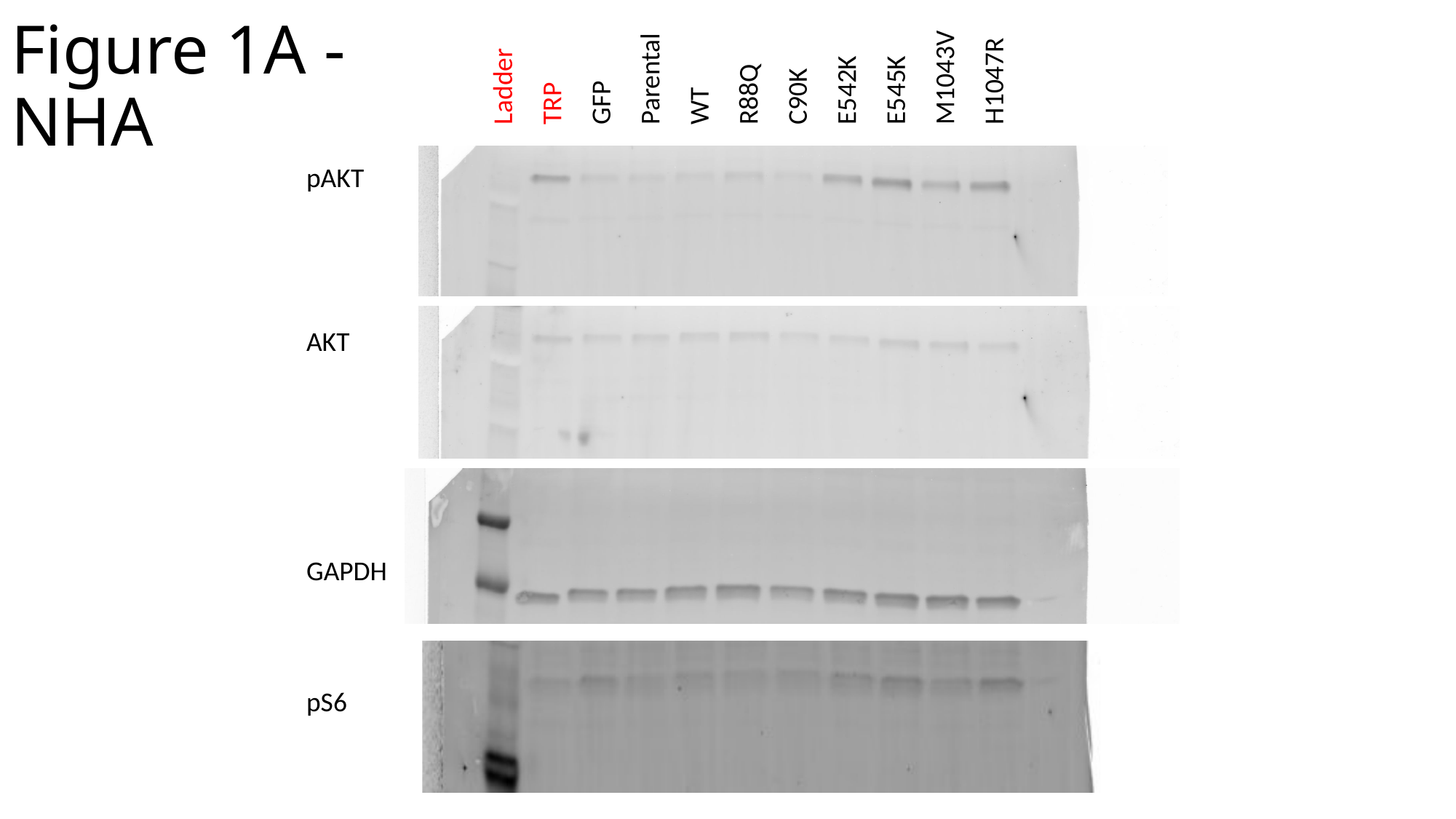

Ladder
TRP
GFP
Parental
WT
R88Q
C90K
E542K
E545K
M1043V
H1047R
# Figure 1A - NHA
pAKT
AKT
GAPDH
pS6

## Slide 3
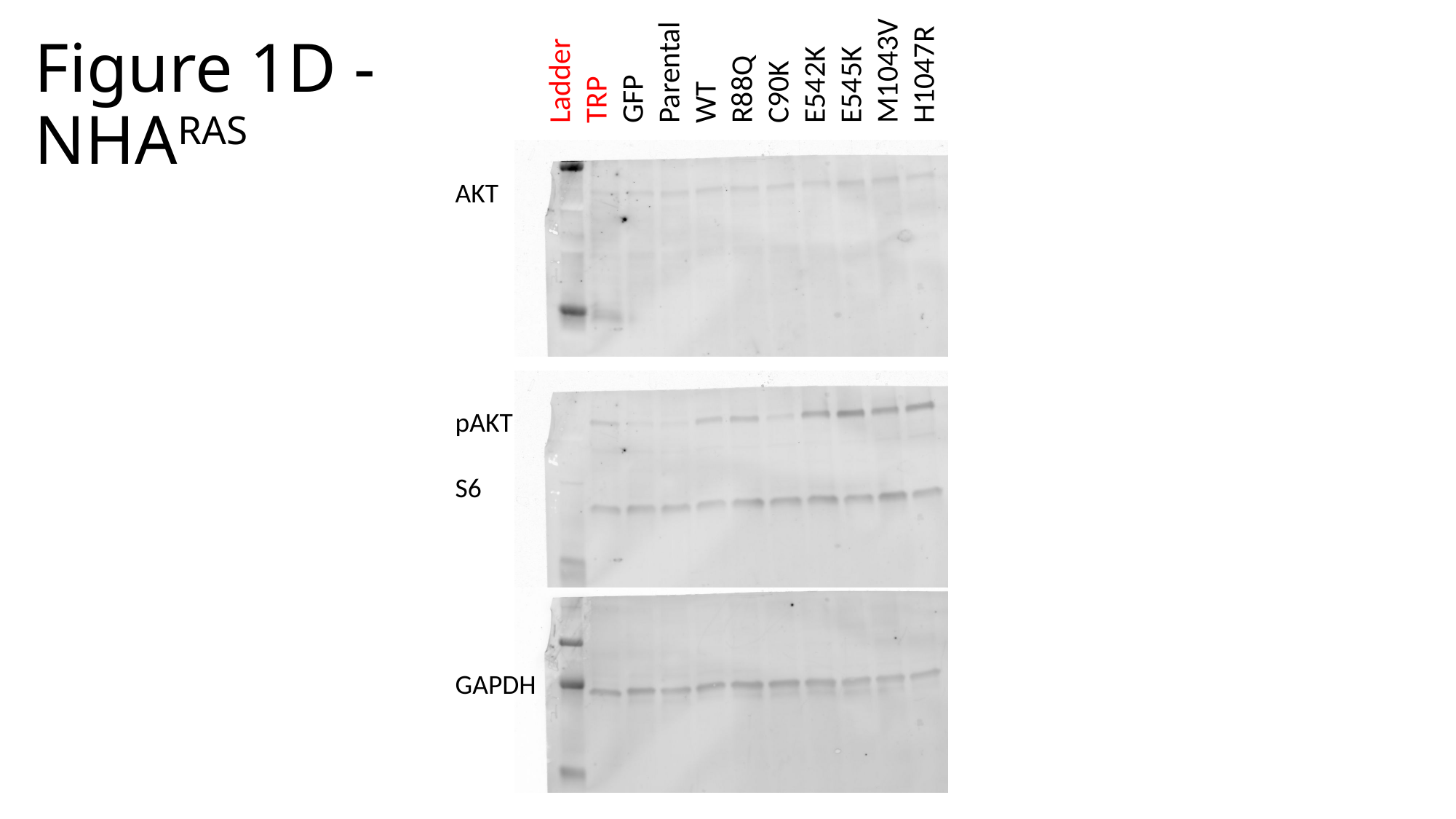

Ladder
TRP
GFP
Parental
WT
R88Q
C90K
E542K
E545K
M1043V
H1047R
# Figure 1D - NHARAS
AKT
pAKT
S6
GAPDH

## Slide 4
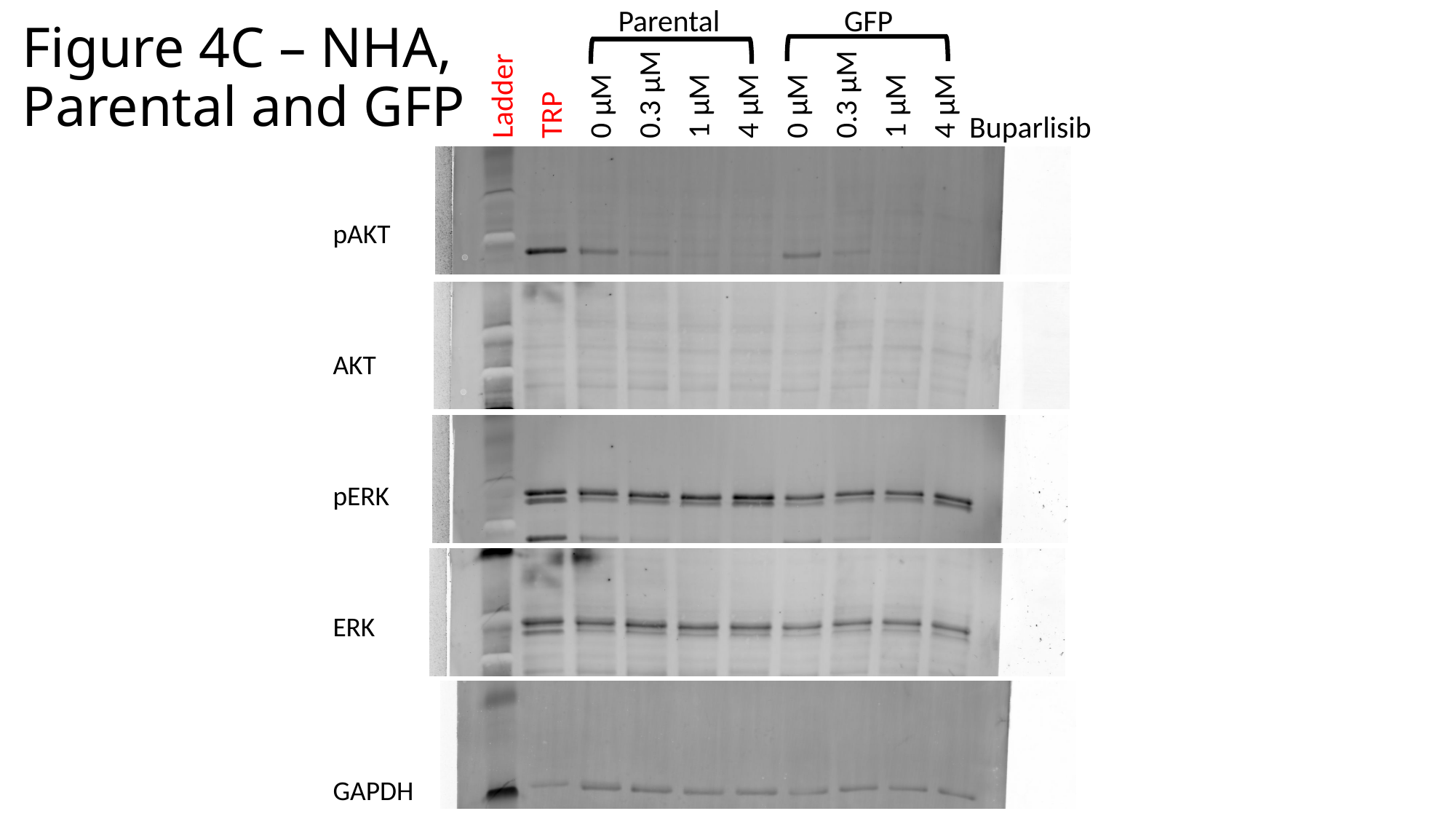

# Figure 4C – NHA, Parental and GFP
Parental GFP
Ladder
TRP
0 µM
0.3 µM
1 µM
4 µM
0 µM
0.3 µM
1 µM
4 µM
Buparlisib
pAKT
AKT
pERK
ERK
GAPDH

## Slide 5
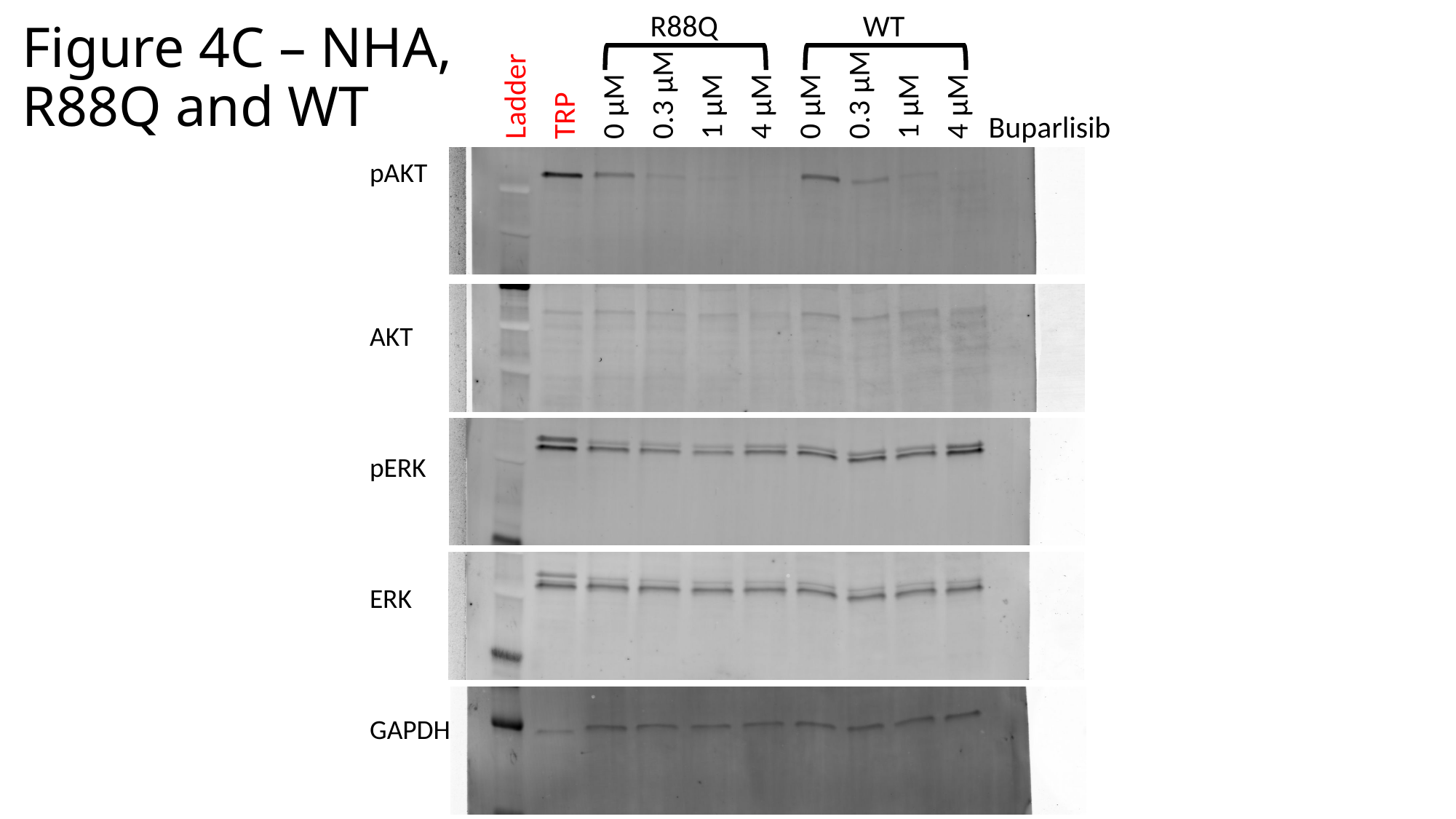

# Figure 4C – NHA, R88Q and WT
 R88Q WT
Ladder
TRP
0 µM
0.3 µM
1 µM
4 µM
0 µM
0.3 µM
1 µM
4 µM
Buparlisib
pAKT
AKT
pERK
ERK
GAPDH

## Slide 6
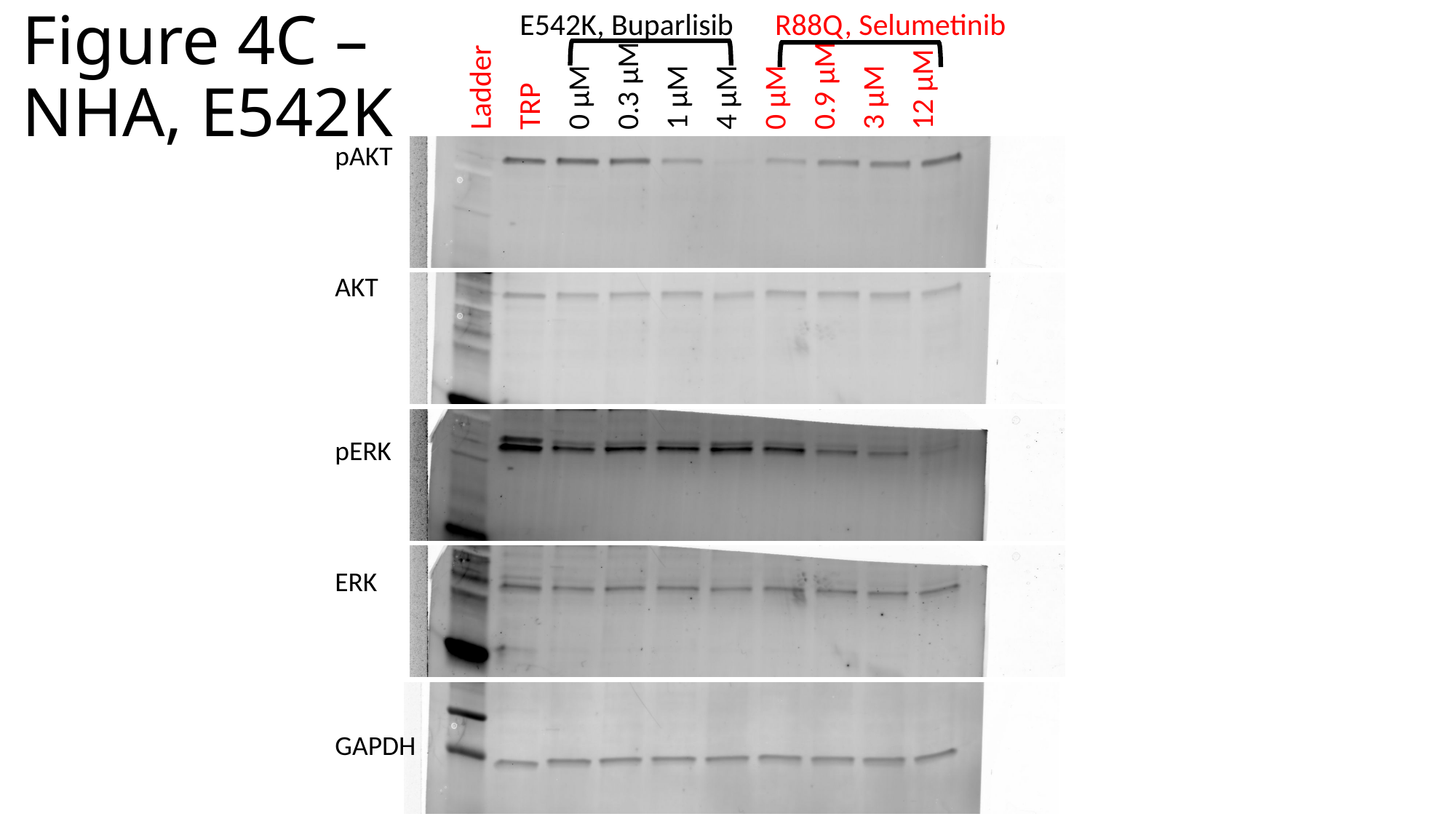

# Figure 4C – NHA, E542K
Ladder
TRP
0 µM
0.3 µM
1 µM
4 µM
0 µM
0.9 µM
3 µM
12 µM
 E542K, Buparlisib R88Q, Selumetinib
pAKT
AKT
pERK
ERK
GAPDH

## Slide 7
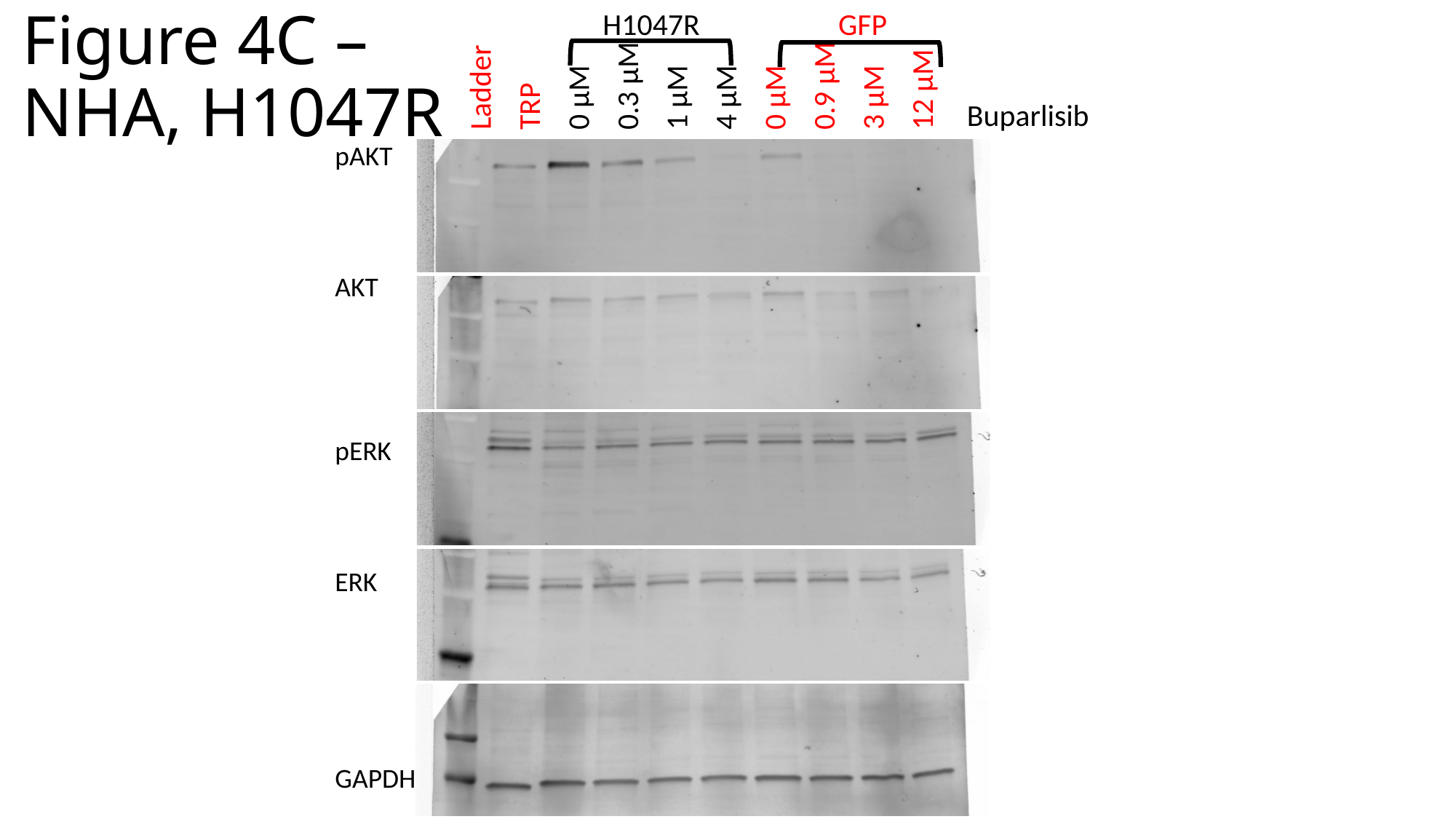

# Figure 4C – NHA, H1047R
Ladder
TRP
0 µM
0.3 µM
1 µM
4 µM
0 µM
0.9 µM
3 µM
12 µM
 H1047R GFP
Buparlisib
pAKT
AKT
pERK
ERK
GAPDH

## Slide 8
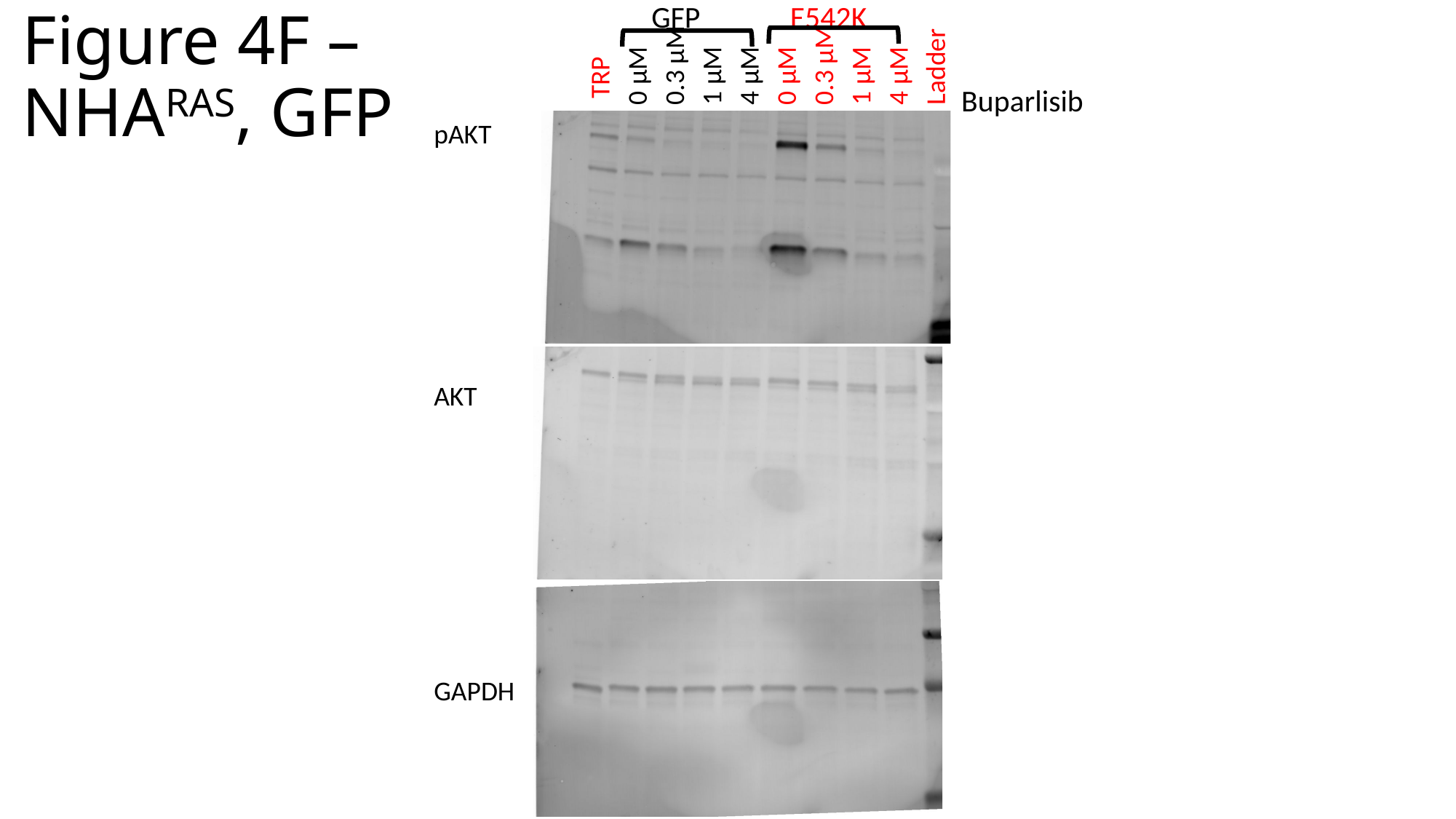

# Figure 4F – NHARAS, GFP
 TRP
0 µM
0.3 µM
1 µM
4 µM
0 µM
0.3 µM
1 µM
4 µM
Ladder
 GFP E542K
Buparlisib
pAKT
AKT
GAPDH

## Slide 9
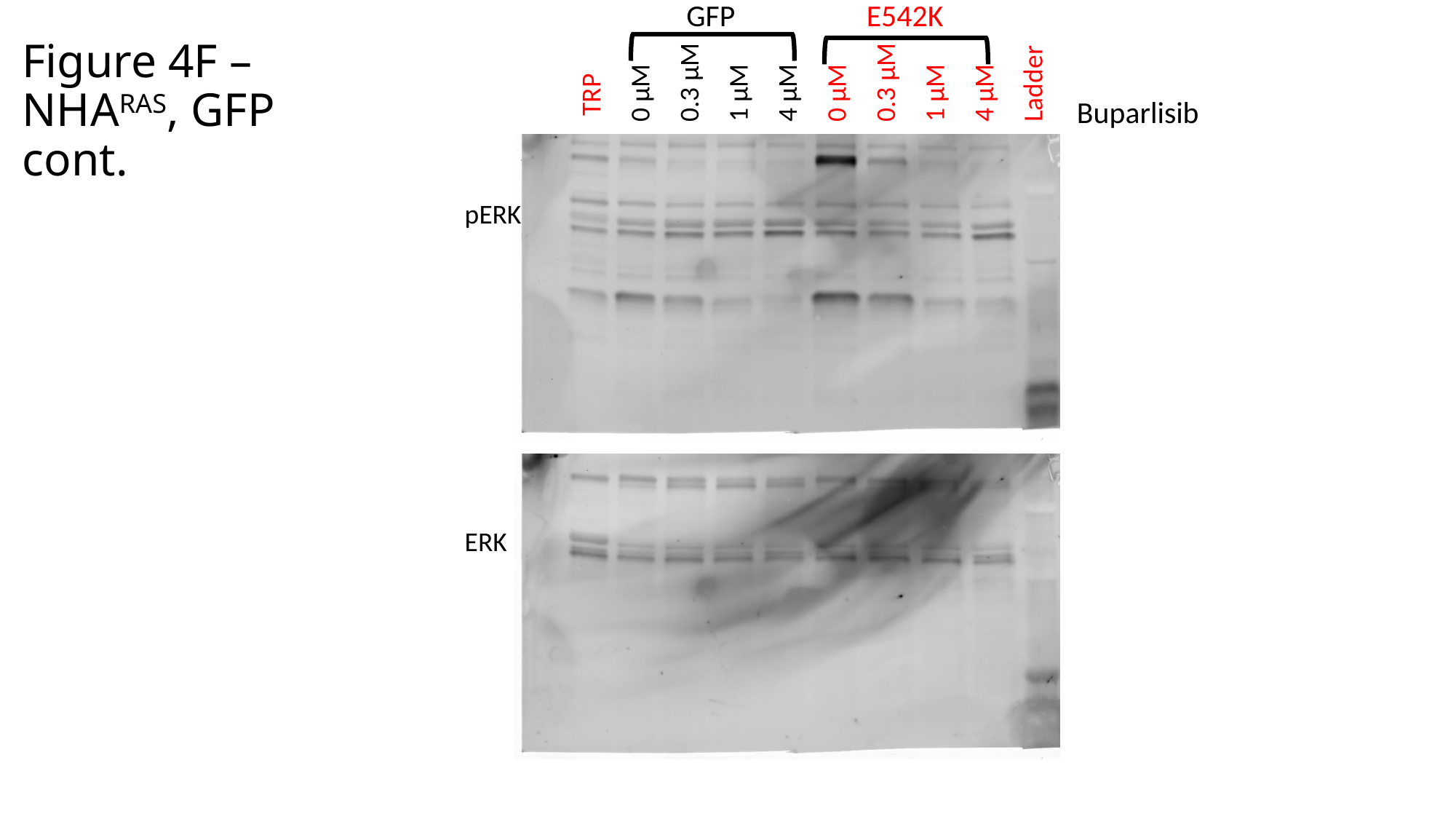

TRP
0 µM
0.3 µM
1 µM
4 µM
0 µM
0.3 µM
1 µM
4 µM
Ladder
 GFP E542K
# Figure 4F – NHARAS, GFP cont.
Buparlisib
pERK
ERK

## Slide 10
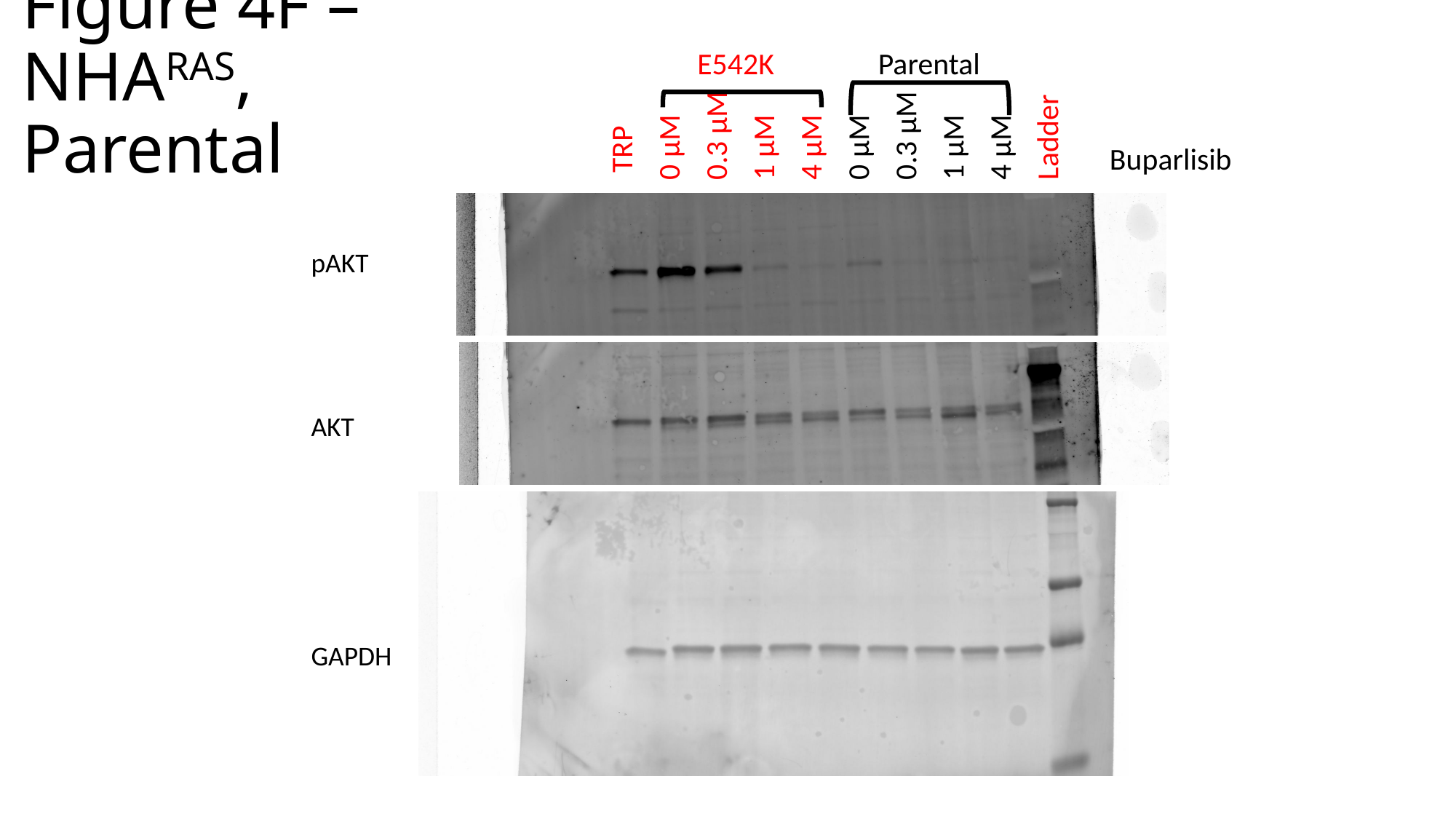

# Figure 4F – NHARAS, Parental
 E542K Parental
 TRP
0 µM
0.3 µM
1 µM
4 µM
0 µM
0.3 µM
1 µM
4 µM
Ladder
Buparlisib
pAKT
AKT
GAPDH

## Slide 11
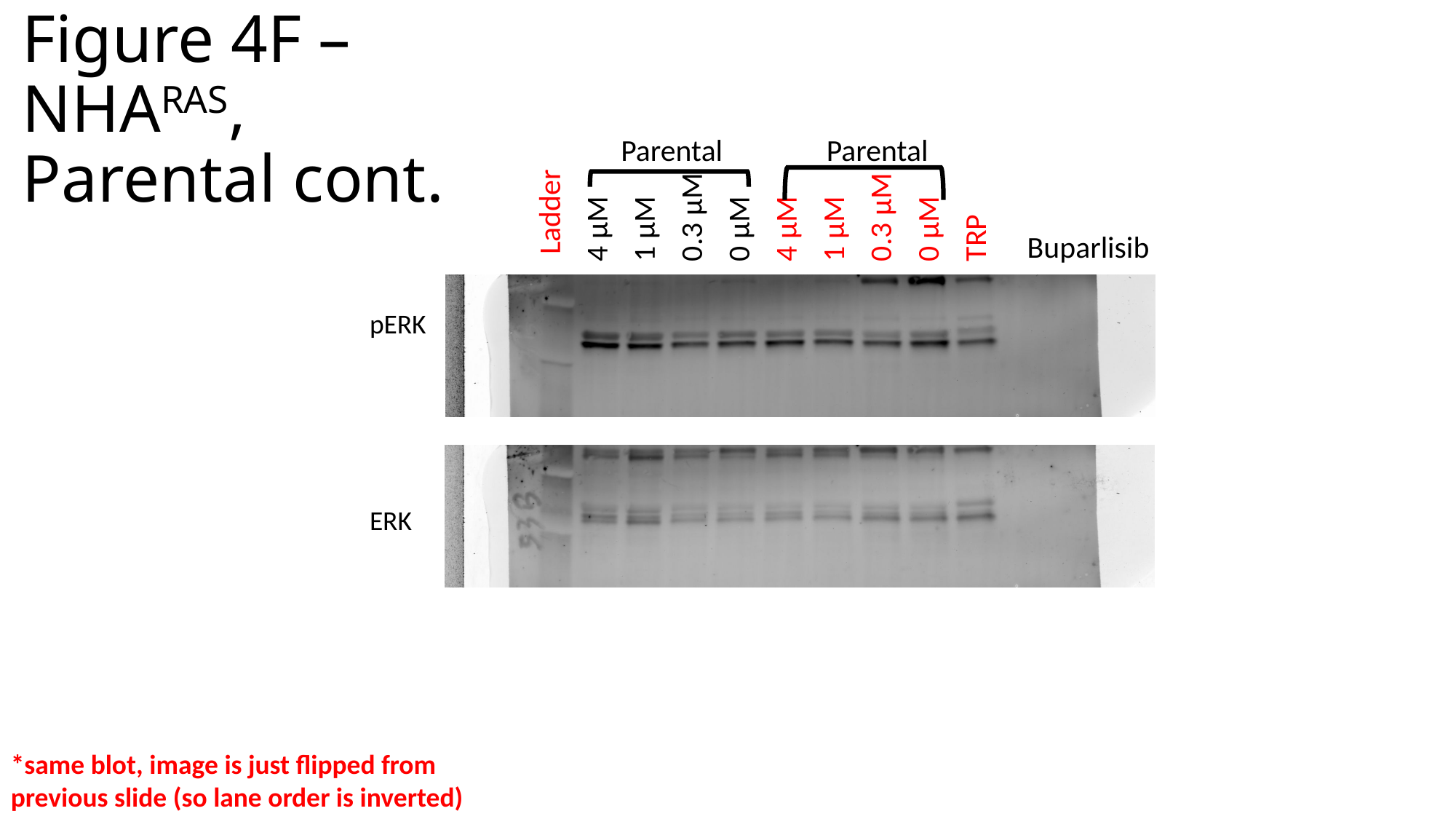

# Figure 4F – NHARAS, Parental cont.
 Parental Parental
 Ladder
4 µM
1 µM
0.3 µM
0 µM
4 µM
1 µM
0.3 µM
0 µM
TRP
Buparlisib
pERK
ERK
*same blot, image is just flipped from previous slide (so lane order is inverted)

## Slide 12
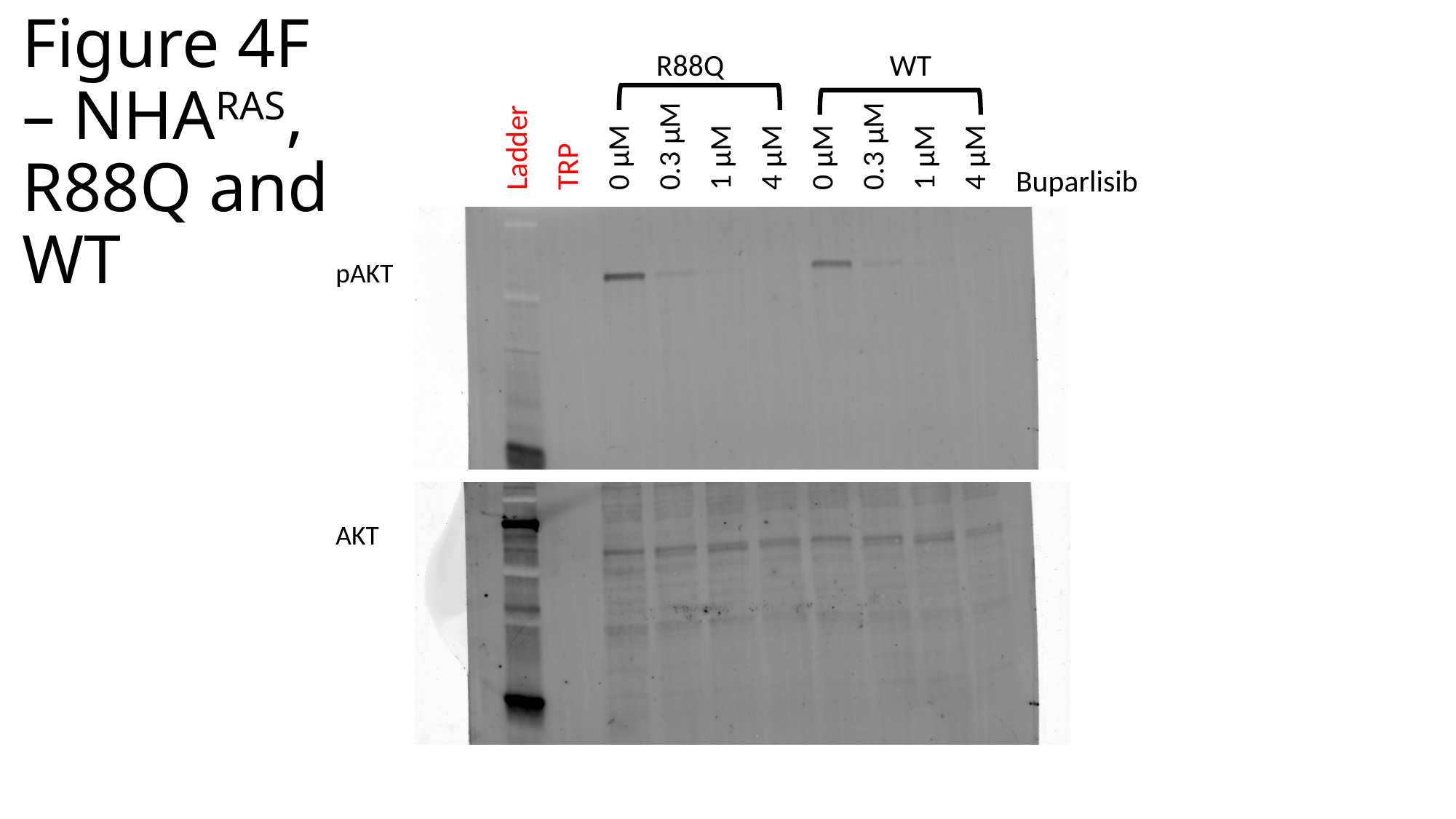

# Figure 4F – NHARAS, R88Q and WT
 R88Q WT
Ladder
TRP
0 µM
0.3 µM
1 µM
4 µM
0 µM
0.3 µM
1 µM
4 µM
Buparlisib
pAKT
AKT

## Slide 13
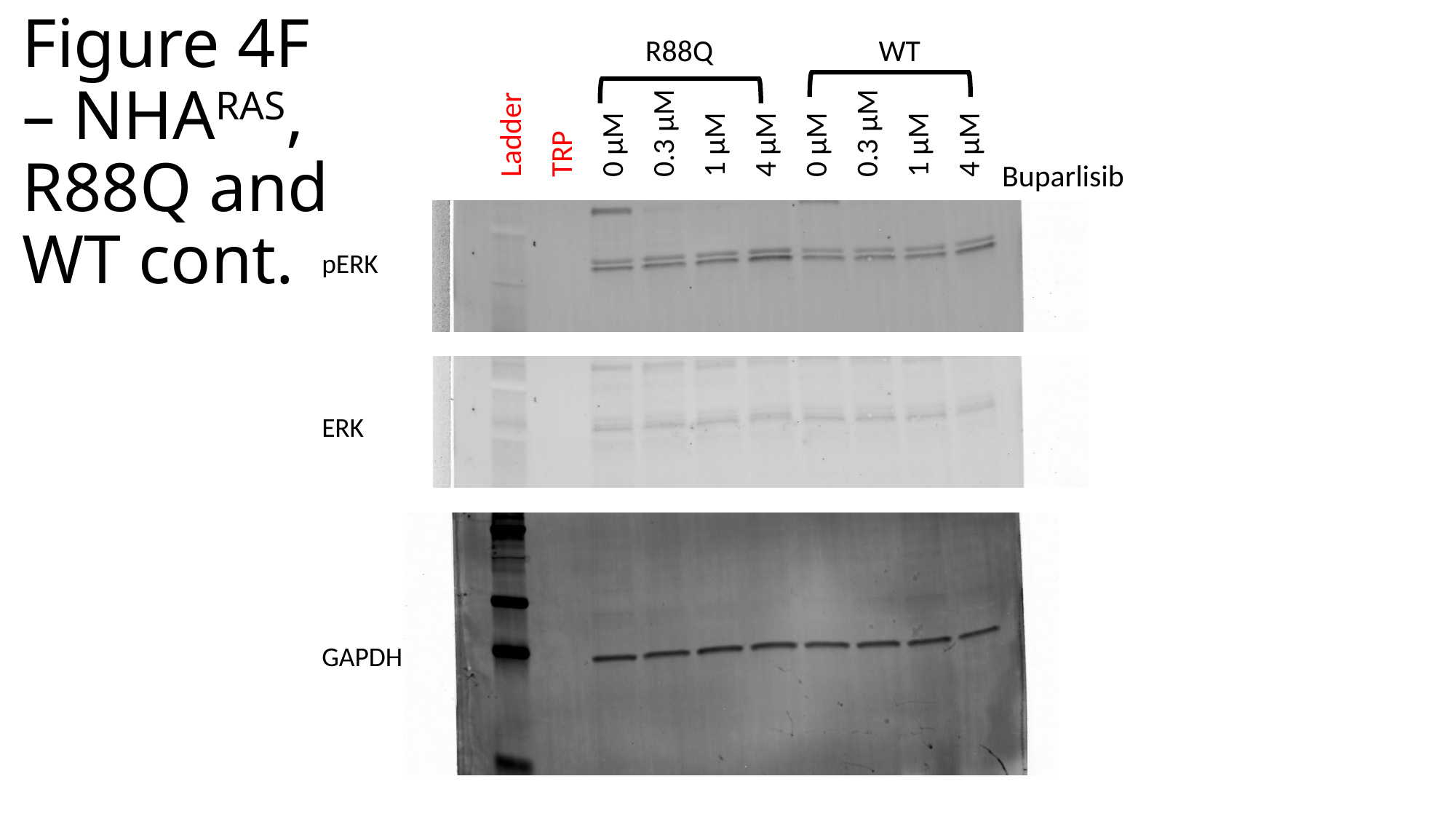

# Figure 4F – NHARAS, R88Q and WT cont.
 R88Q WT
Ladder
TRP
0 µM
0.3 µM
1 µM
4 µM
0 µM
0.3 µM
1 µM
4 µM
Buparlisib
pERK
ERK
GAPDH

## Slide 14
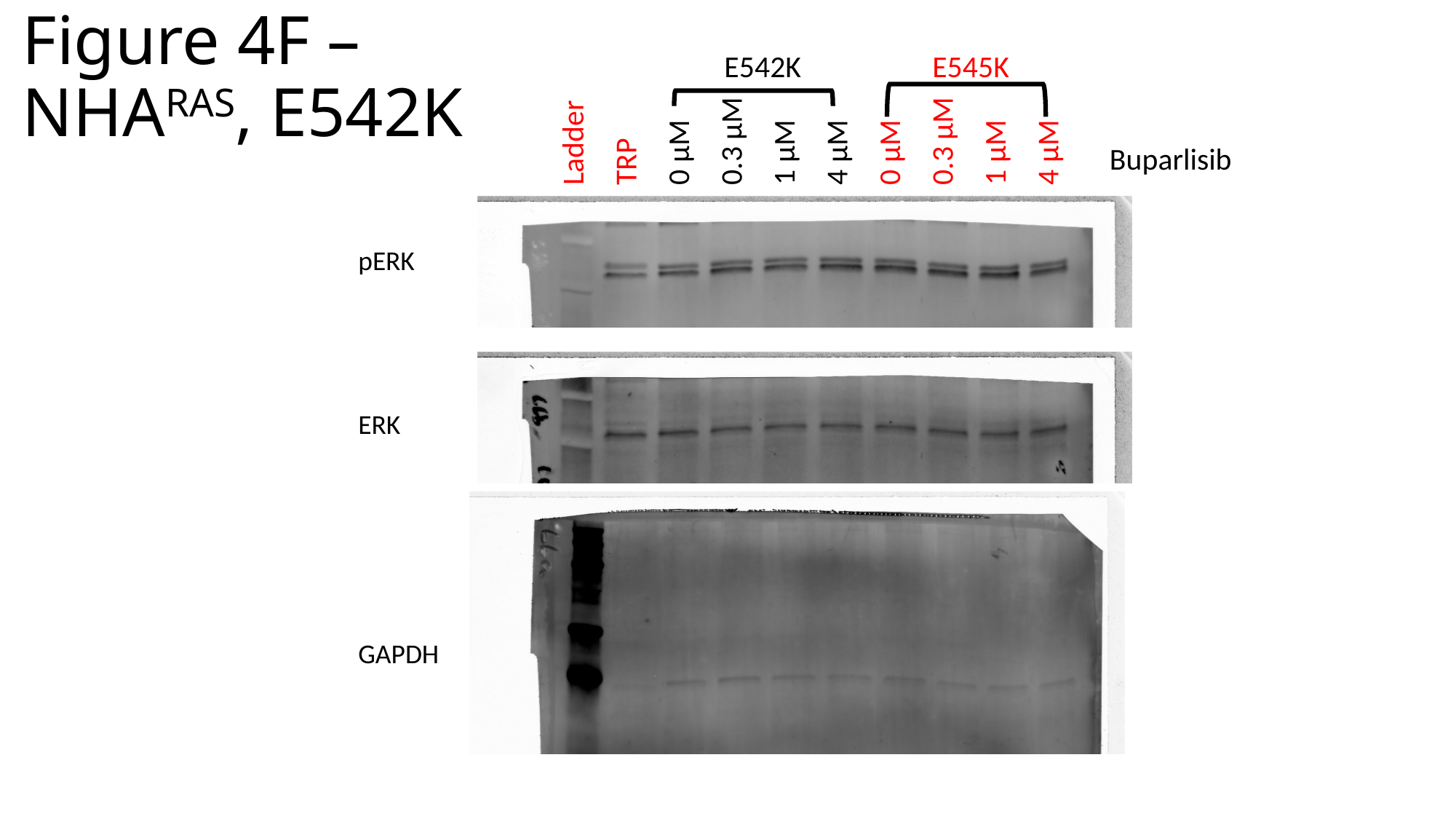

# Figure 4F – NHARAS, E542K
 E542K E545K
Ladder
TRP
0 µM
0.3 µM
1 µM
4 µM
0 µM
0.3 µM
1 µM
4 µM
Buparlisib
pERK
ERK
GAPDH

## Slide 15
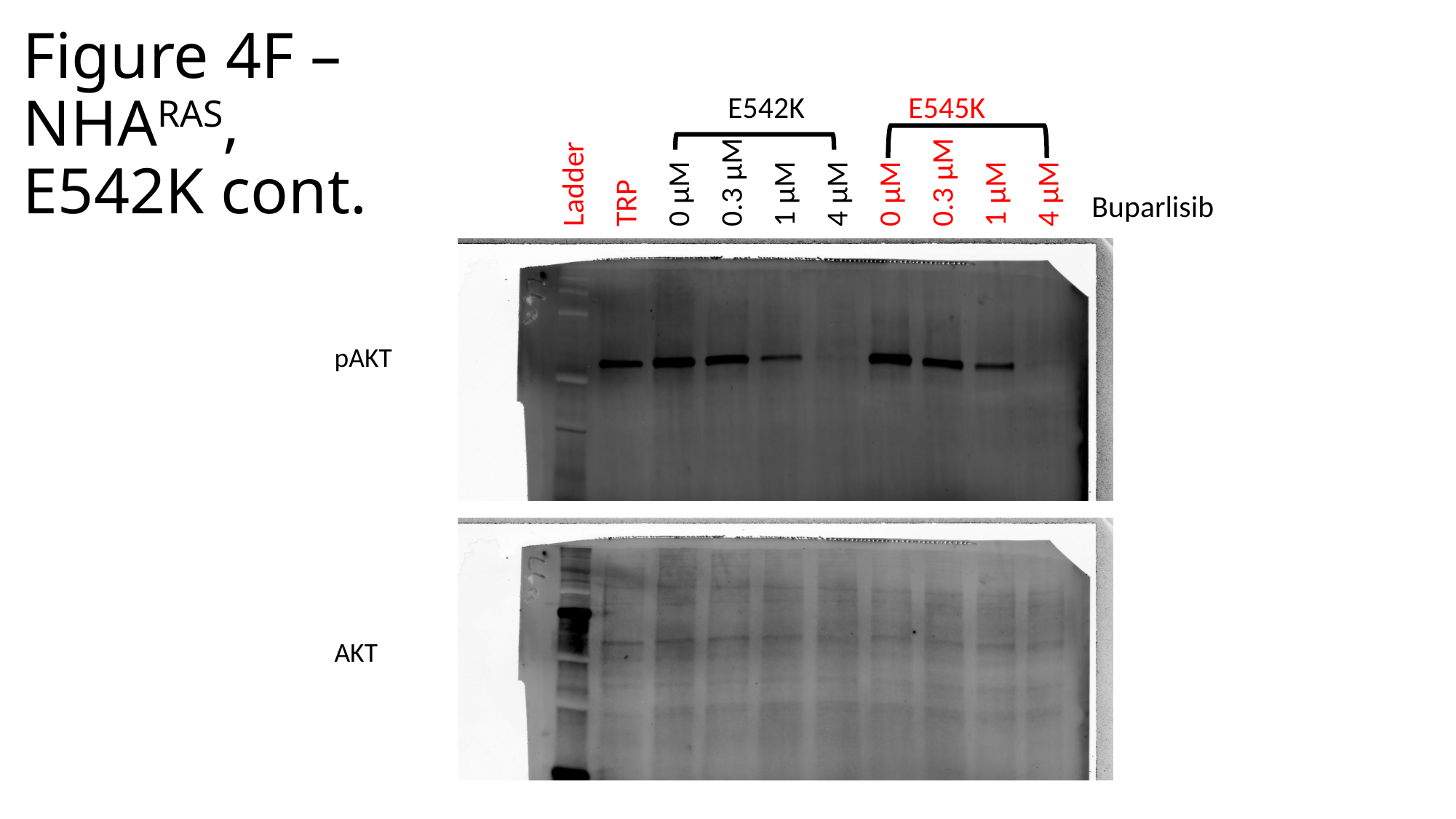

# Figure 4F – NHARAS, E542K cont.
 E542K E545K
Ladder
TRP
0 µM
0.3 µM
1 µM
4 µM
0 µM
0.3 µM
1 µM
4 µM
Buparlisib
pAKT
AKT

## Slide 16
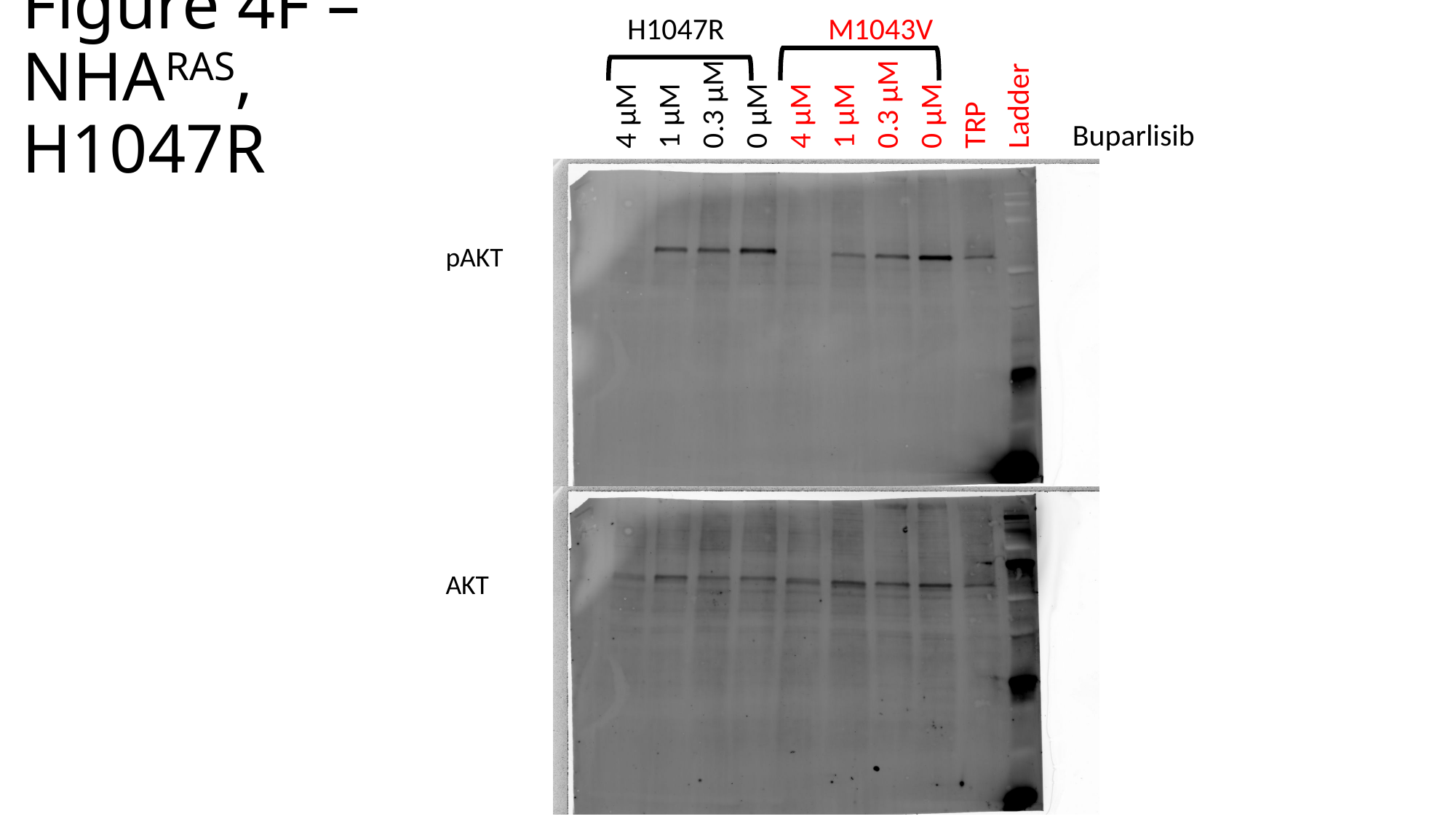

# Figure 4F – NHARAS, H1047R
 H1047R M1043V
4 µM
1 µM
0.3 µM
0 µM
4 µM
1 µM
0.3 µM
0 µM
TRP
Ladder
Buparlisib
pAKT
AKT

## Slide 17
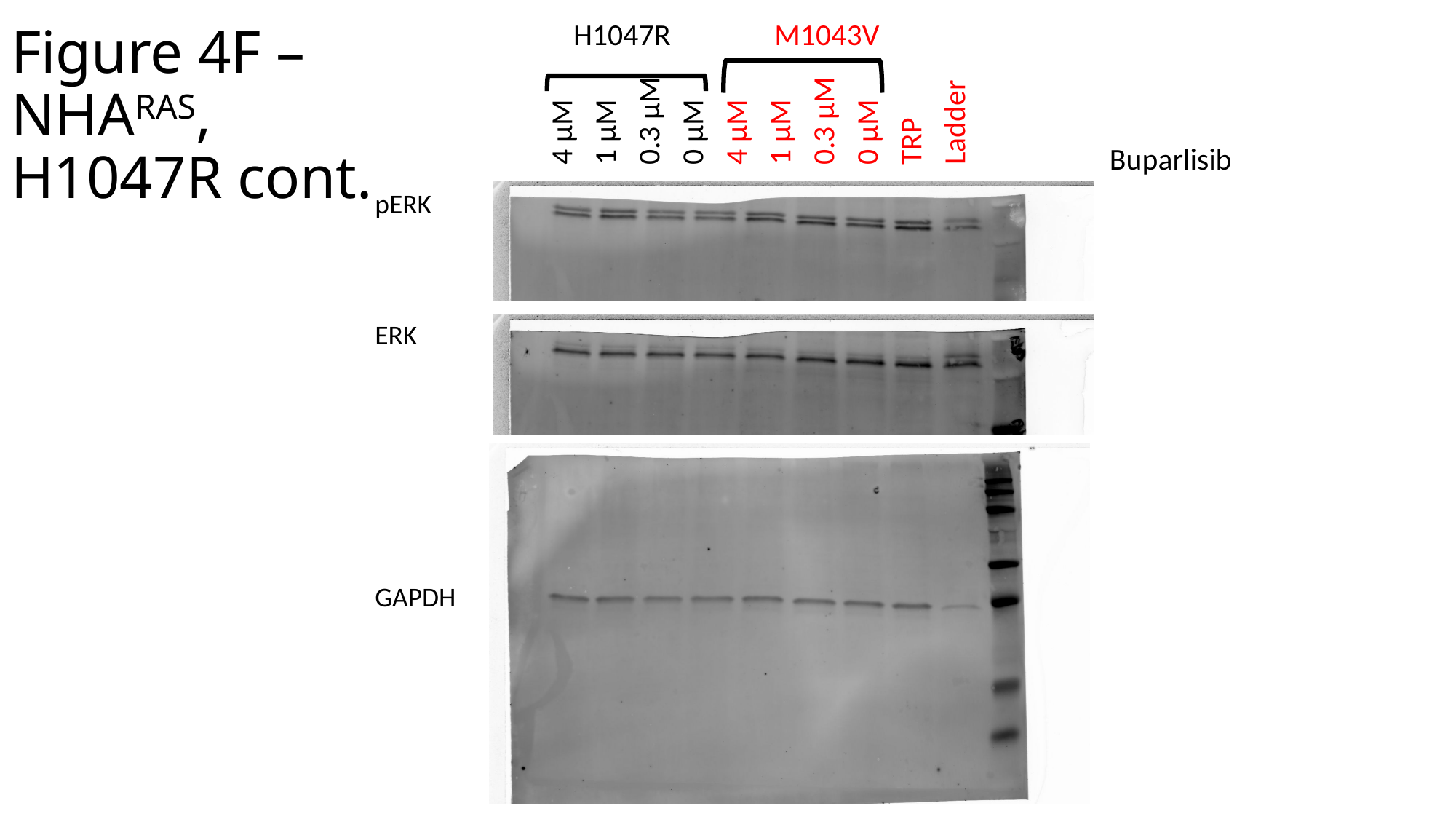

# Figure 4F – NHARAS, H1047R cont.
 H1047R M1043V
4 µM
1 µM
0.3 µM
0 µM
4 µM
1 µM
0.3 µM
0 µM
TRP
Ladder
Buparlisib
pERK
ERK
GAPDH

## Slide 18
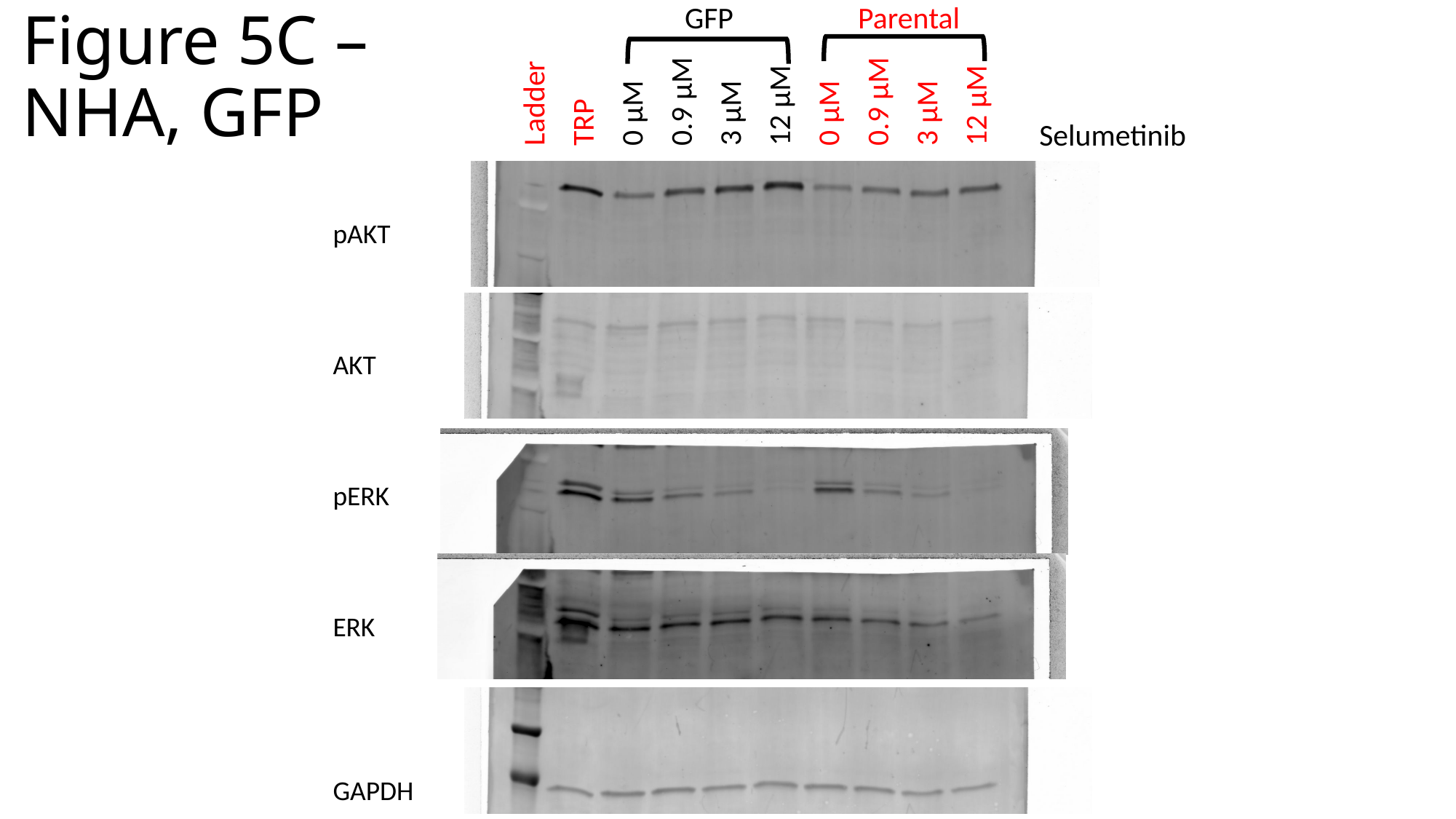

# Figure 5C – NHA, GFP
GFP Parental
Ladder
TRP
0 µM
0.9 µM
3 µM
12 µM
0 µM
0.9 µM
3 µM
12 µM
Selumetinib
pAKT
AKT
pERK
ERK
GAPDH

## Slide 19
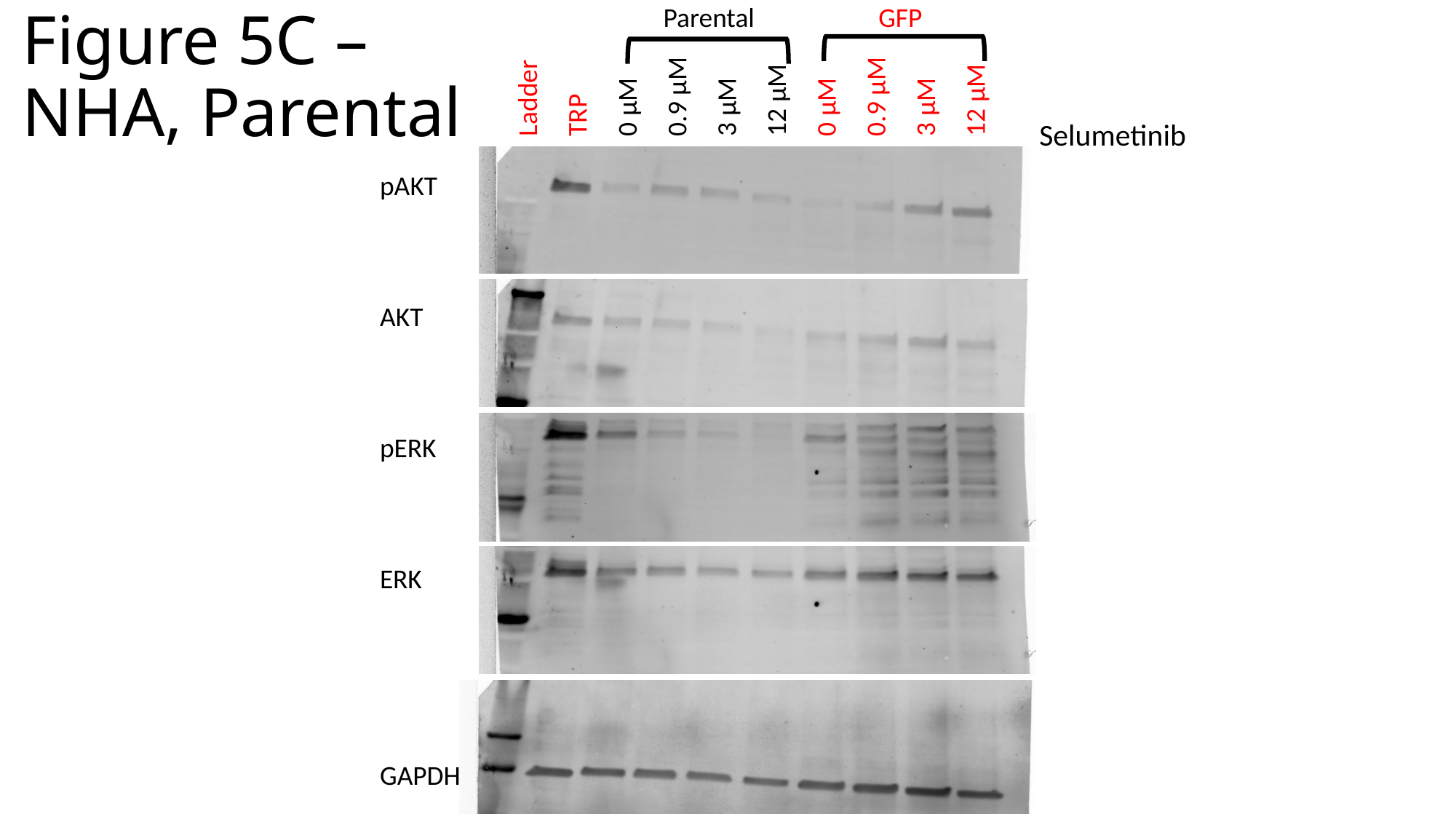

# Figure 5C – NHA, Parental
Parental GFP
Ladder
TRP
0 µM
0.9 µM
3 µM
12 µM
0 µM
0.9 µM
3 µM
12 µM
Selumetinib
pAKT
AKT
pERK
ERK
GAPDH

## Slide 20
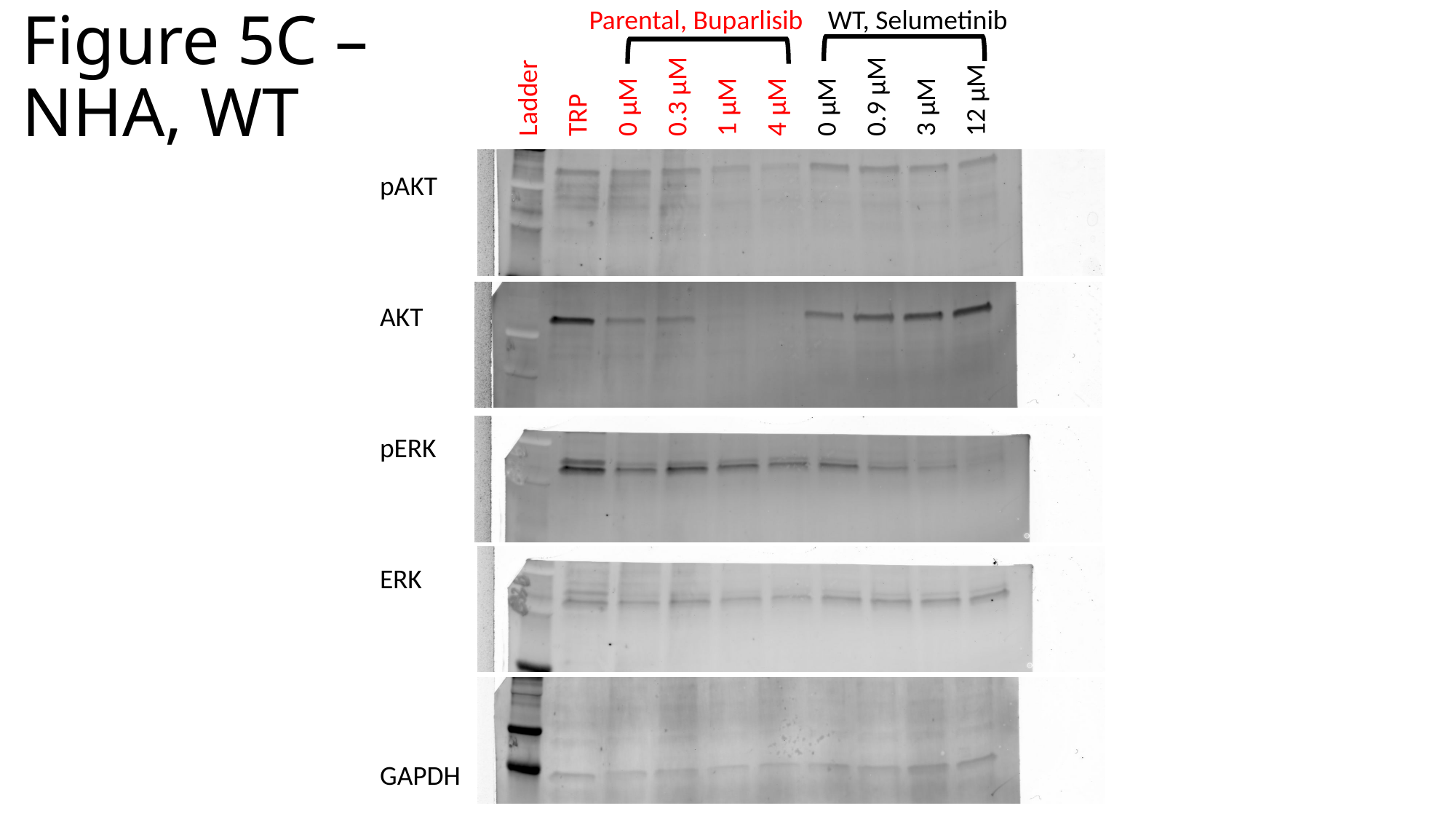

# Figure 5C – NHA, WT
Parental, Buparlisib WT, Selumetinib
Ladder
TRP
0 µM
0.3 µM
1 µM
4 µM
0 µM
0.9 µM
3 µM
12 µM
pAKT
AKT
pERK
ERK
GAPDH

## Slide 21
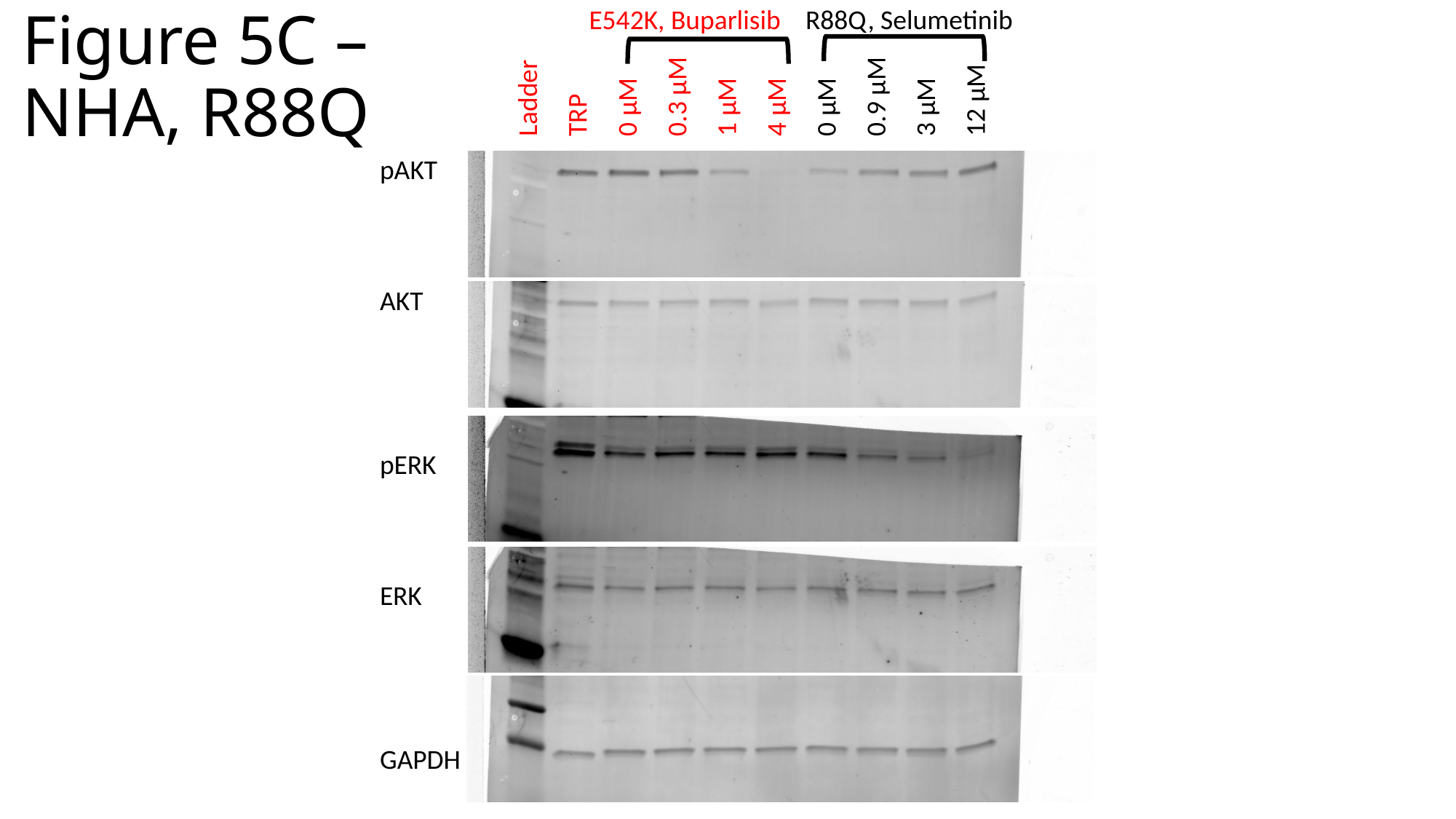

# Figure 5C – NHA, R88Q
E542K, Buparlisib R88Q, Selumetinib
Ladder
TRP
0 µM
0.3 µM
1 µM
4 µM
0 µM
0.9 µM
3 µM
12 µM
pAKT
AKT
pERK
ERK
GAPDH

## Slide 22
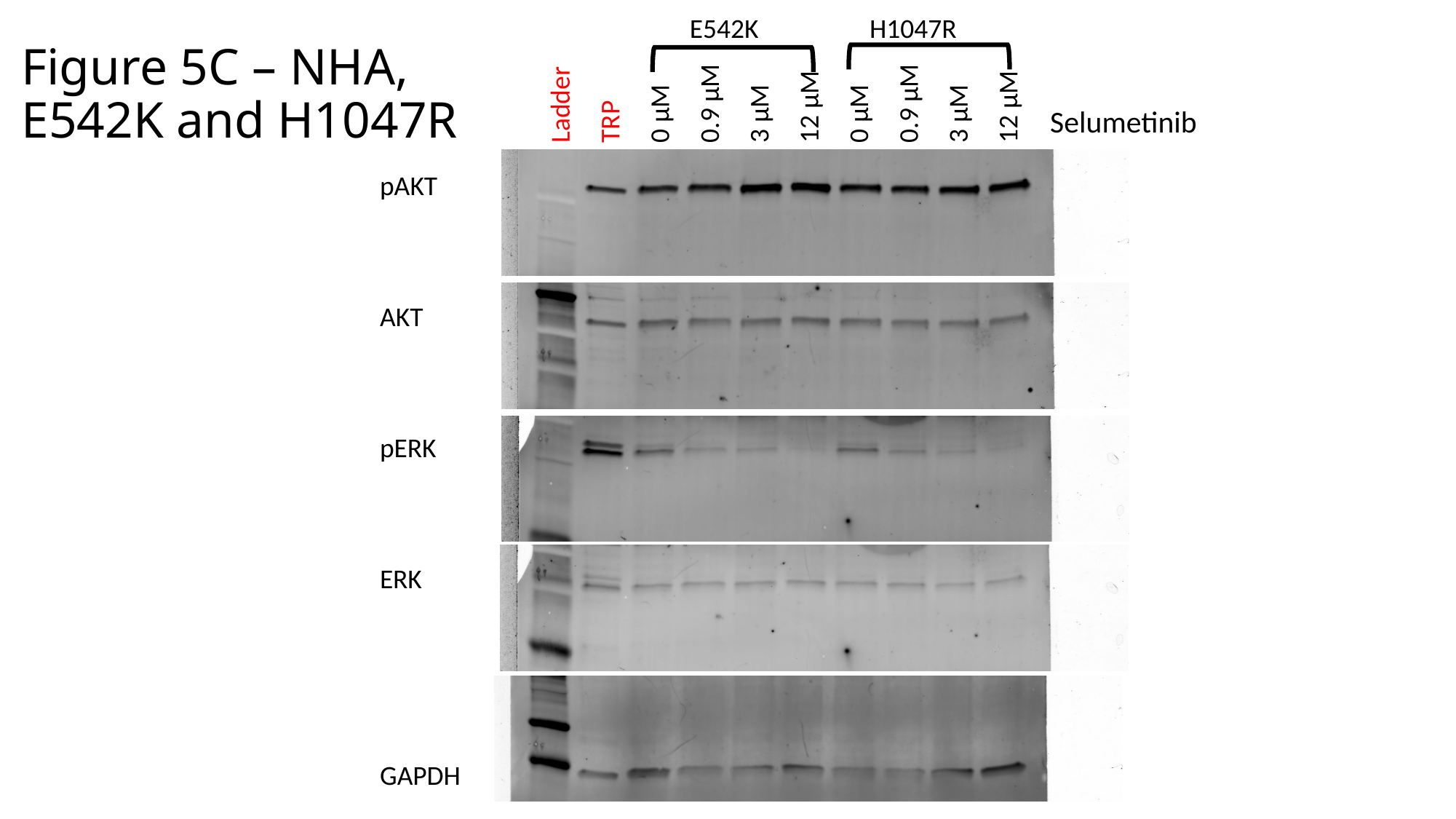

E542K H1047R
Ladder
TRP
0 µM
0.9 µM
3 µM
12 µM
0 µM
0.9 µM
3 µM
12 µM
# Figure 5C – NHA, E542K and H1047R
Selumetinib
pAKT
AKT
pERK
ERK
GAPDH

## Slide 23
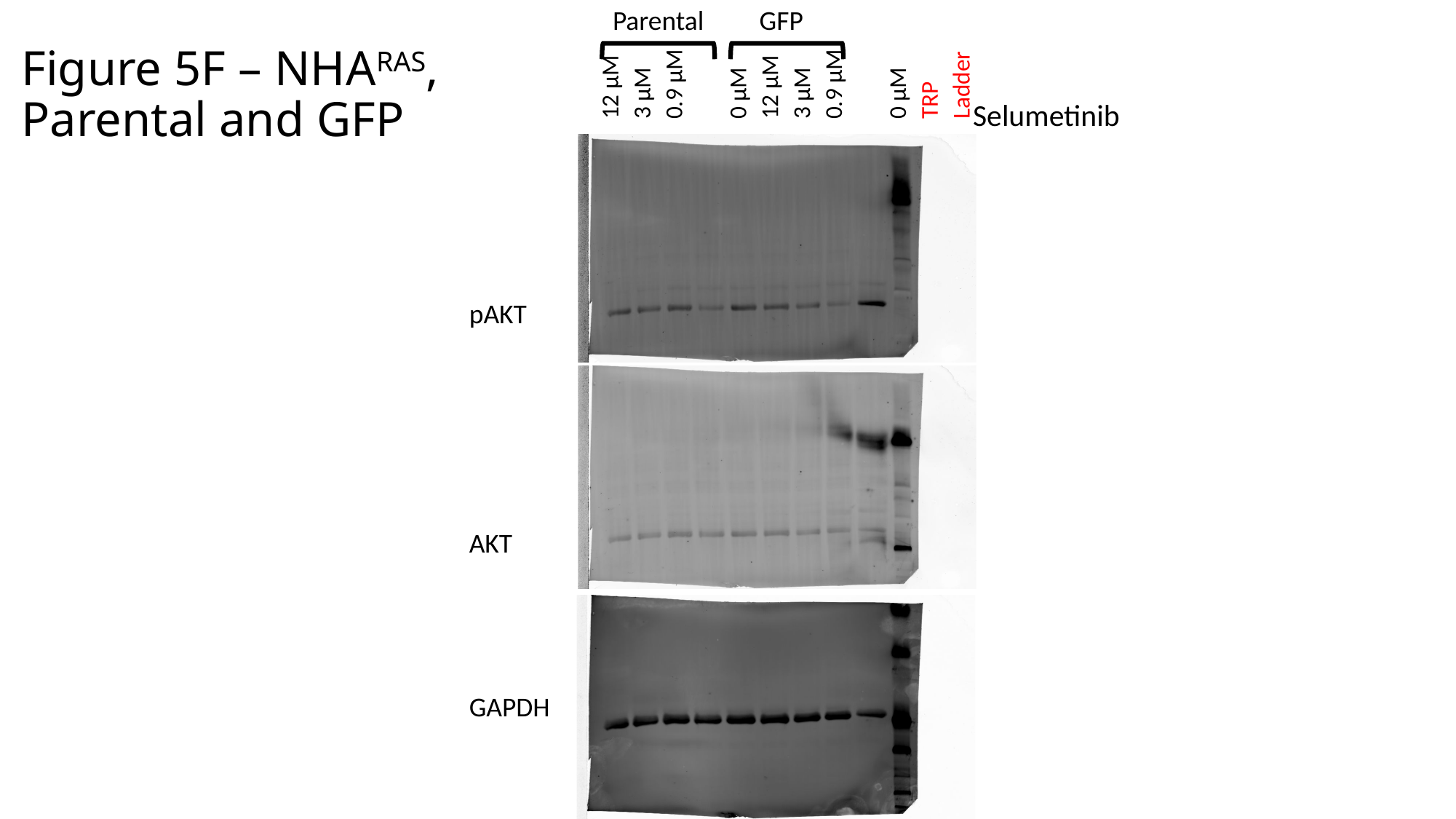

Parental GFP
12 µM
3 µM
0.9 µM
0 µM
12 µM
3 µM
0.9 µM
0 µM
TRP
Ladder
# Figure 5F – NHARAS, Parental and GFP
Selumetinib
pAKT
AKT
GAPDH

## Slide 24
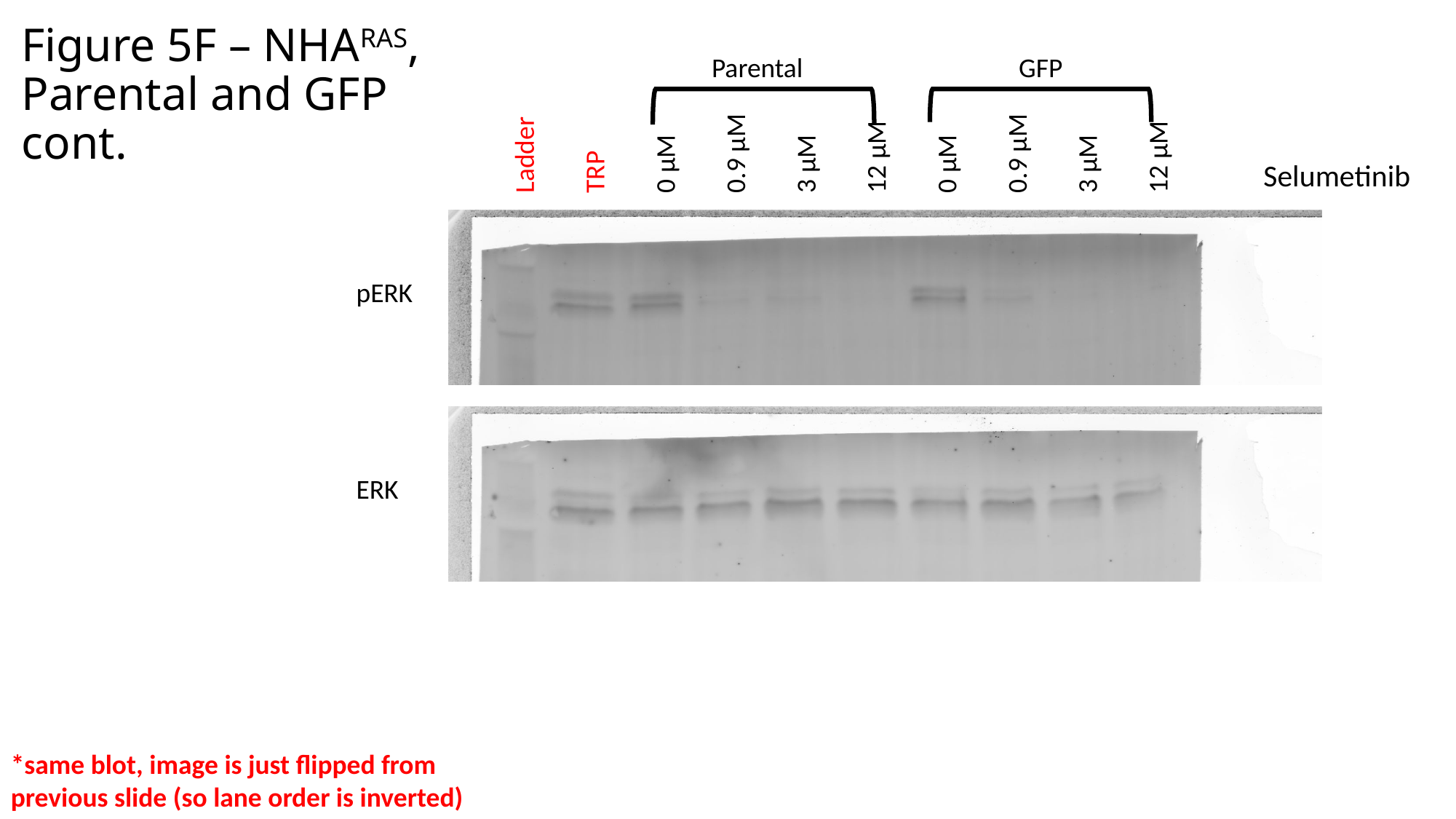

# Figure 5F – NHARAS, Parental and GFP cont.
Parental GFP
Ladder
TRP
0 µM
0.9 µM
3 µM
12 µM
0 µM
0.9 µM
3 µM
12 µM
Selumetinib
pERK
ERK
*same blot, image is just flipped from previous slide (so lane order is inverted)

## Slide 25
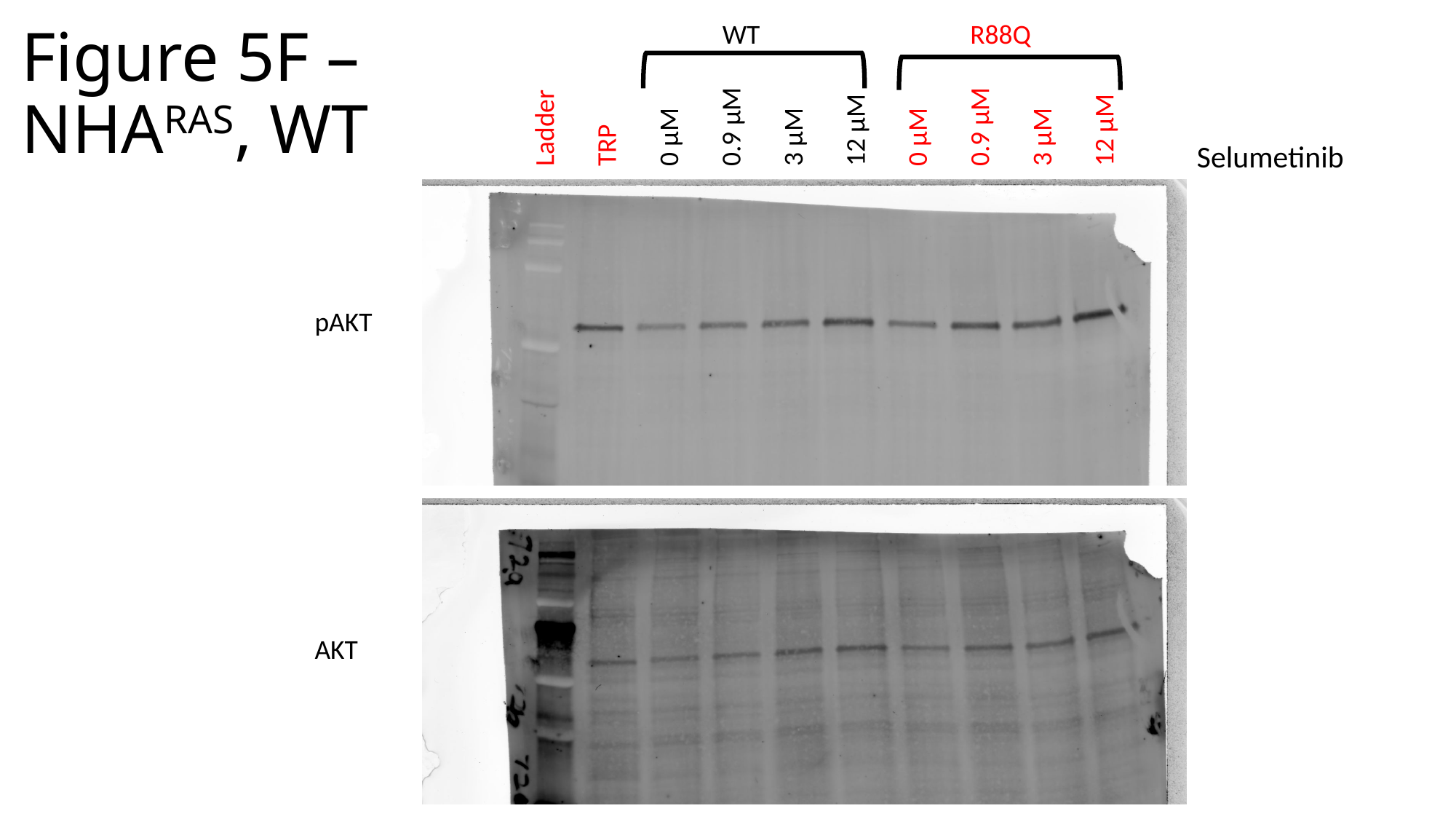

WT R88Q
# Figure 5F – NHARAS, WT
Ladder
TRP
0 µM
0.9 µM
3 µM
12 µM
0 µM
0.9 µM
3 µM
12 µM
Selumetinib
pAKT
AKT

## Slide 26
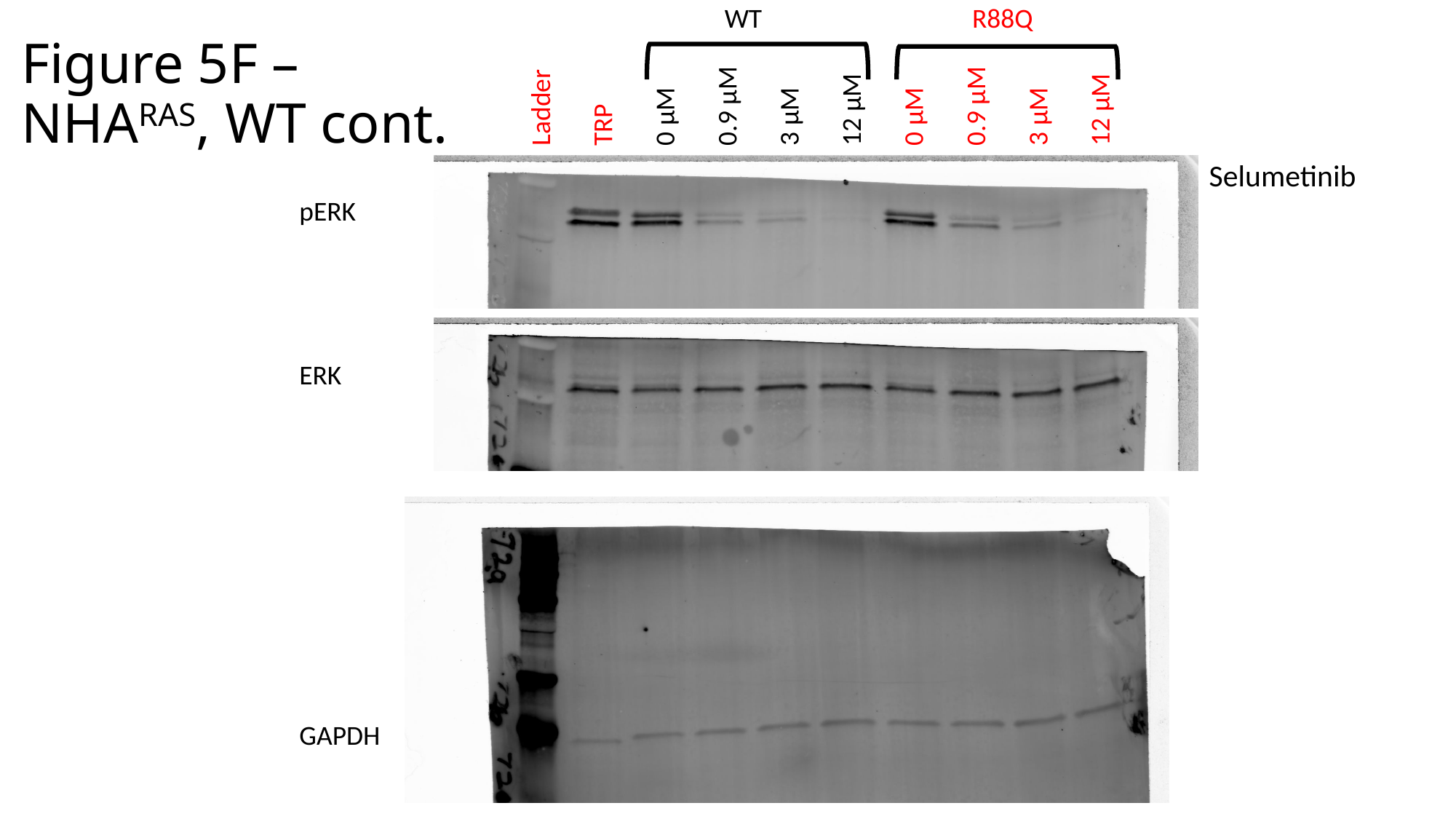

WT R88Q
Ladder
TRP
0 µM
0.9 µM
3 µM
12 µM
0 µM
0.9 µM
3 µM
12 µM
# Figure 5F – NHARAS, WT cont.
Selumetinib
pERK
ERK
GAPDH

## Slide 27
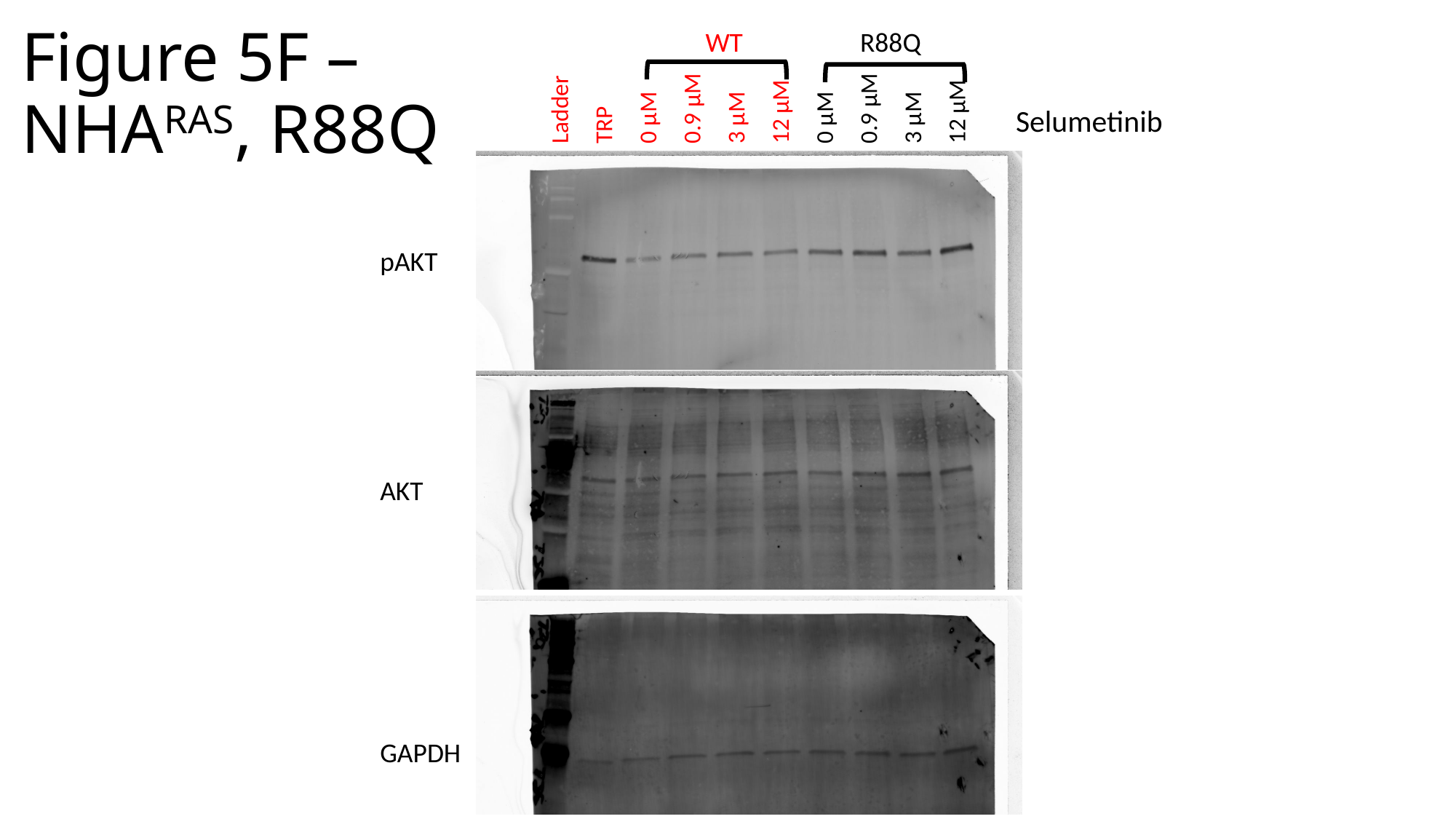

Ladder
TRP
0 µM
0.9 µM
3 µM
12 µM
0 µM
0.9 µM
3 µM
12 µM
# Figure 5F – NHARAS, R88Q
WT R88Q
Selumetinib
pAKT
AKT
GAPDH

## Slide 28
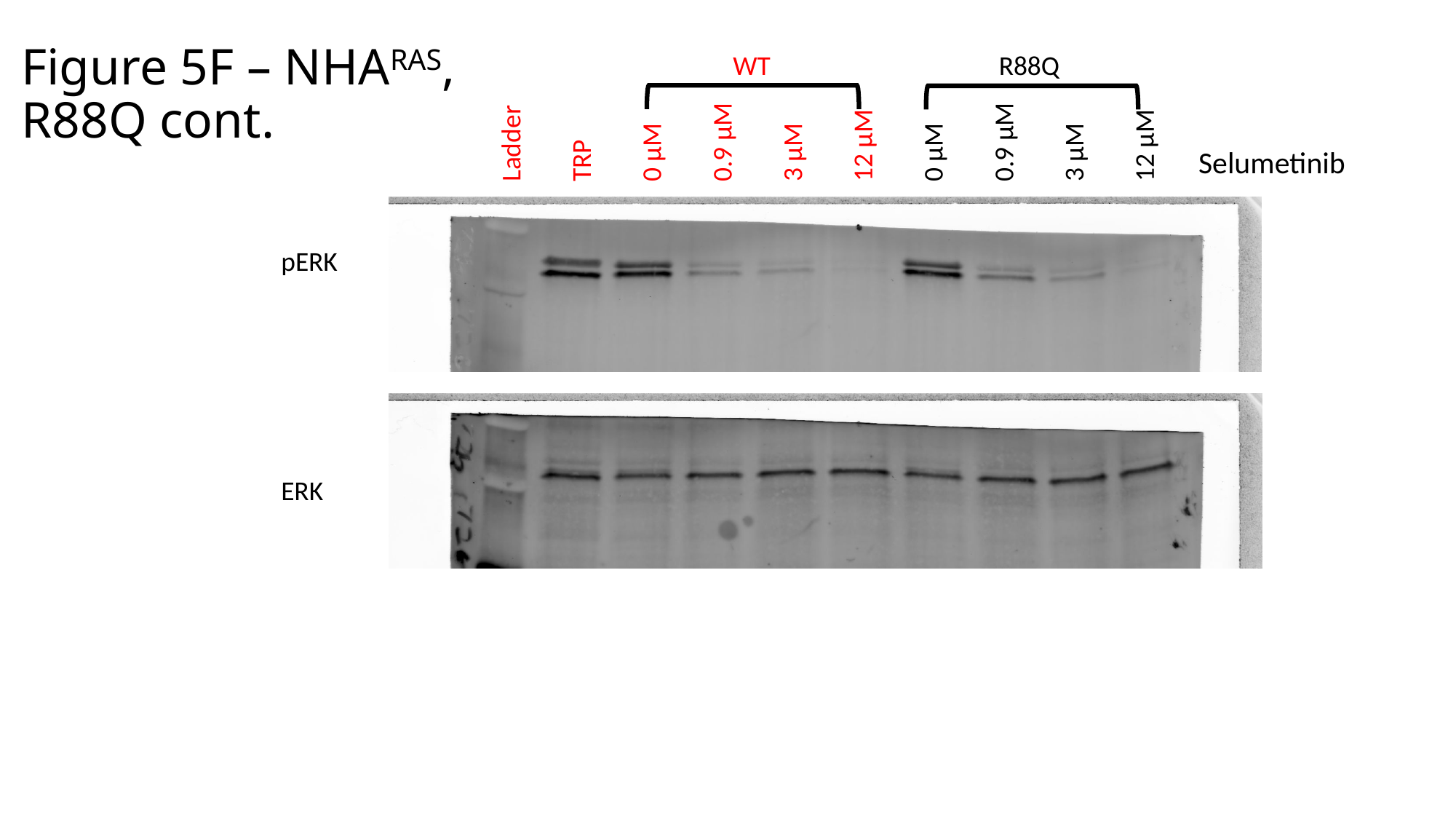

# Figure 5F – NHARAS, R88Q cont.
WT R88Q
Ladder
TRP
0 µM
0.9 µM
3 µM
12 µM
0 µM
0.9 µM
3 µM
12 µM
Selumetinib
pERK
ERK

## Slide 29
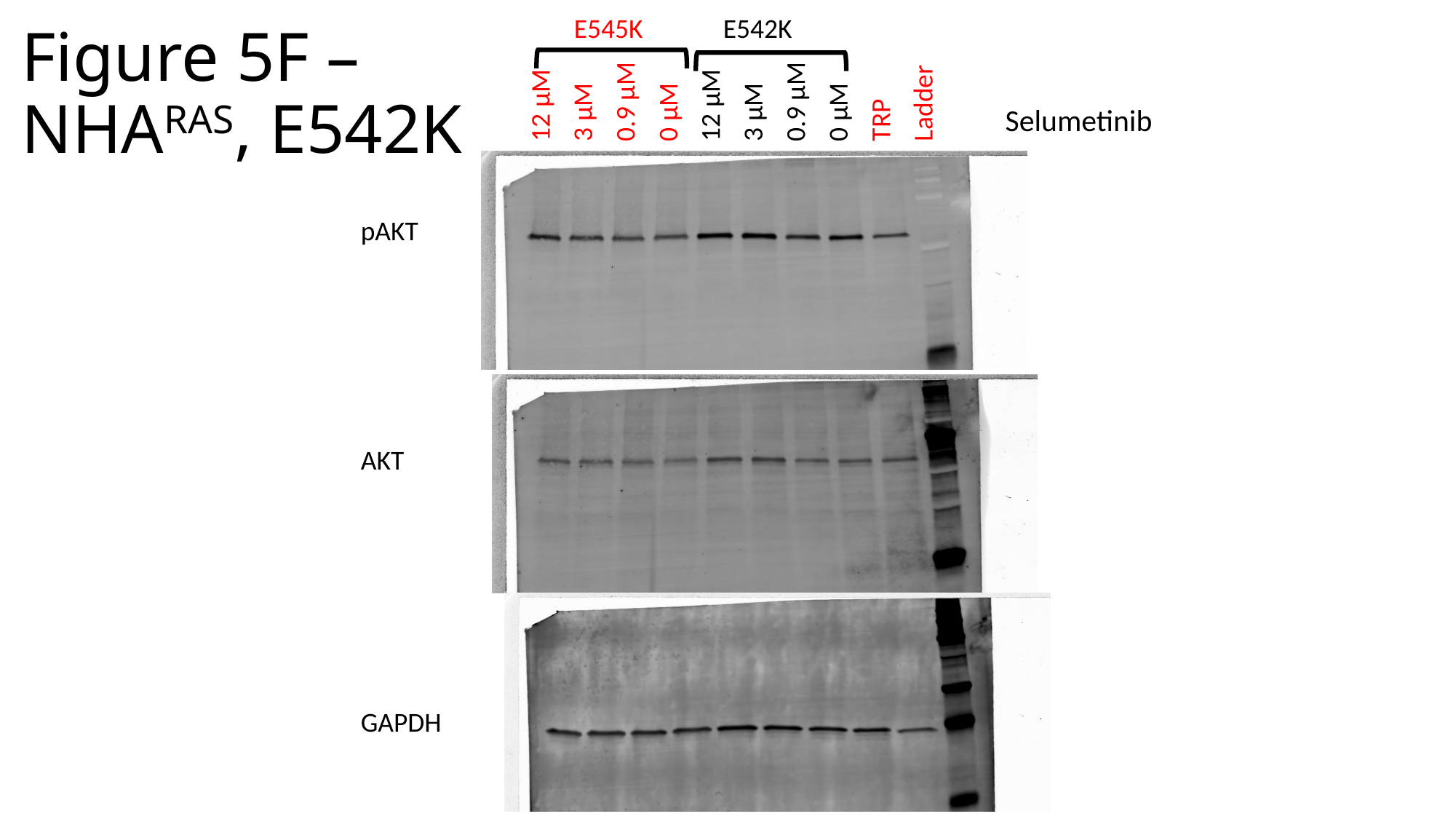

E545K E542K
12 µM
3 µM
0.9 µM
0 µM
12 µM
3 µM
0.9 µM
0 µM
TRP
Ladder
# Figure 5F – NHARAS, E542K
Selumetinib
pAKT
AKT
GAPDH

## Slide 30
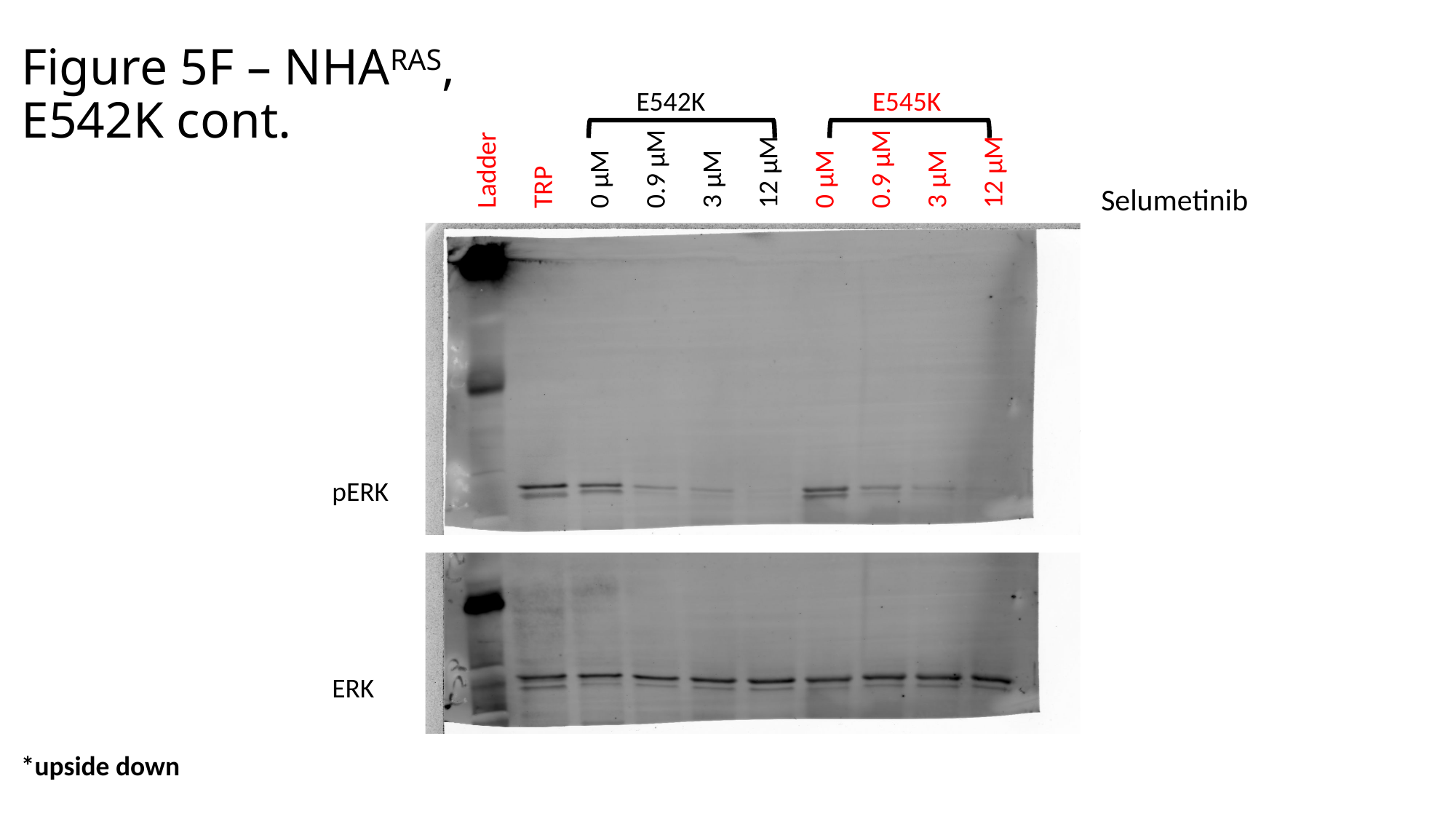

# Figure 5F – NHARAS, E542K cont.
Ladder
TRP
0 µM
0.9 µM
3 µM
12 µM
0 µM
0.9 µM
3 µM
12 µM
E542K E545K
Selumetinib
pERK
ERK
*upside down

## Slide 31
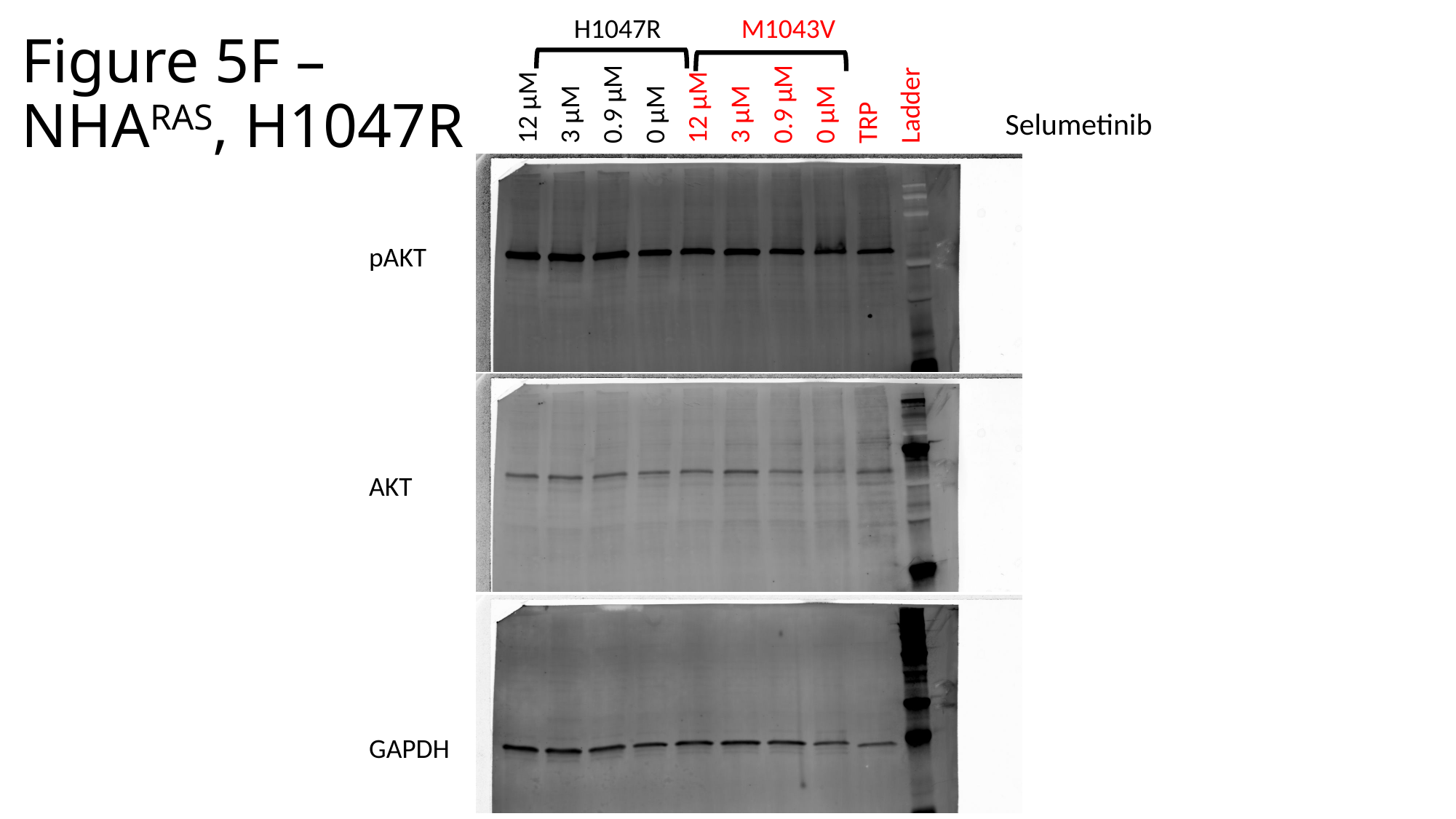

H1047R M1043V
12 µM
3 µM
0.9 µM
0 µM
12 µM
3 µM
0.9 µM
0 µM
TRP
Ladder
# Figure 5F – NHARAS, H1047R
Selumetinib
pAKT
AKT
GAPDH

## Slide 32
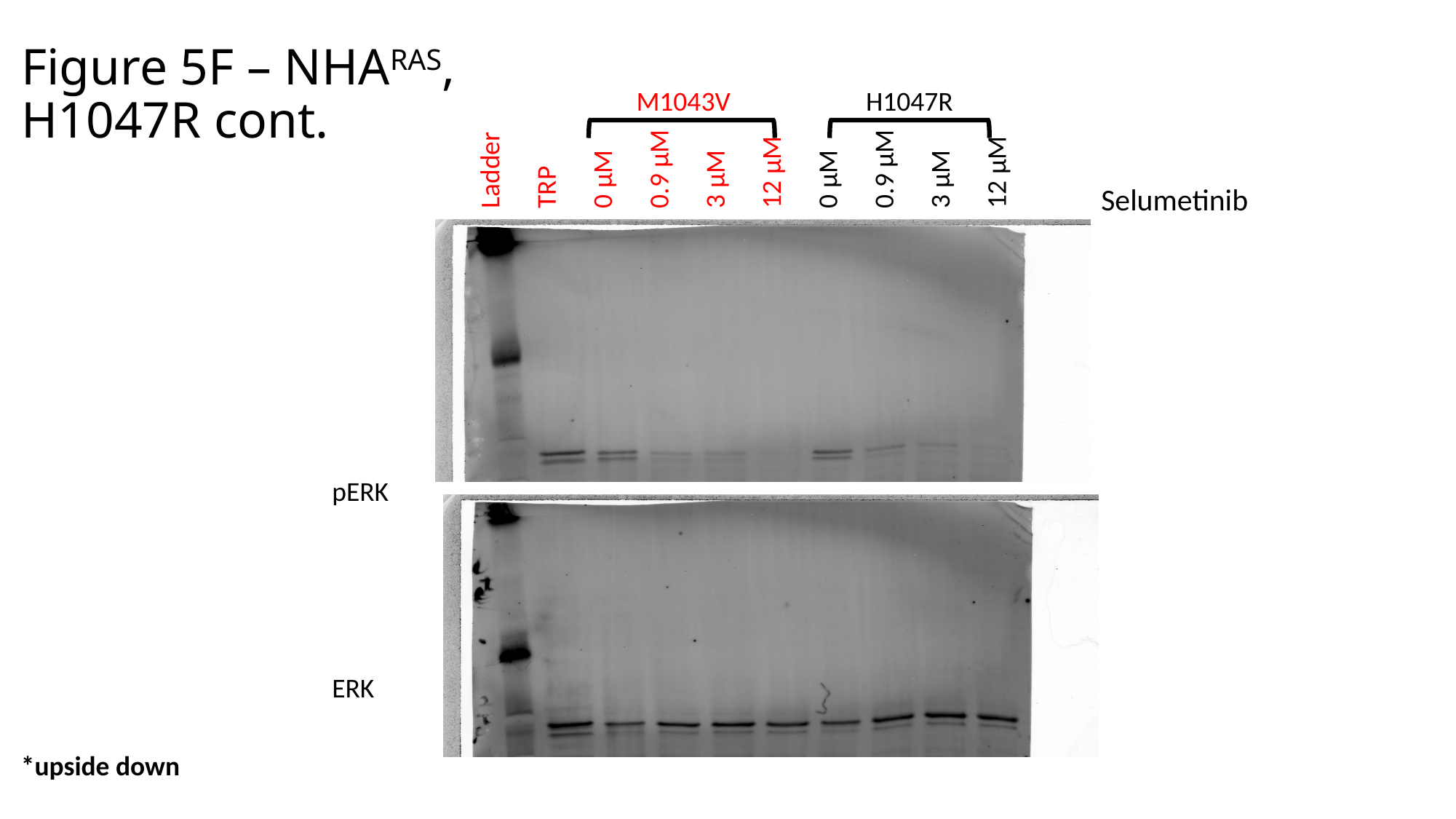

# Figure 5F – NHARAS, H1047R cont.
Ladder
TRP
0 µM
0.9 µM
3 µM
12 µM
0 µM
0.9 µM
3 µM
12 µM
M1043V H1047R
Selumetinib
pERK
ERK
*upside down

## Slide 33
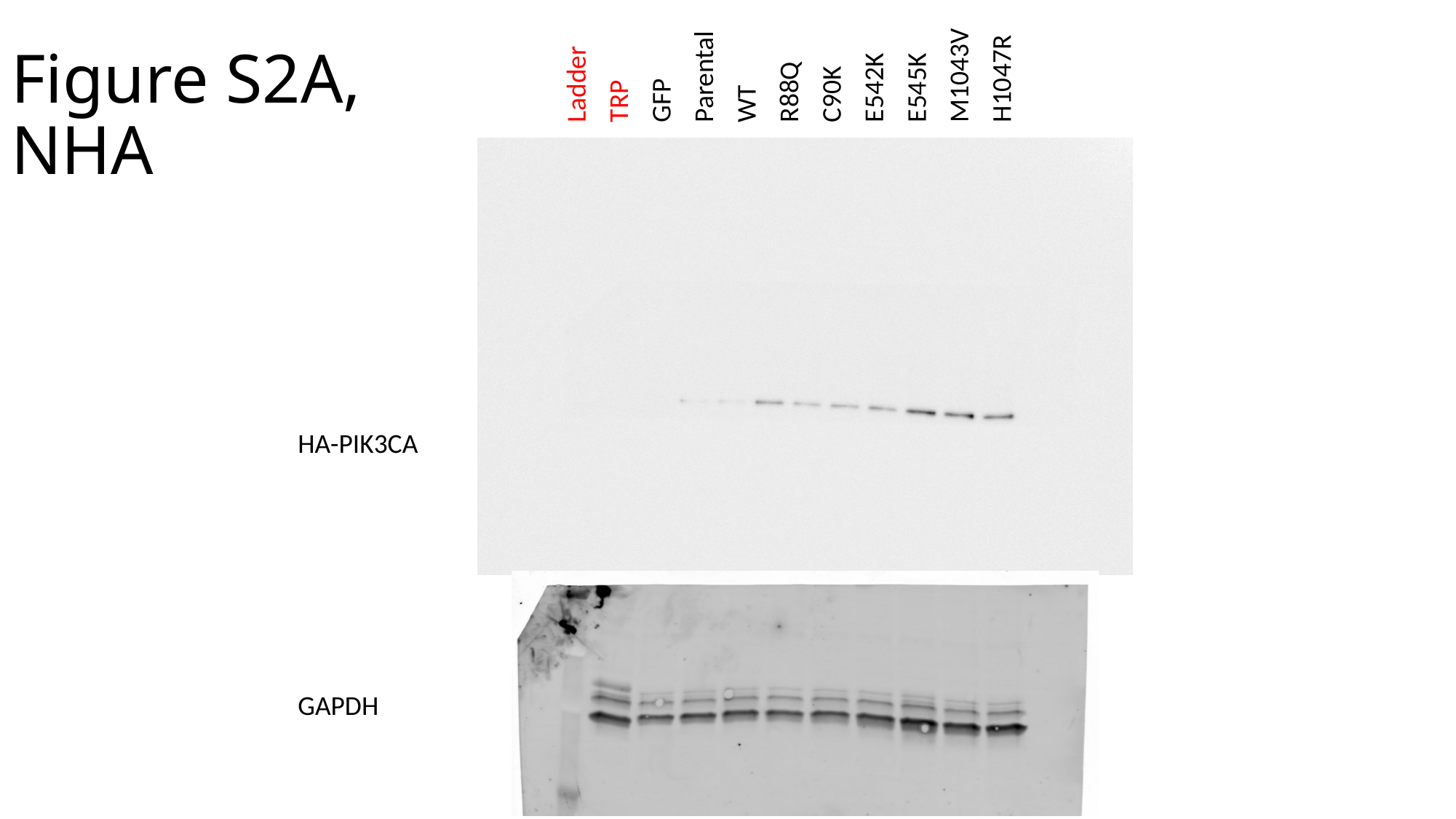

# Figure S2A, NHA
Ladder
TRP
GFP
Parental
WT
R88Q
C90K
E542K
E545K
M1043V
H1047R
HA-PIK3CA
GAPDH

## Slide 34
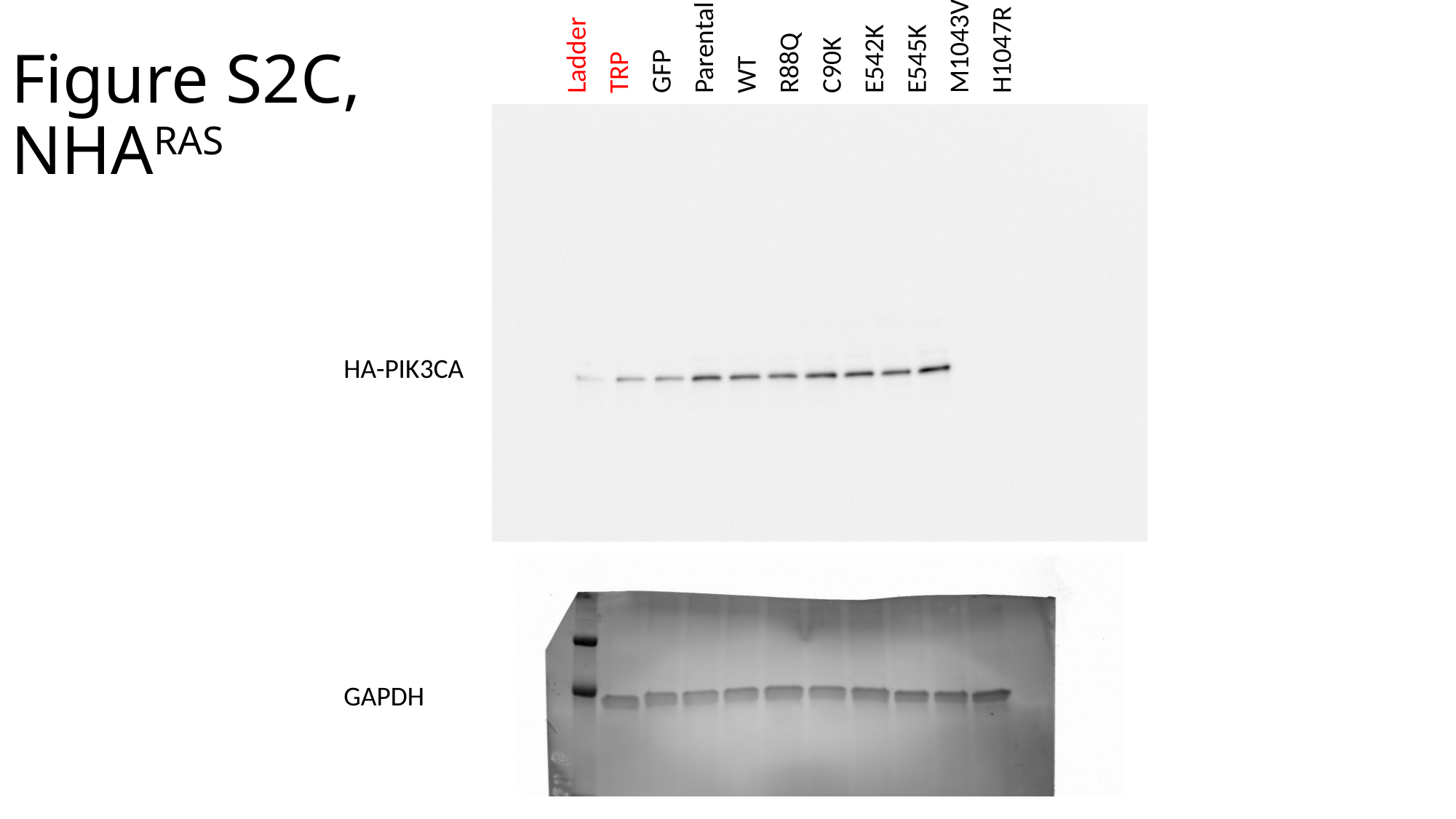

# Figure S2C, NHARAS
Ladder
TRP
GFP
Parental
WT
R88Q
C90K
E542K
E545K
M1043V
H1047R
HA-PIK3CA
GAPDH

## Slide 35
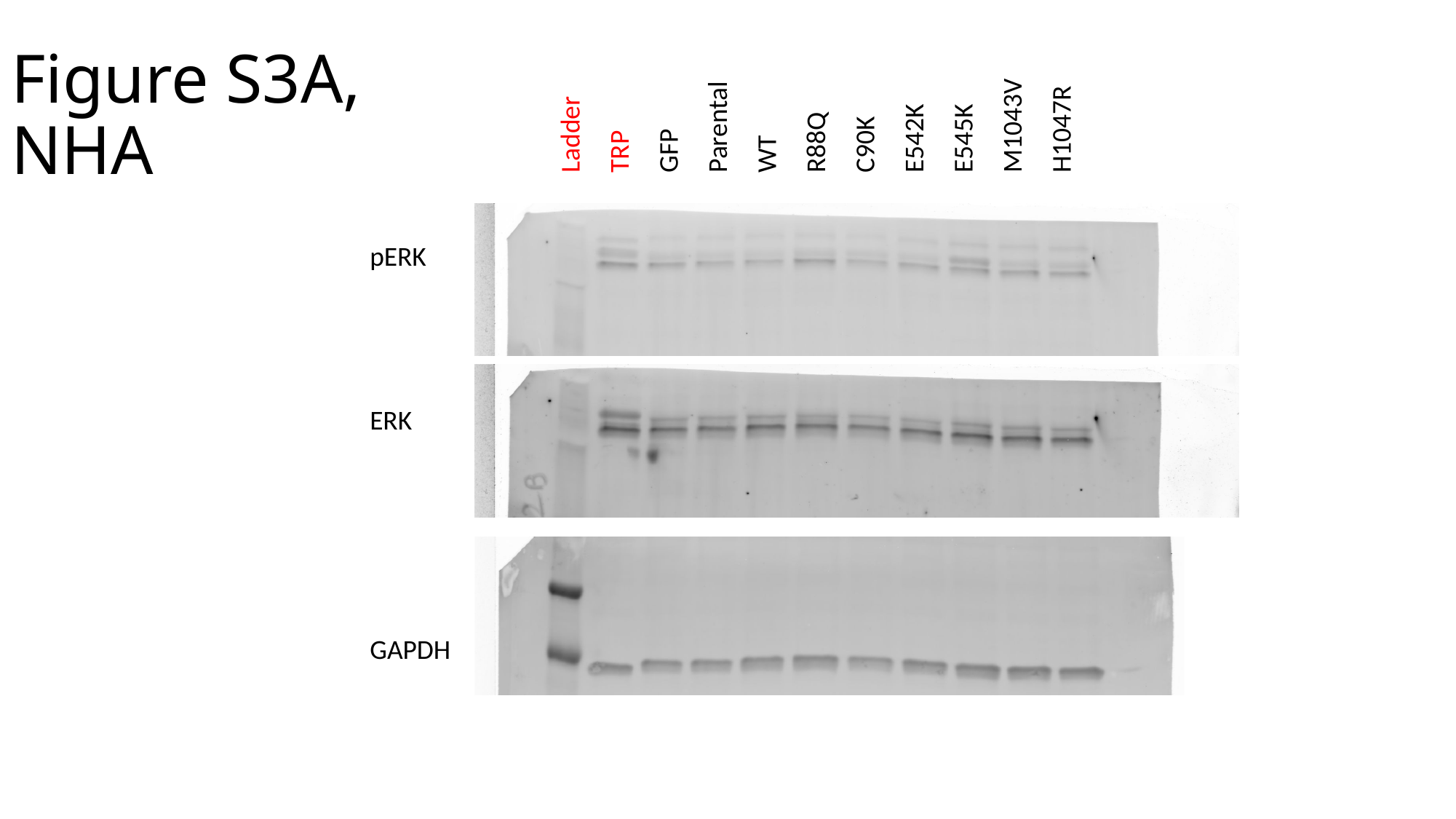

# Figure S3A, NHA
Ladder
TRP
GFP
Parental
WT
R88Q
C90K
E542K
E545K
M1043V
H1047R
pERK
ERK
GAPDH

## Slide 36
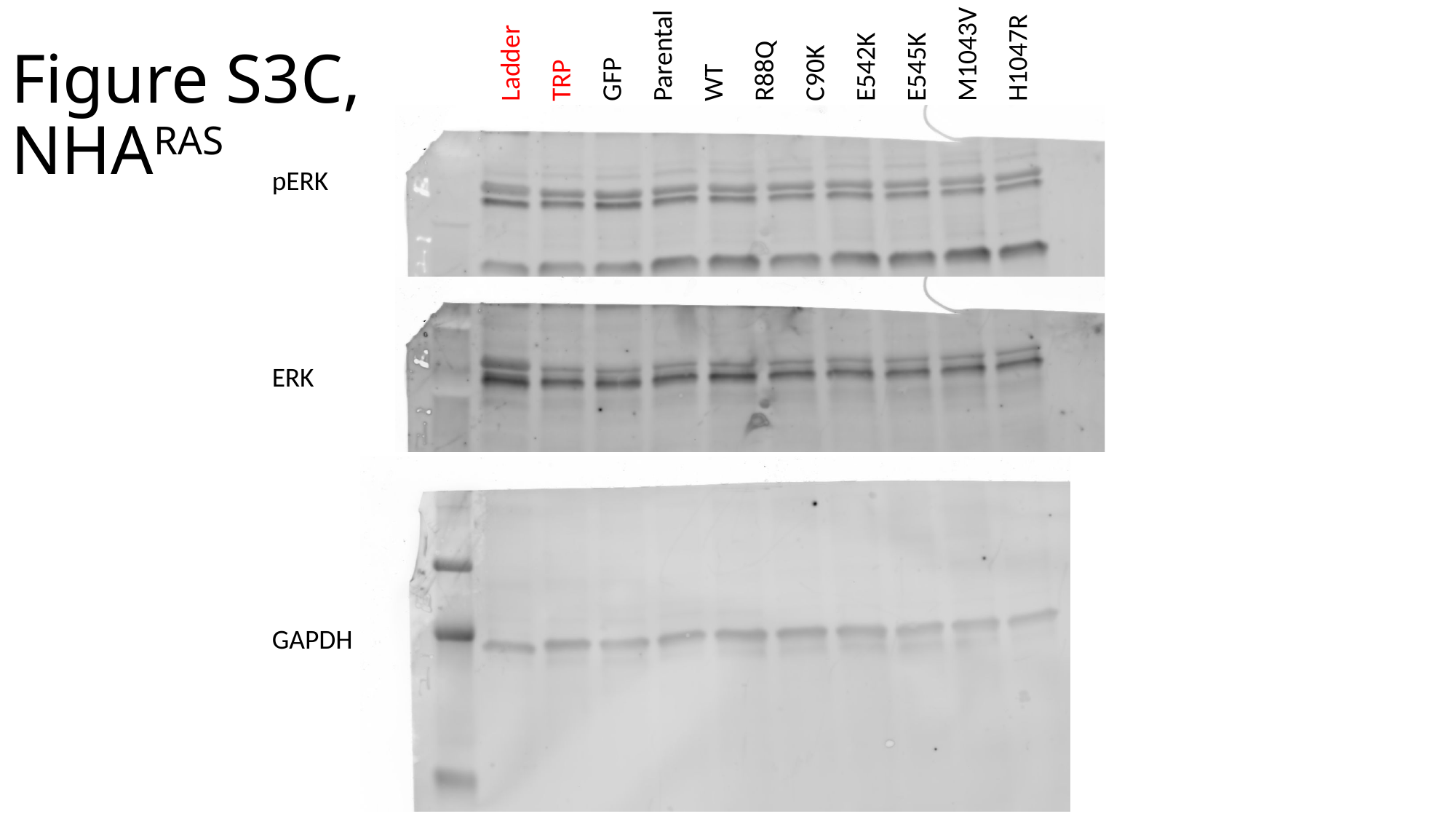

# Figure S3C, NHARAS
Ladder
TRP
GFP
Parental
WT
R88Q
C90K
E542K
E545K
M1043V
H1047R
pERK
ERK
GAPDH

## Slide 37
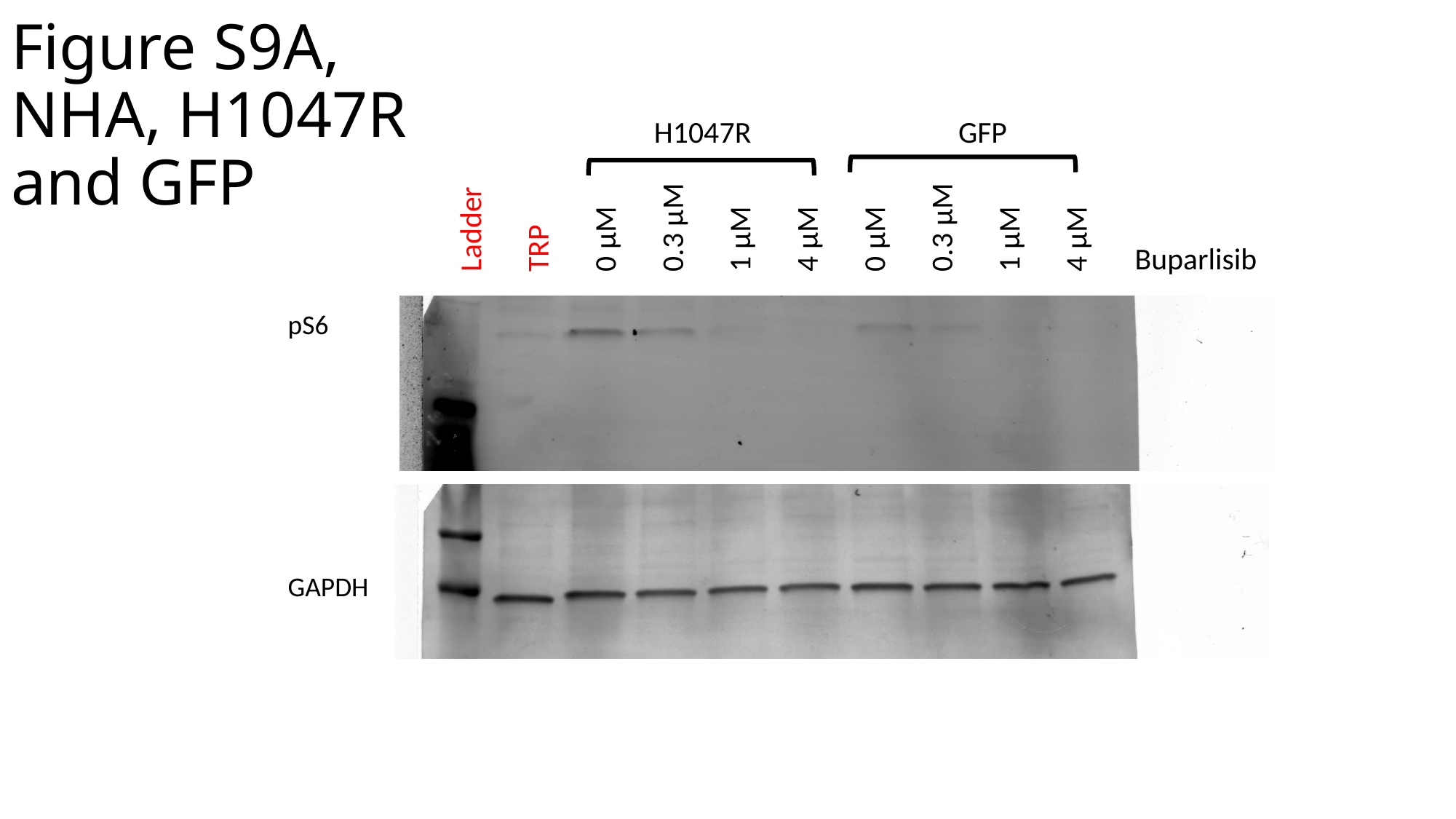

# Figure S9A, NHA, H1047R and GFP
 H1047R GFP
Ladder
TRP
0 µM
0.3 µM
1 µM
4 µM
0 µM
0.3 µM
1 µM
4 µM
Buparlisib
pS6
GAPDH

## Slide 38
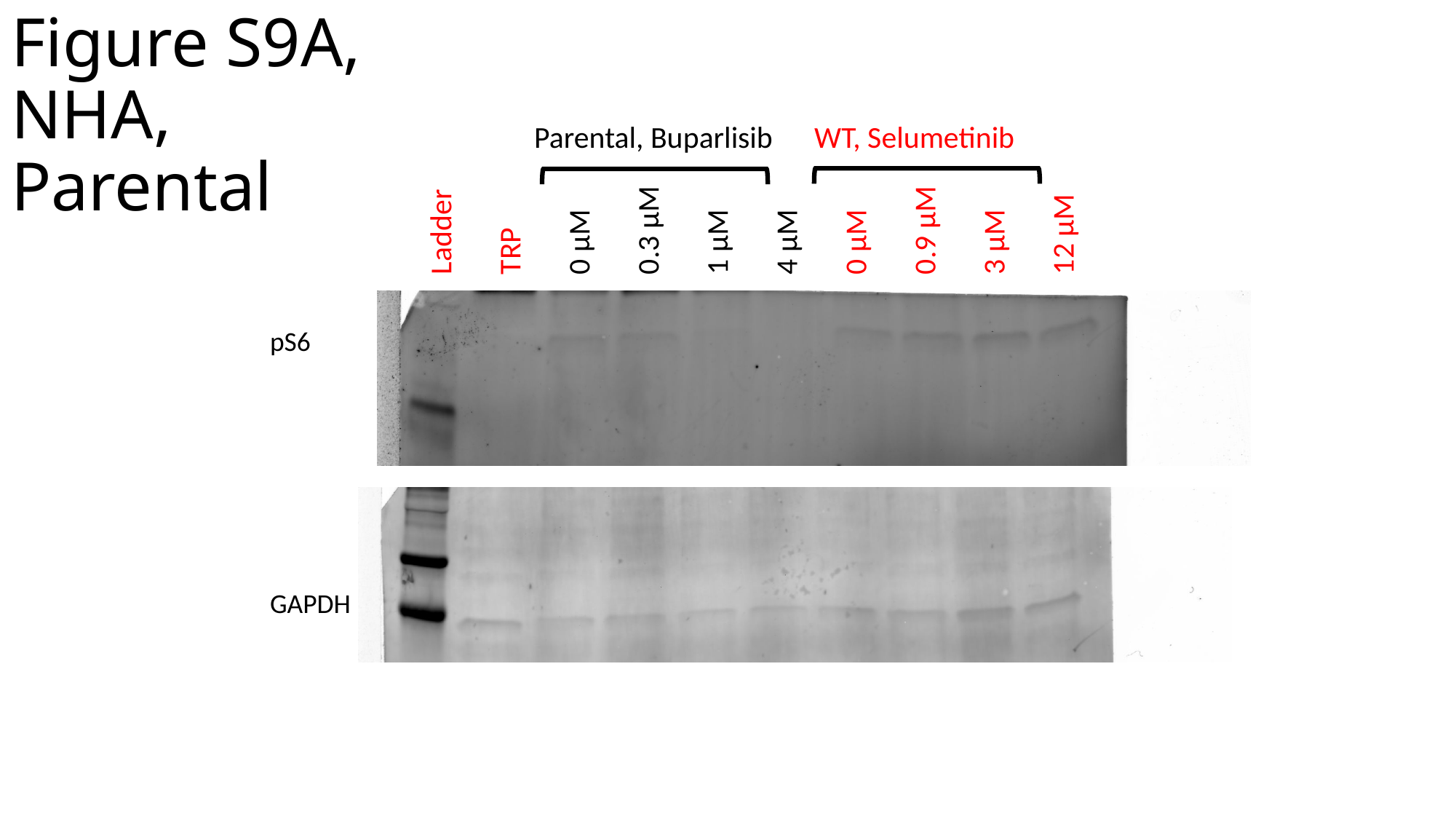

# Figure S9A, NHA, Parental
 Parental, Buparlisib WT, Selumetinib
Ladder
TRP
0 µM
0.3 µM
1 µM
4 µM
0 µM
0.9 µM
3 µM
12 µM
pS6
GAPDH

## Slide 39
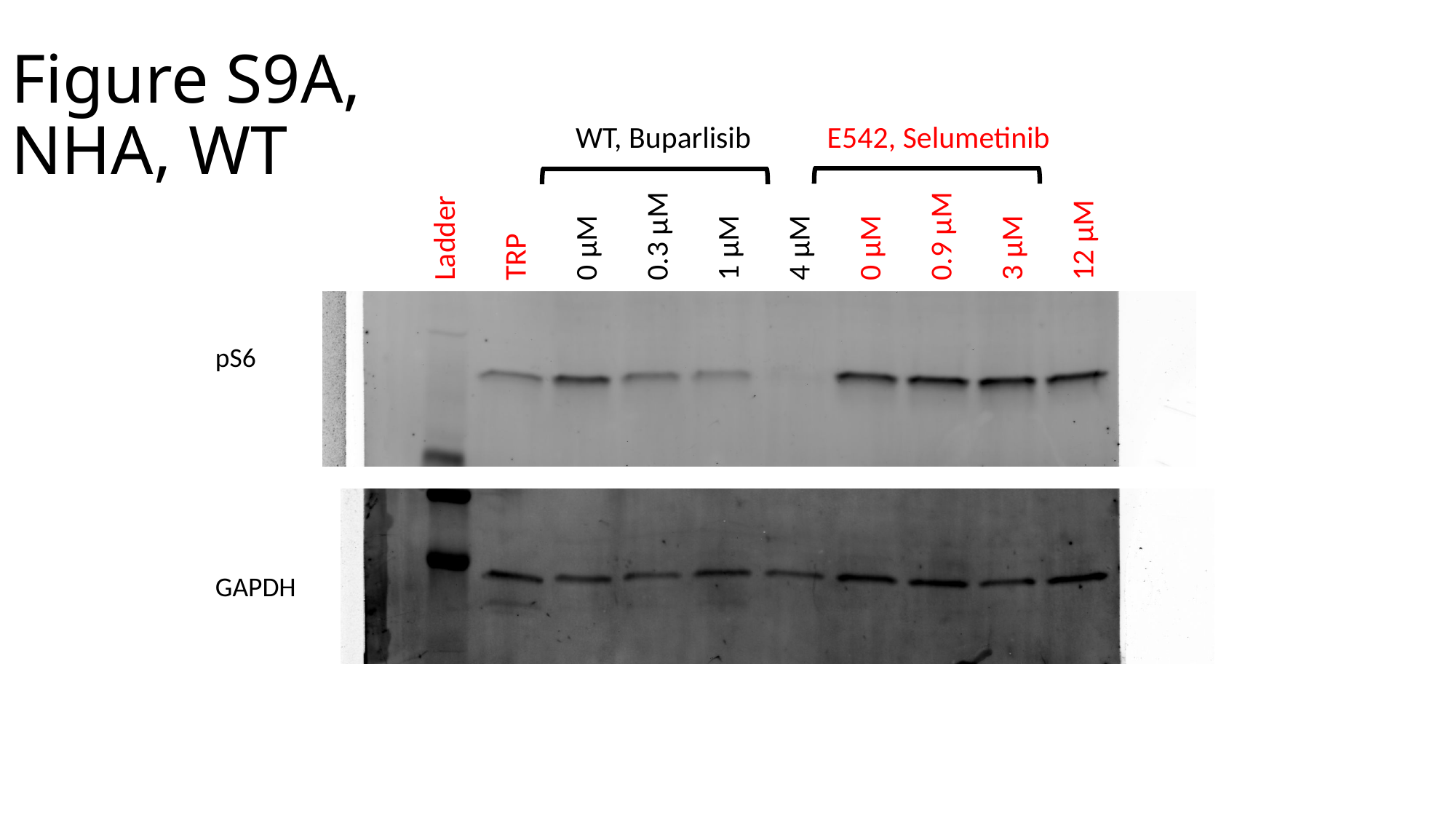

# Figure S9A, NHA, WT
 WT, Buparlisib E542, Selumetinib
Ladder
TRP
0 µM
0.3 µM
1 µM
4 µM
0 µM
0.9 µM
3 µM
12 µM
pS6
GAPDH

## Slide 40
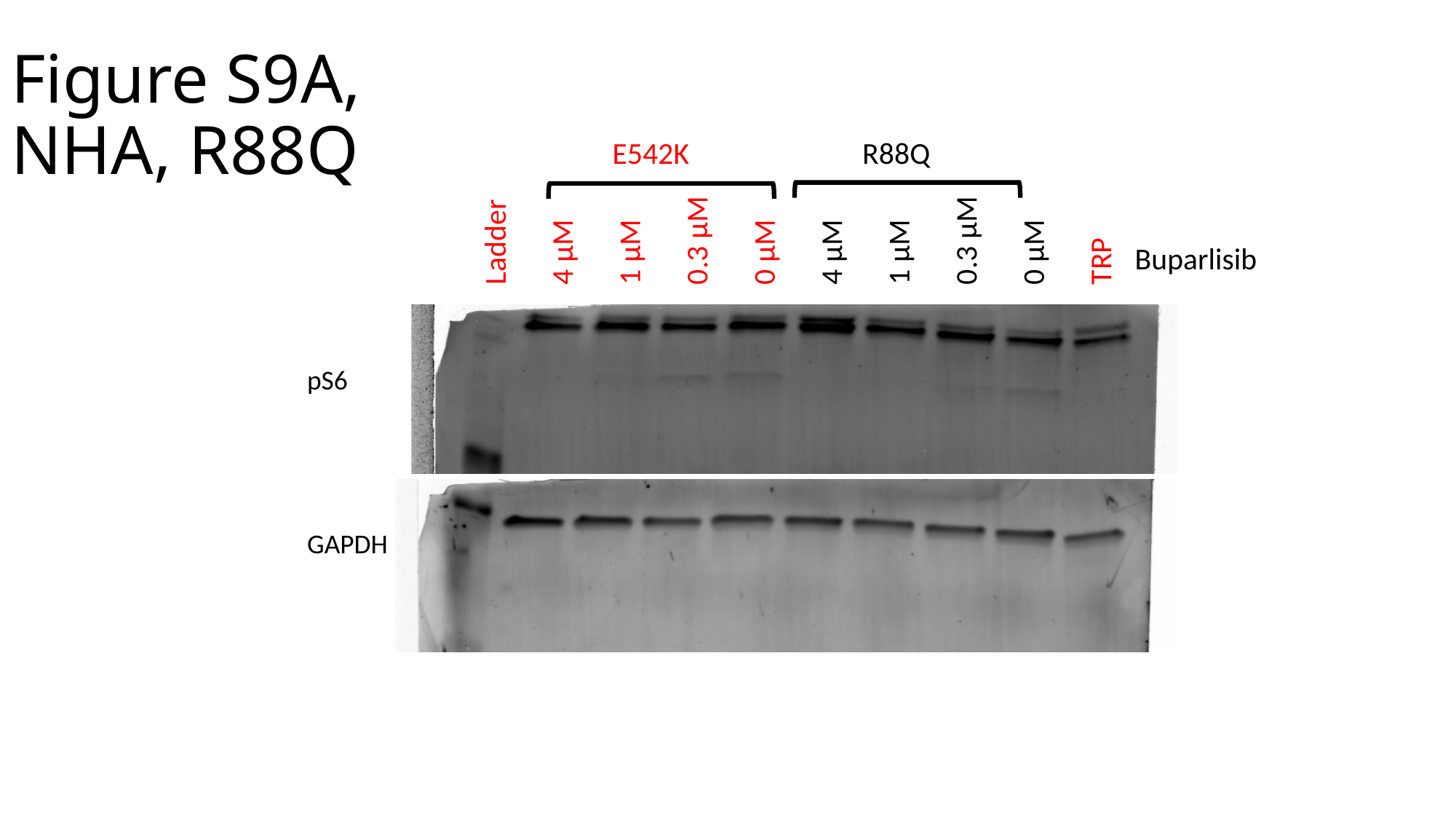

# Figure S9A, NHA, R88Q
 E542K R88Q
Ladder
4 µM
1 µM
0.3 µM
0 µM
4 µM
1 µM
0.3 µM
0 µM
TRP
Buparlisib
pS6
GAPDH

## Slide 41
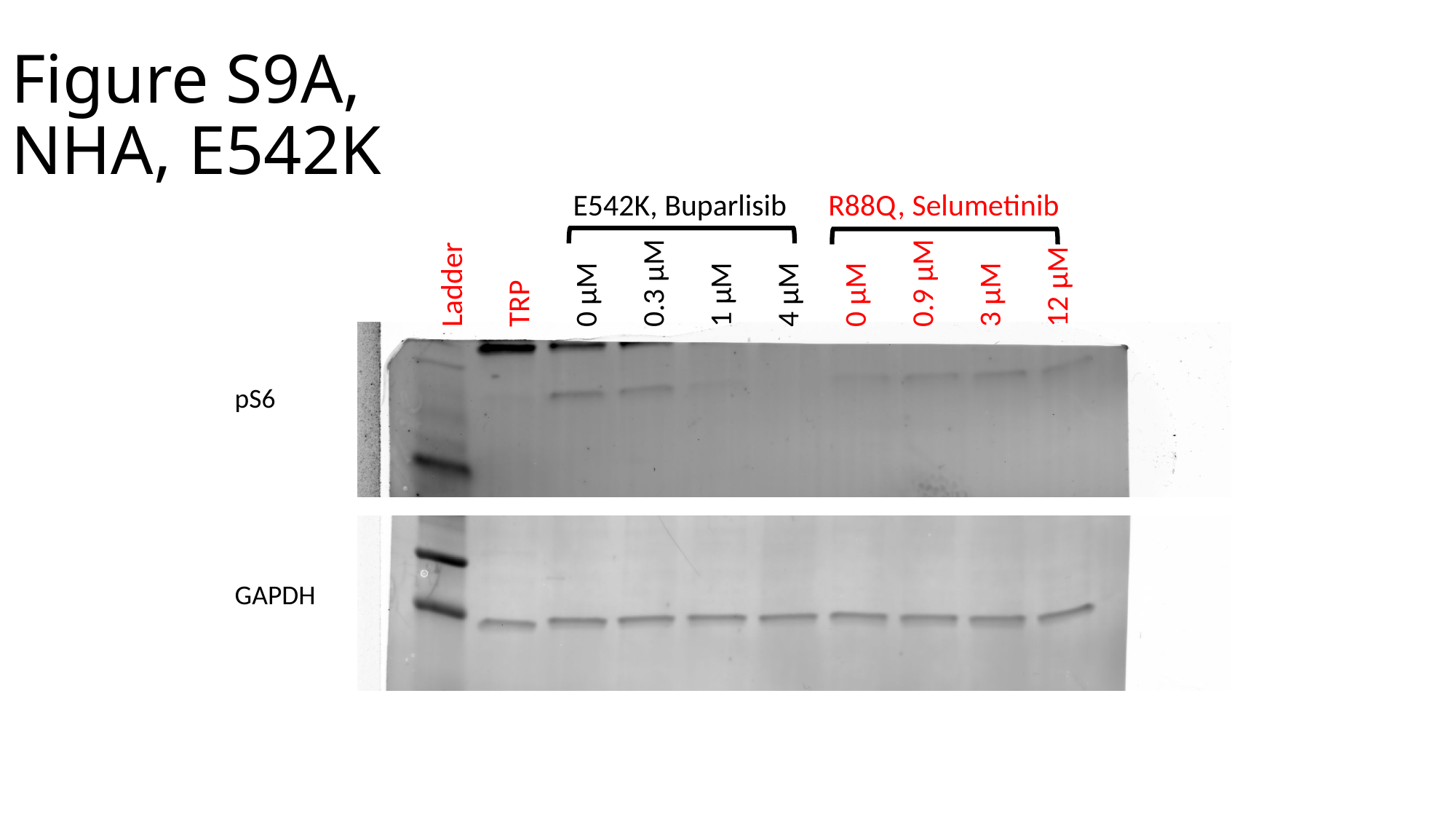

# Figure S9A, NHA, E542K
 E542K, Buparlisib R88Q, Selumetinib
Ladder
TRP
0 µM
0.3 µM
1 µM
4 µM
0 µM
0.9 µM
3 µM
12 µM
pS6
GAPDH

## Slide 42
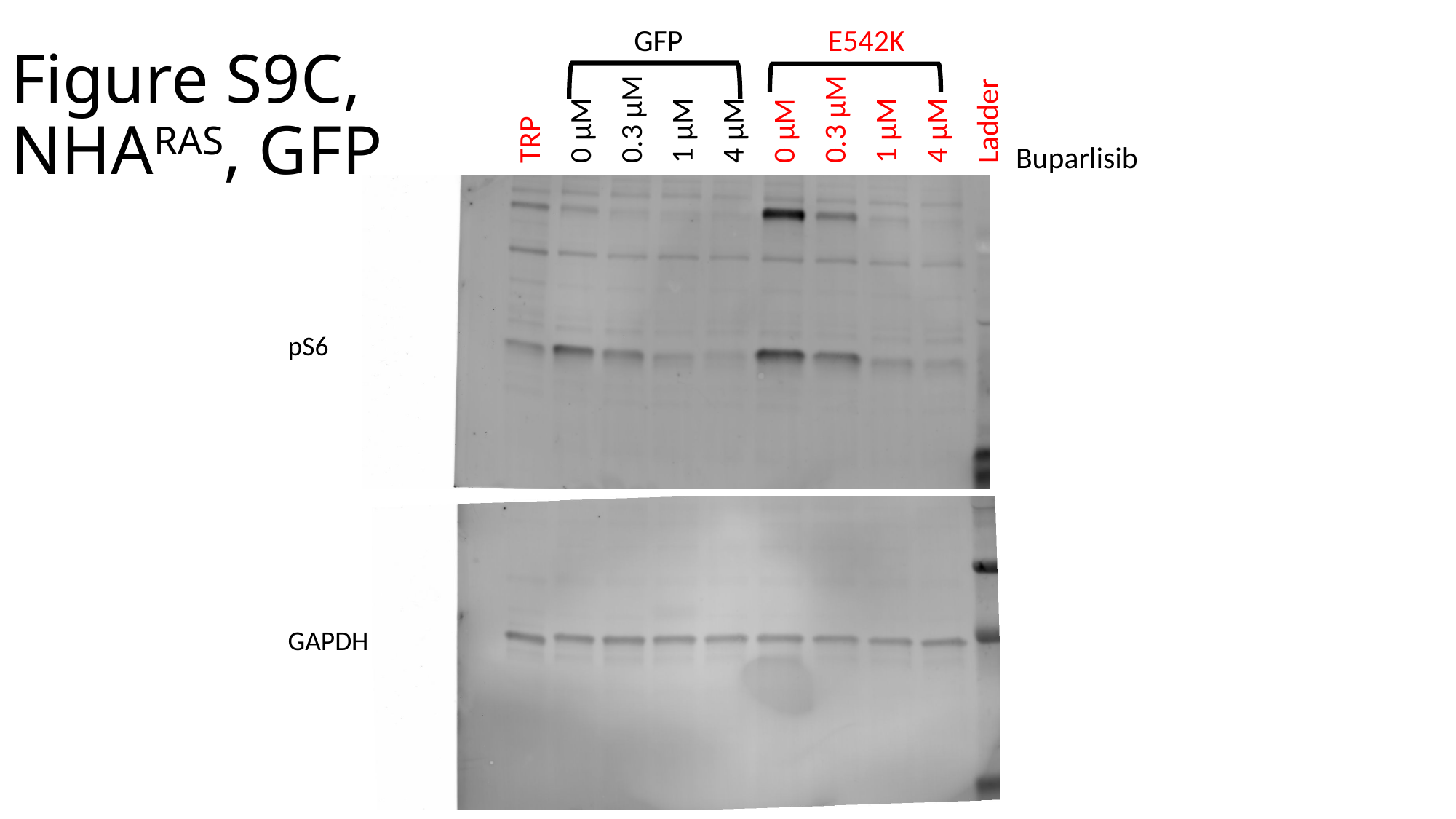

# Figure S9C, NHARAS, GFP
 GFP E542K
TRP
0 µM
0.3 µM
1 µM
4 µM
0 µM
0.3 µM
1 µM
4 µM
Ladder
Buparlisib
pS6
GAPDH

## Slide 43
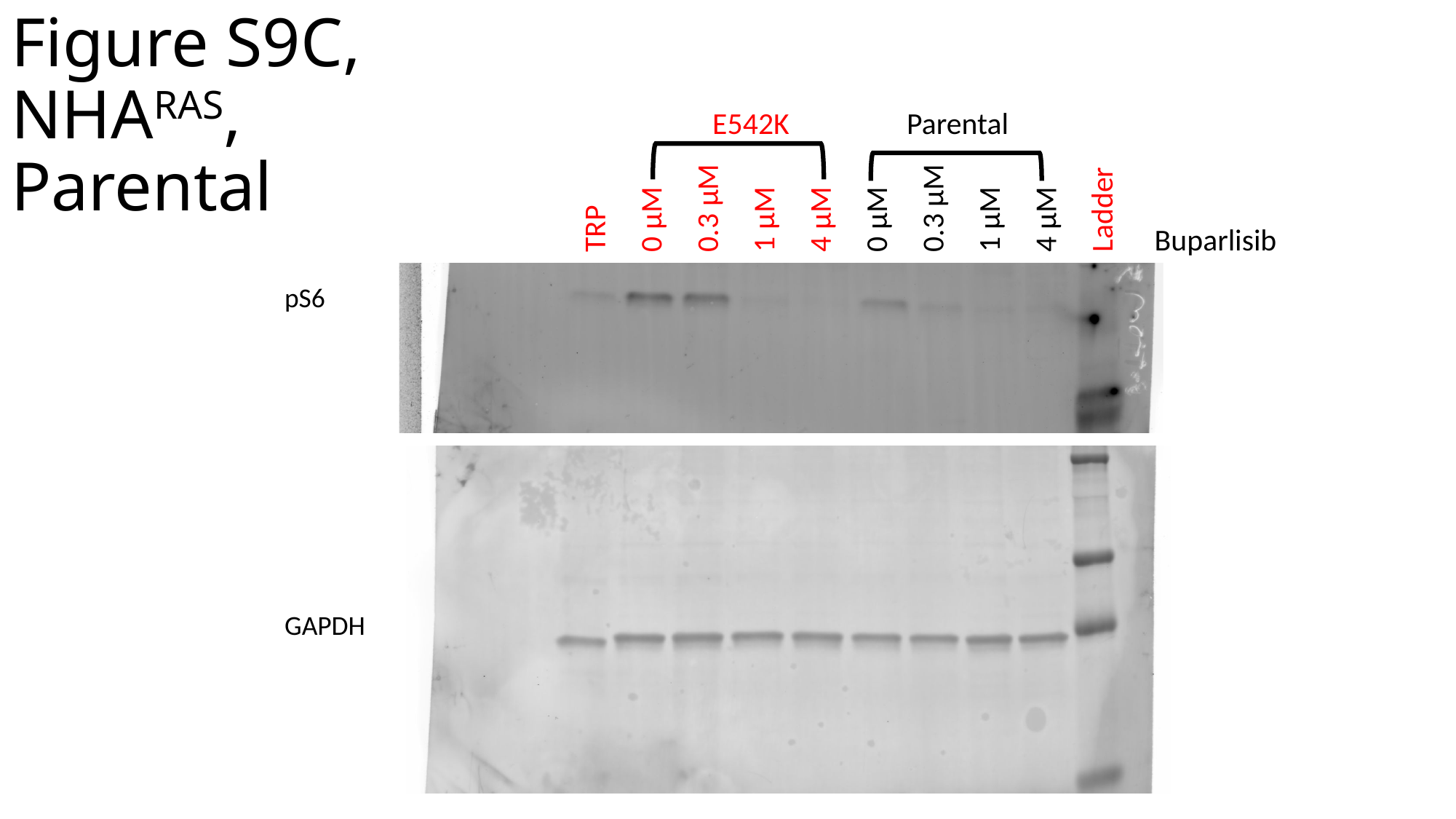

# Figure S9C, NHARAS, Parental
 E542K Parental
TRP
0 µM
0.3 µM
1 µM
4 µM
0 µM
0.3 µM
1 µM
4 µM
Ladder
Buparlisib
pS6
GAPDH

## Slide 44
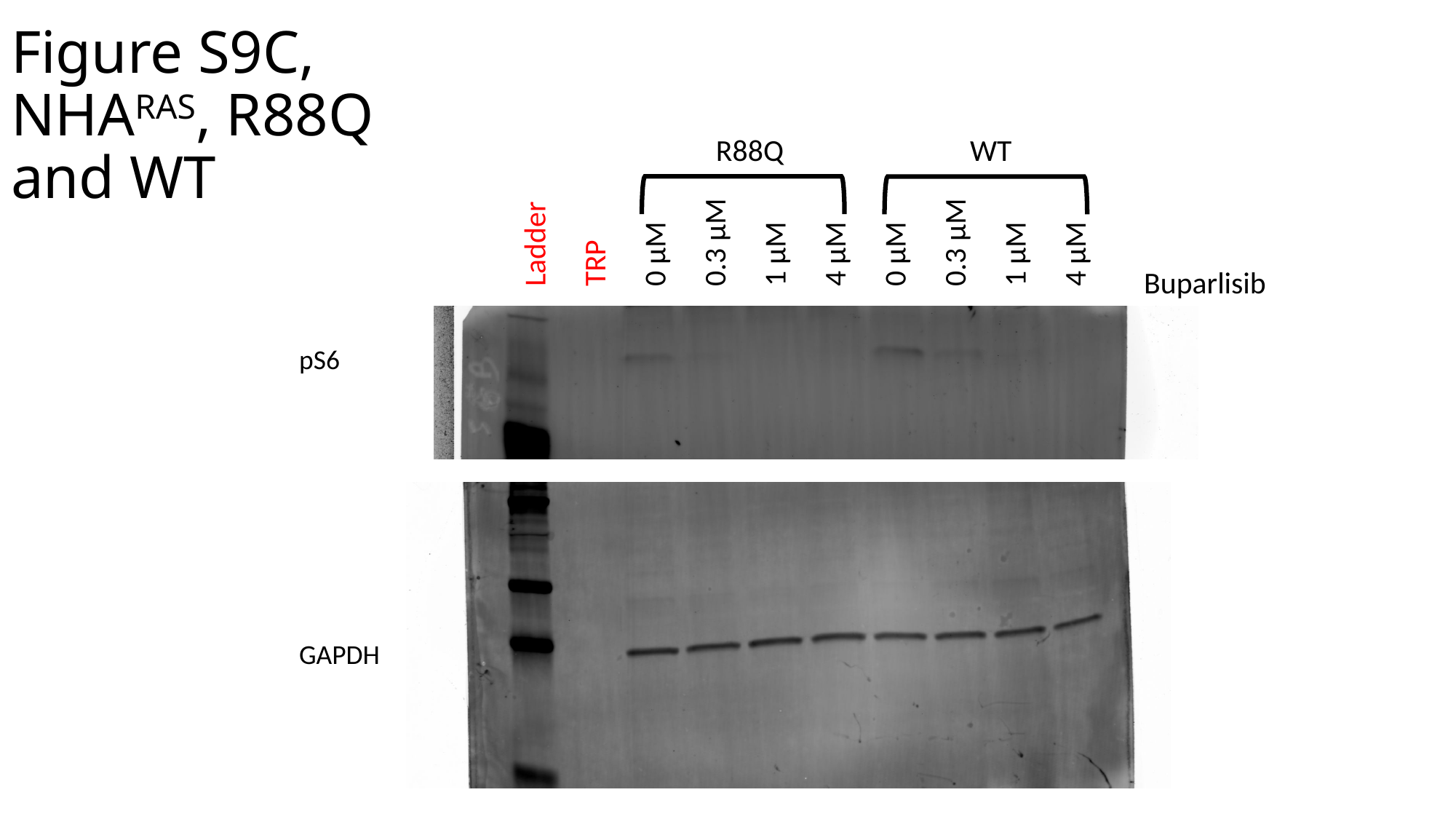

# Figure S9C, NHARAS, R88Q and WT
 R88Q WT
Ladder
TRP
0 µM
0.3 µM
1 µM
4 µM
0 µM
0.3 µM
1 µM
4 µM
Buparlisib
pS6
GAPDH

## Slide 45
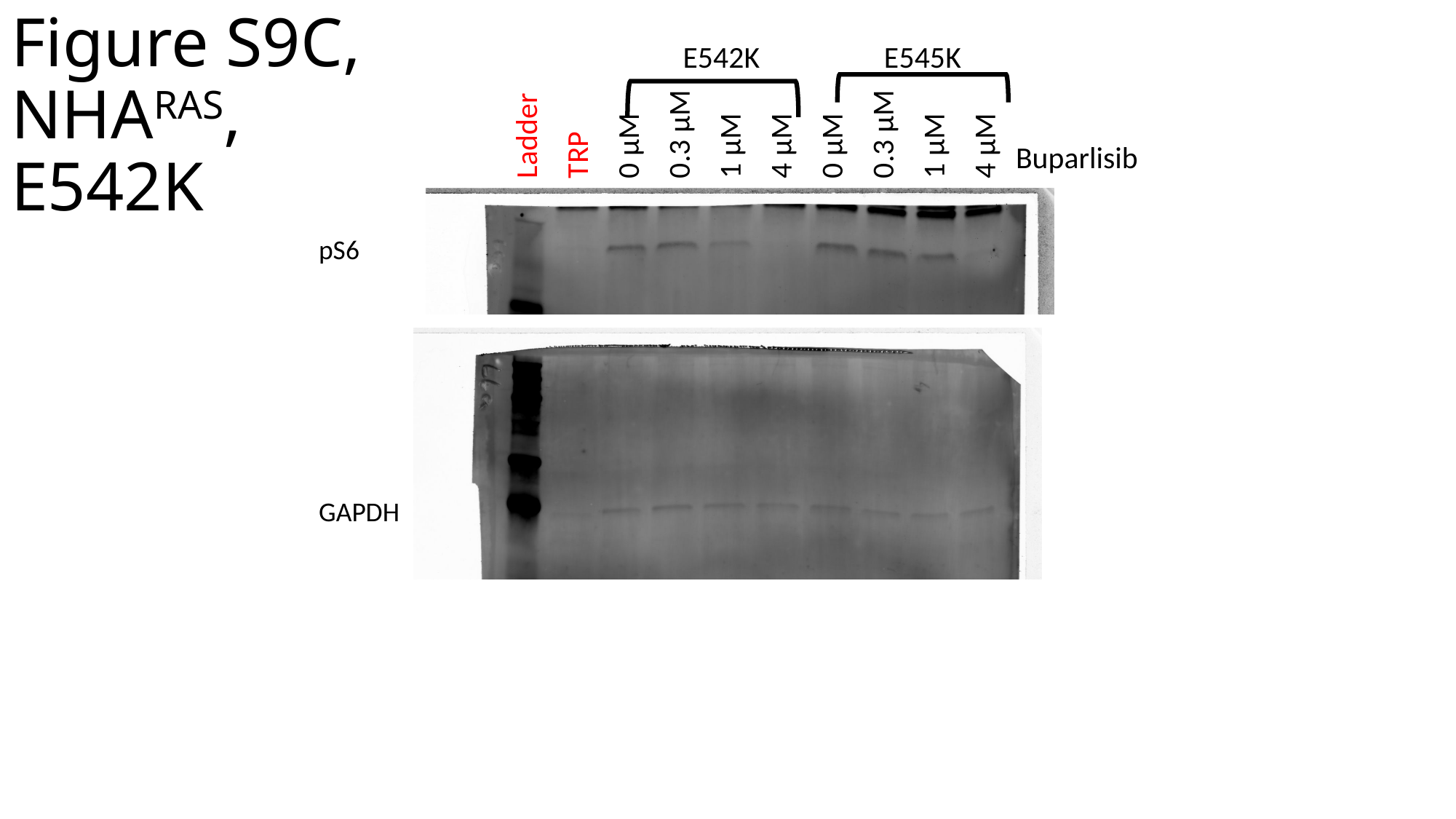

# Figure S9C, NHARAS, E542K
 E542K E545K
Ladder
TRP
0 µM
0.3 µM
1 µM
4 µM
0 µM
0.3 µM
1 µM
4 µM
Buparlisib
pS6
GAPDH

## Slide 46
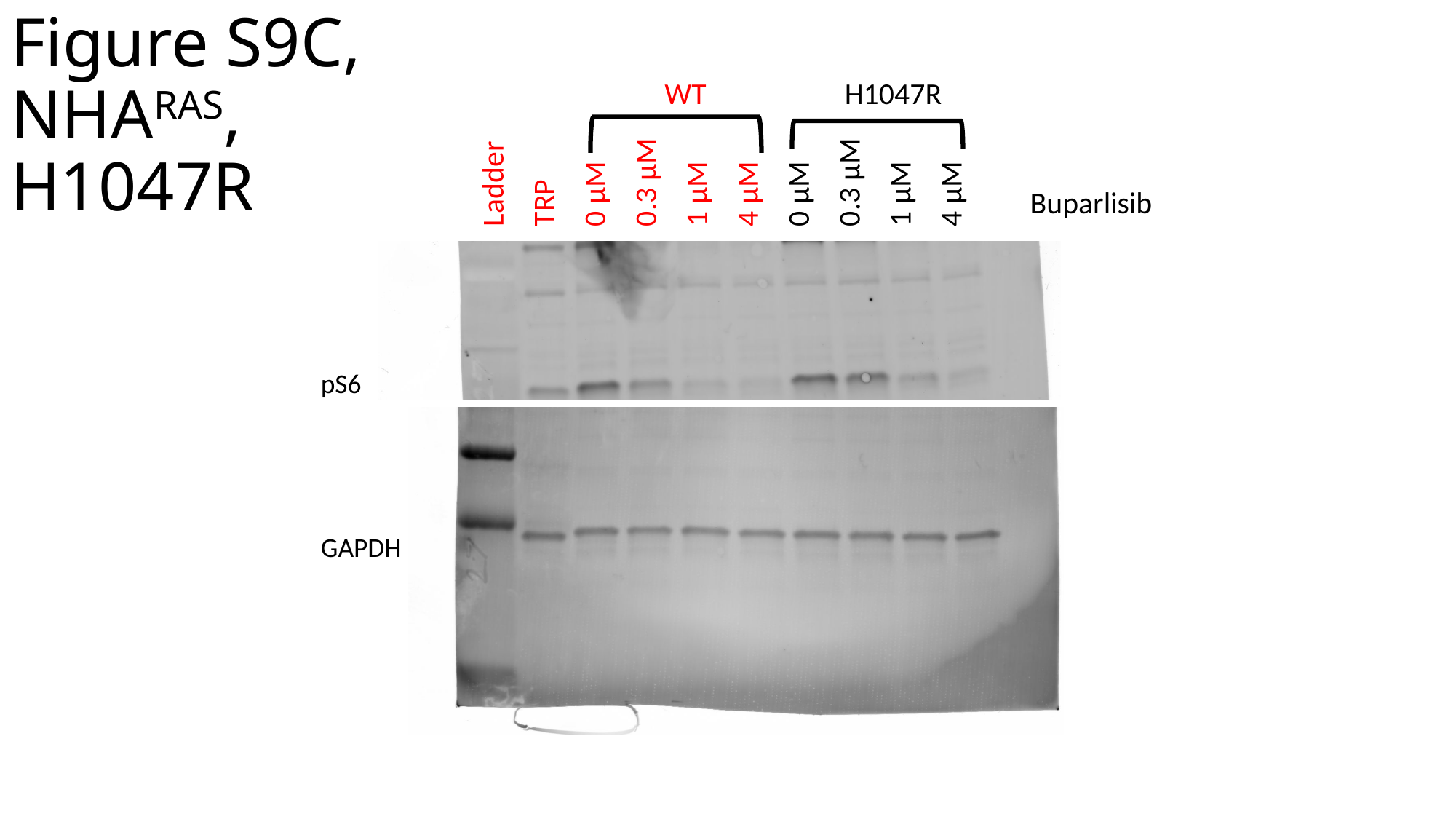

# Figure S9C, NHARAS, H1047R
 WT H1047R
Ladder
TRP
0 µM
0.3 µM
1 µM
4 µM
0 µM
0.3 µM
1 µM
4 µM
Buparlisib
pS6
GAPDH

## Slide 47
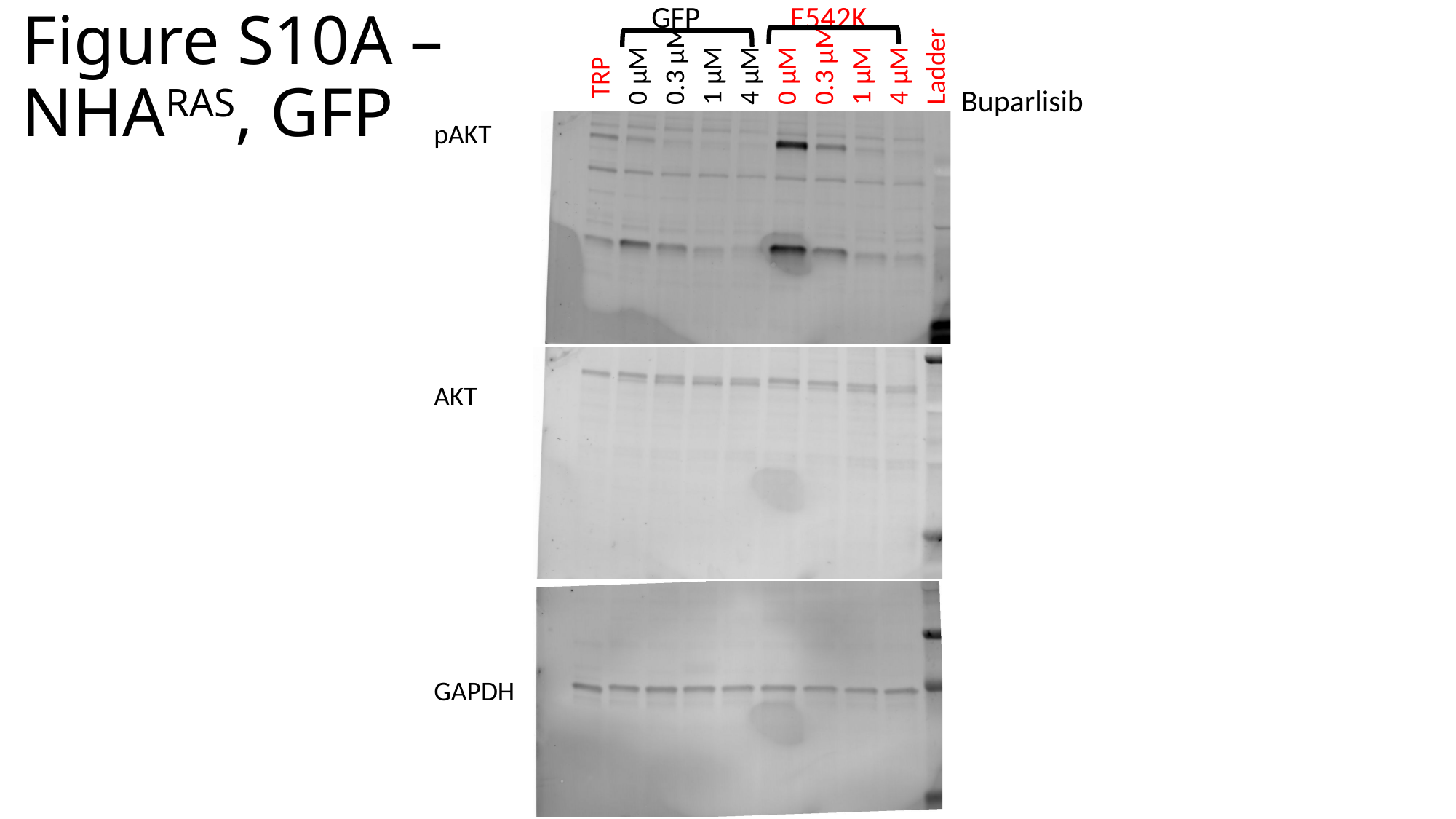

# Figure S10A – NHARAS, GFP
 TRP
0 µM
0.3 µM
1 µM
4 µM
0 µM
0.3 µM
1 µM
4 µM
Ladder
 GFP E542K
Buparlisib
pAKT
AKT
GAPDH

## Slide 48
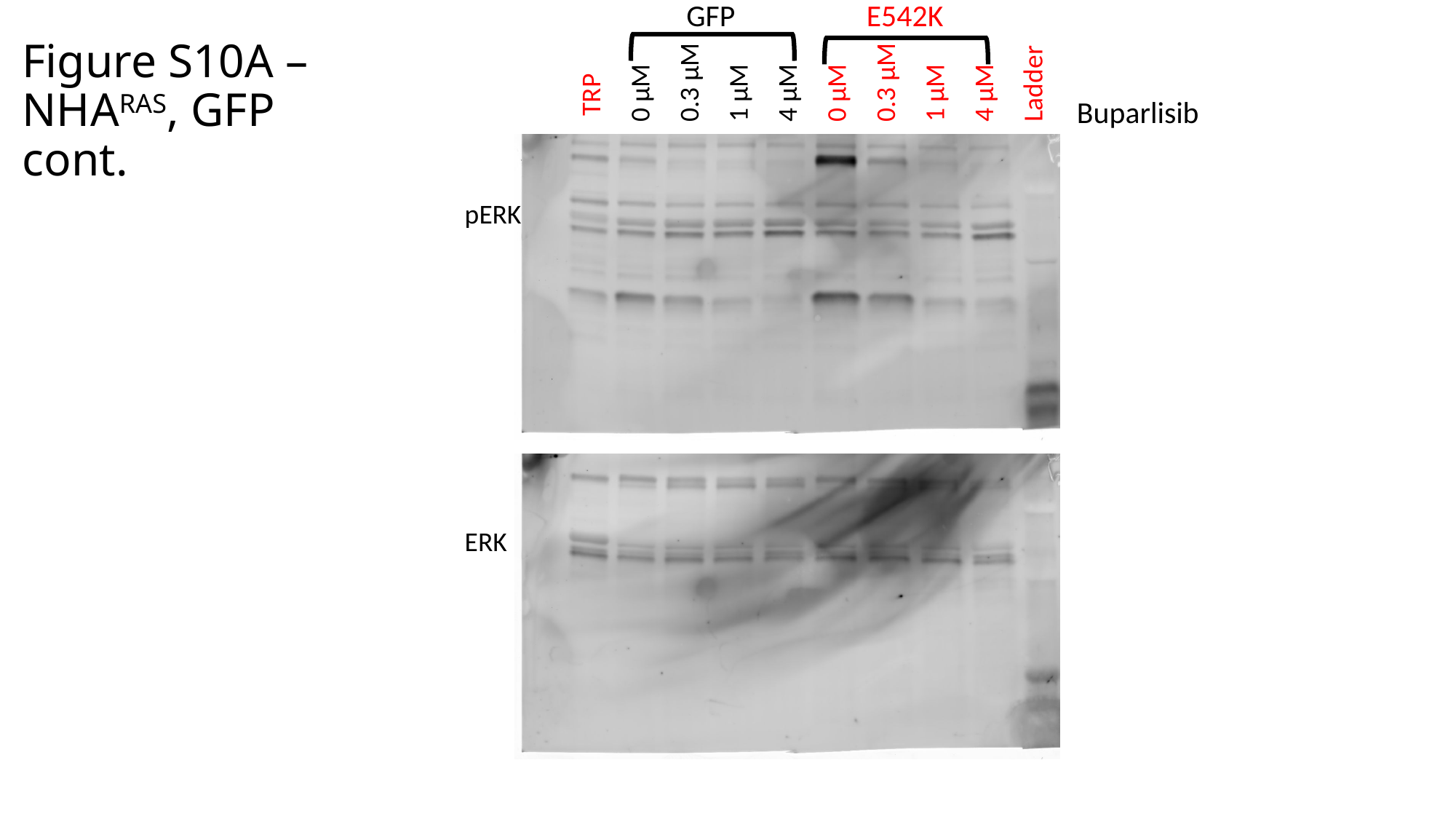

TRP
0 µM
0.3 µM
1 µM
4 µM
0 µM
0.3 µM
1 µM
4 µM
Ladder
 GFP E542K
# Figure S10A – NHARAS, GFP cont.
Buparlisib
pERK
ERK

## Slide 49
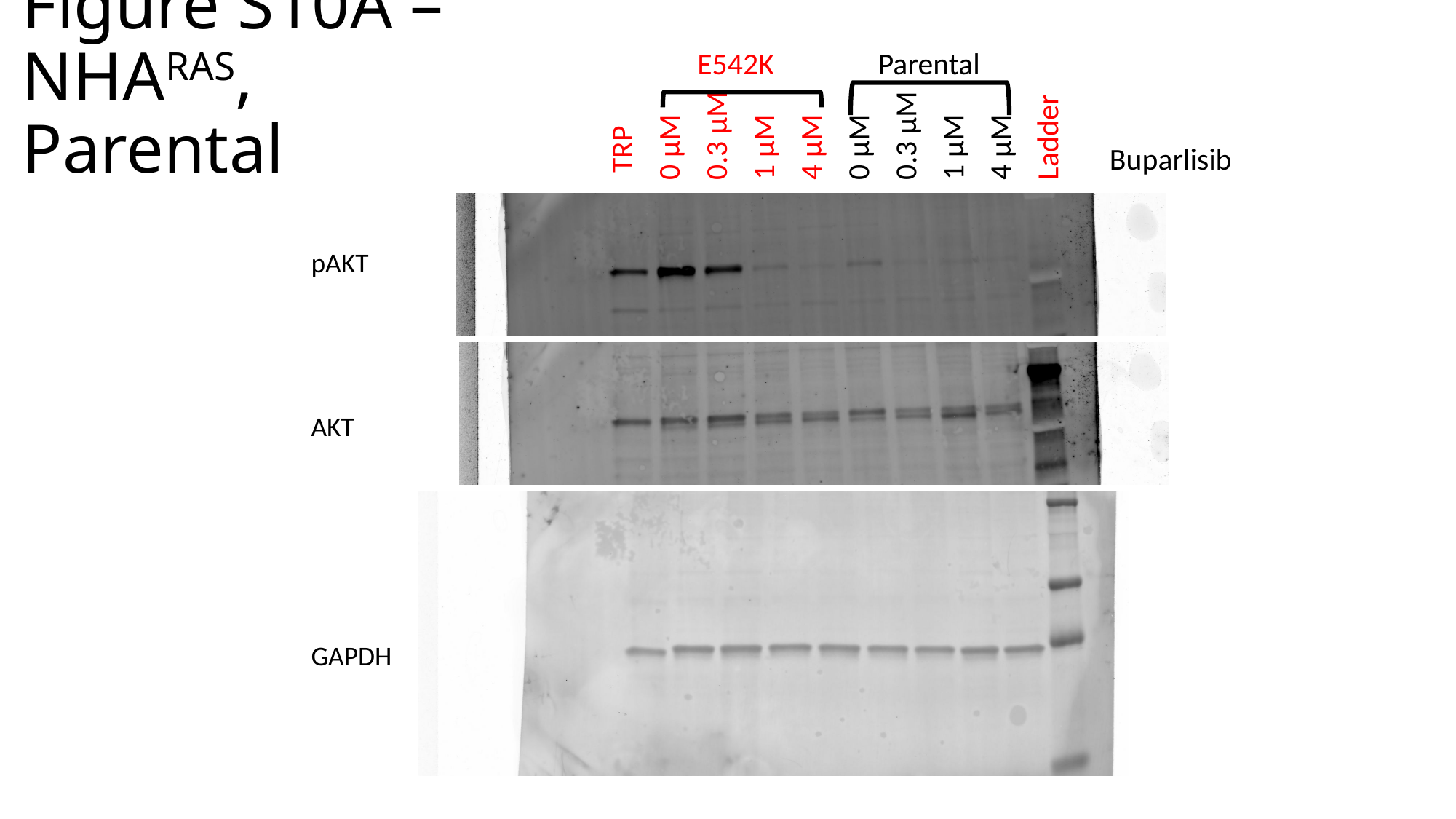

# Figure S10A – NHARAS, Parental
 E542K Parental
 TRP
0 µM
0.3 µM
1 µM
4 µM
0 µM
0.3 µM
1 µM
4 µM
Ladder
Buparlisib
pAKT
AKT
GAPDH

## Slide 50
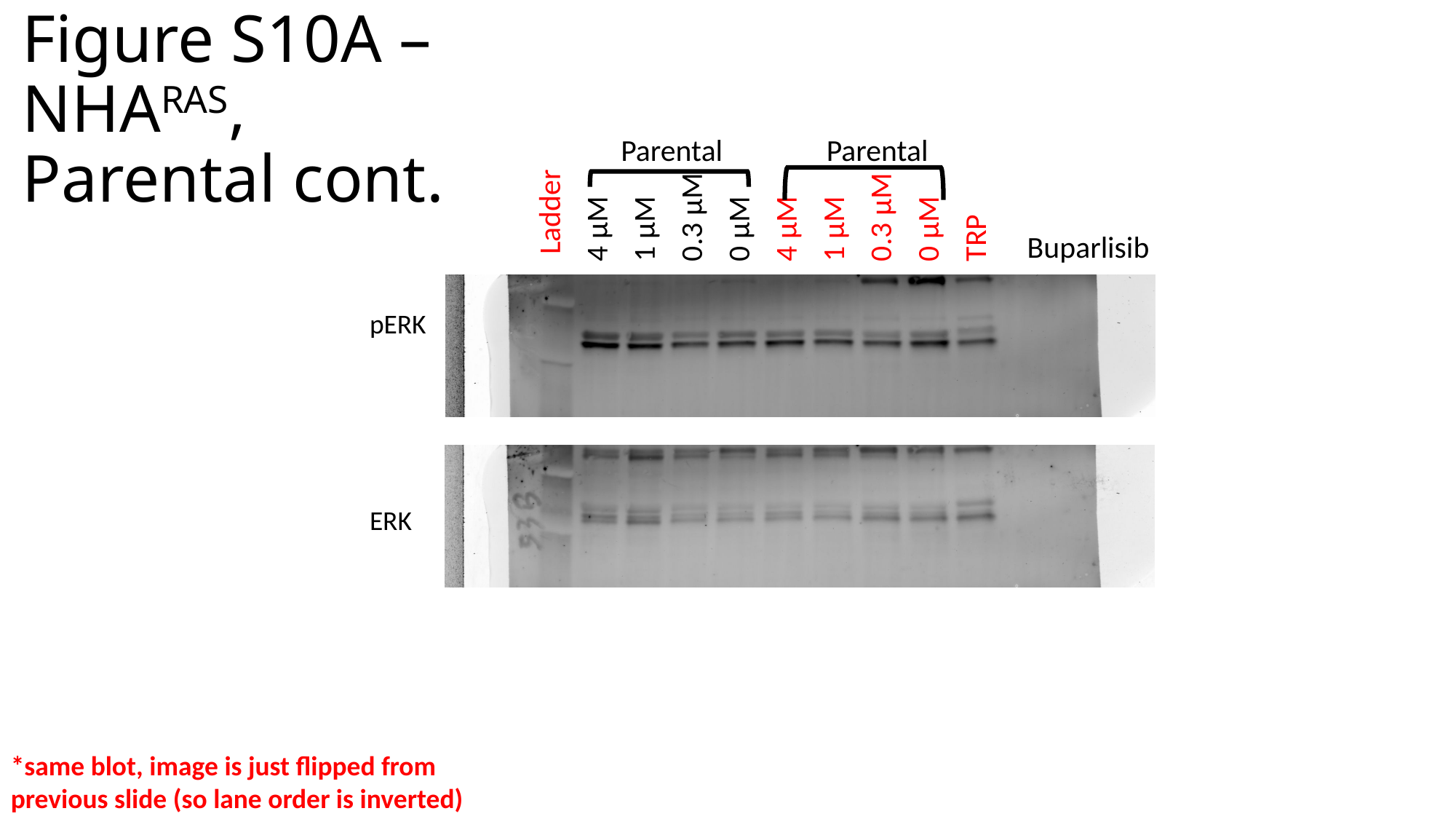

# Figure S10A – NHARAS, Parental cont.
 Parental Parental
 Ladder
4 µM
1 µM
0.3 µM
0 µM
4 µM
1 µM
0.3 µM
0 µM
TRP
Buparlisib
pERK
ERK
*same blot, image is just flipped from previous slide (so lane order is inverted)

## Slide 51
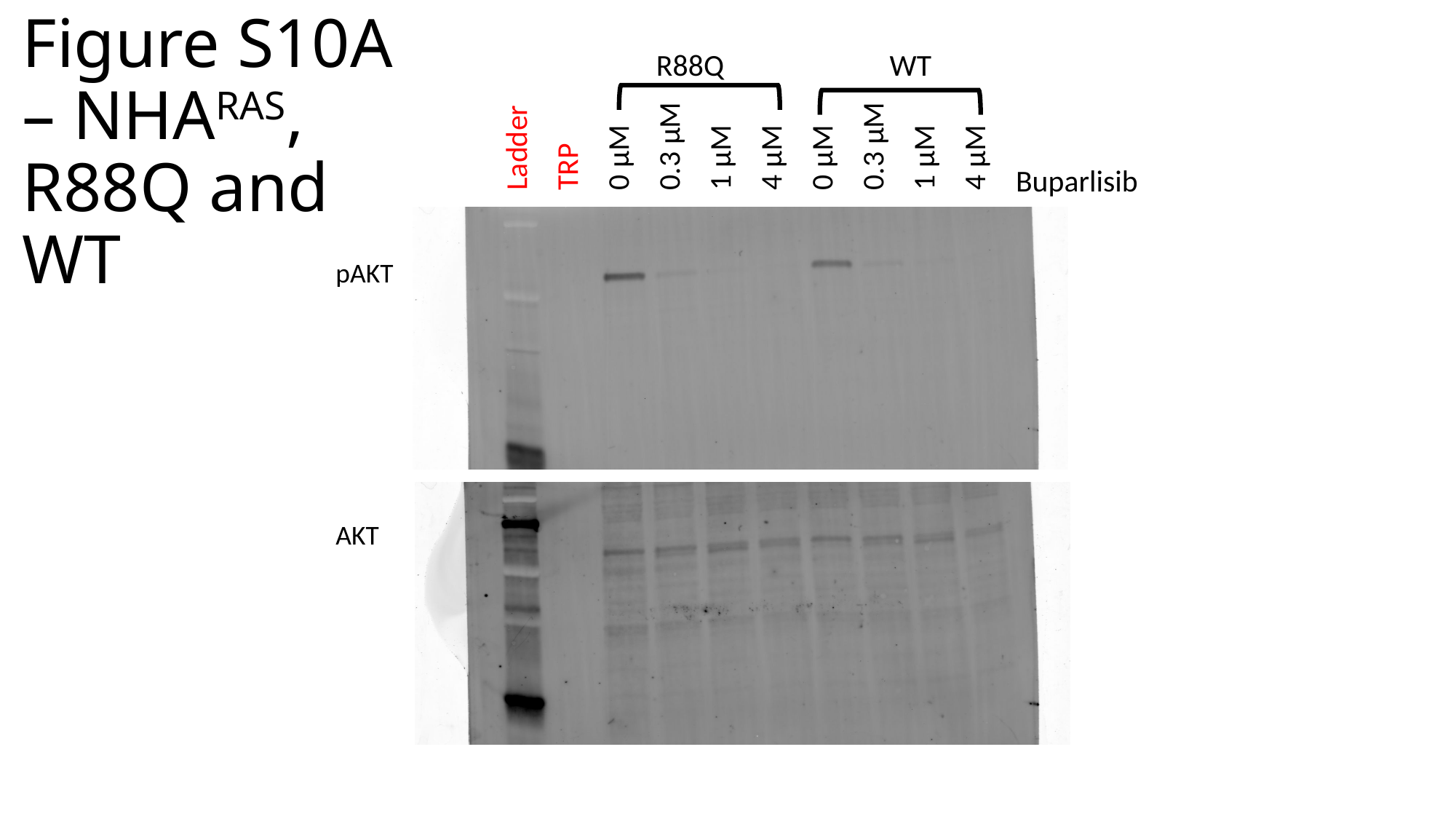

# Figure S10A – NHARAS, R88Q and WT
 R88Q WT
Ladder
TRP
0 µM
0.3 µM
1 µM
4 µM
0 µM
0.3 µM
1 µM
4 µM
Buparlisib
pAKT
AKT

## Slide 52
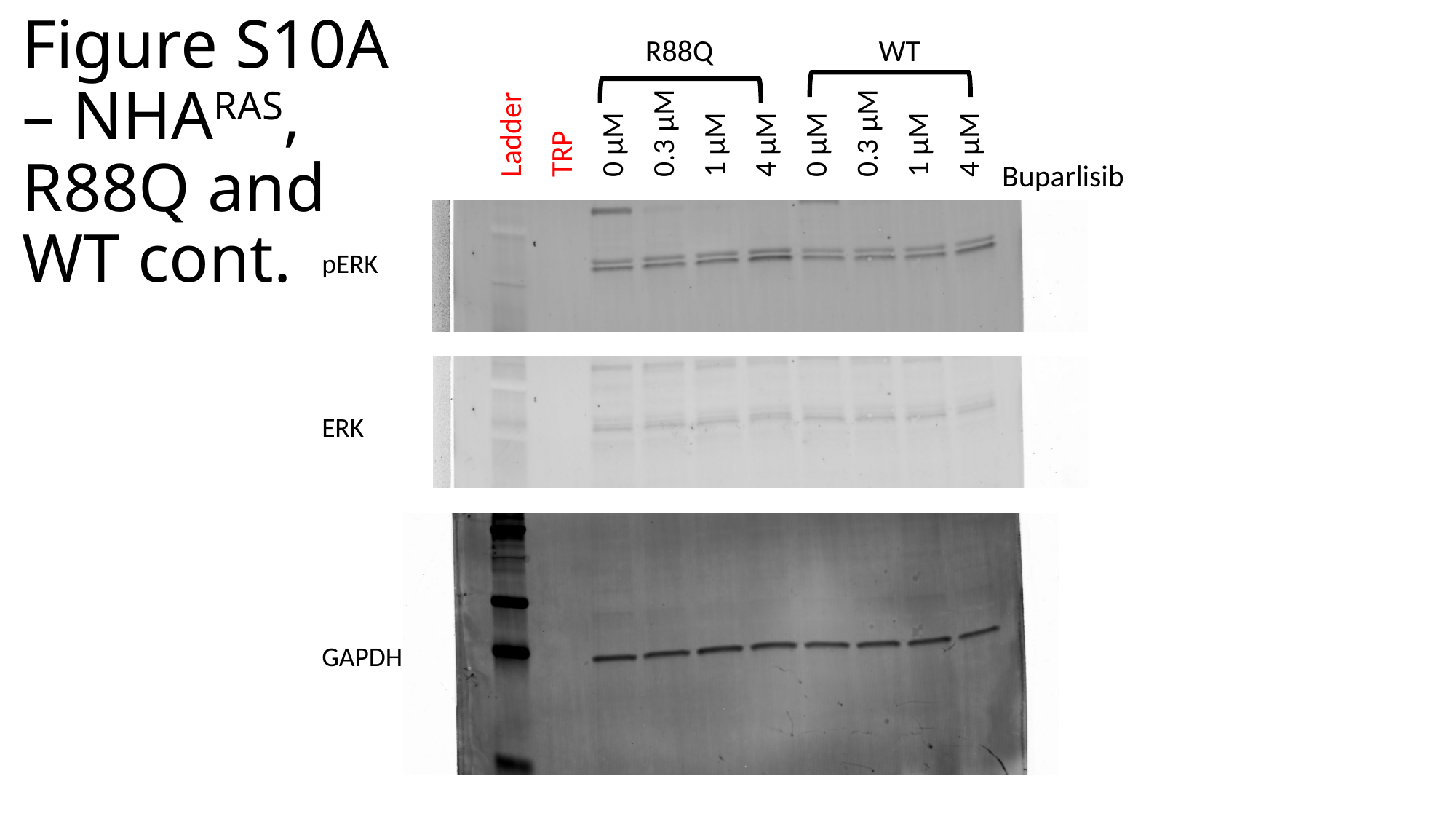

# Figure S10A – NHARAS, R88Q and WT cont.
 R88Q WT
Ladder
TRP
0 µM
0.3 µM
1 µM
4 µM
0 µM
0.3 µM
1 µM
4 µM
Buparlisib
pERK
ERK
GAPDH

## Slide 53
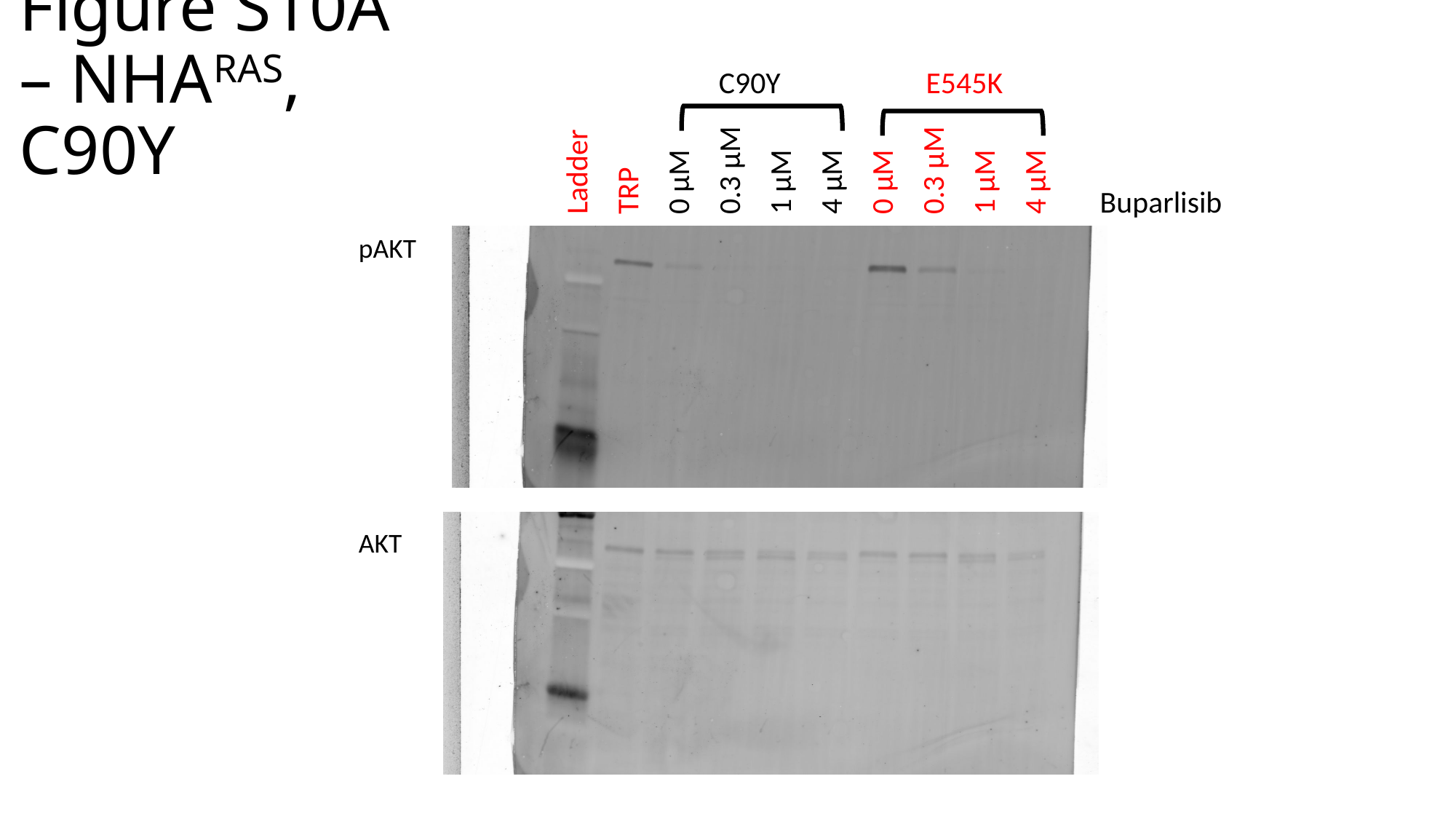

# Figure S10A – NHARAS, C90Y
 C90Y E545K
Ladder
TRP
0 µM
0.3 µM
1 µM
4 µM
0 µM
0.3 µM
1 µM
4 µM
Buparlisib
pAKT
AKT

## Slide 54
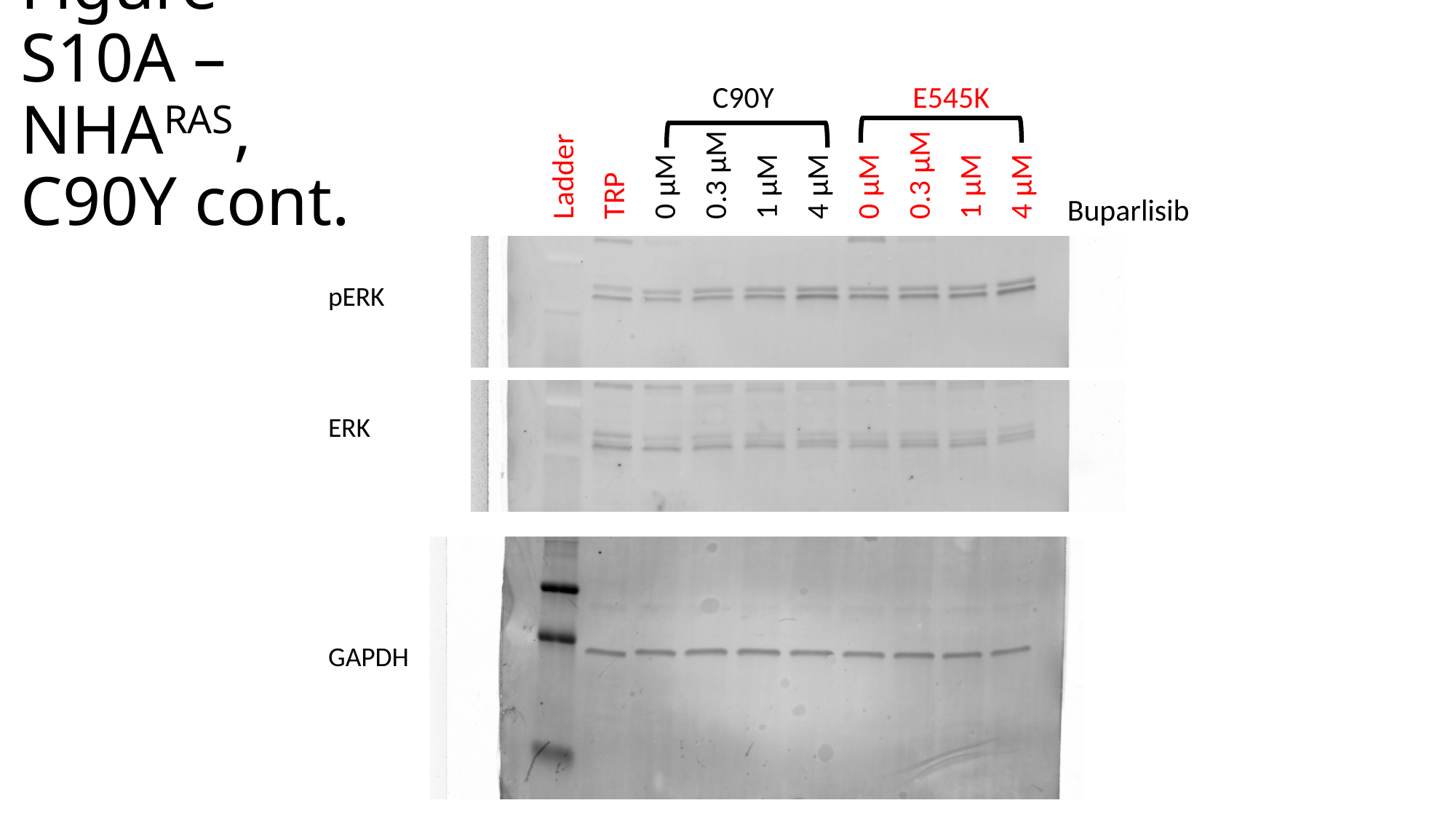

# Figure S10A – NHARAS, C90Y cont.
 C90Y E545K
Ladder
TRP
0 µM
0.3 µM
1 µM
4 µM
0 µM
0.3 µM
1 µM
4 µM
Buparlisib
pERK
ERK
GAPDH

## Slide 55
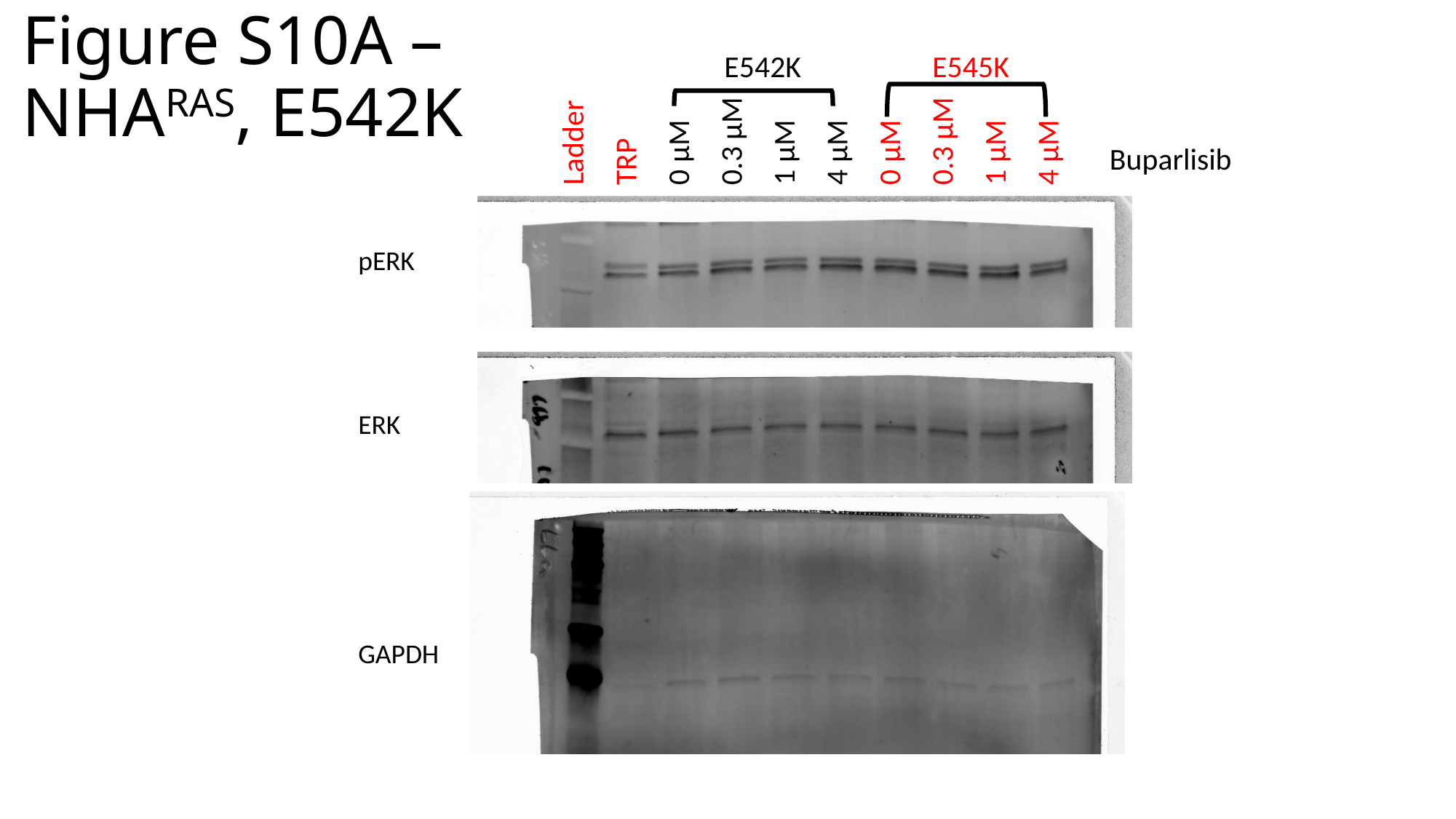

# Figure S10A – NHARAS, E542K
 E542K E545K
Ladder
TRP
0 µM
0.3 µM
1 µM
4 µM
0 µM
0.3 µM
1 µM
4 µM
Buparlisib
pERK
ERK
GAPDH

## Slide 56
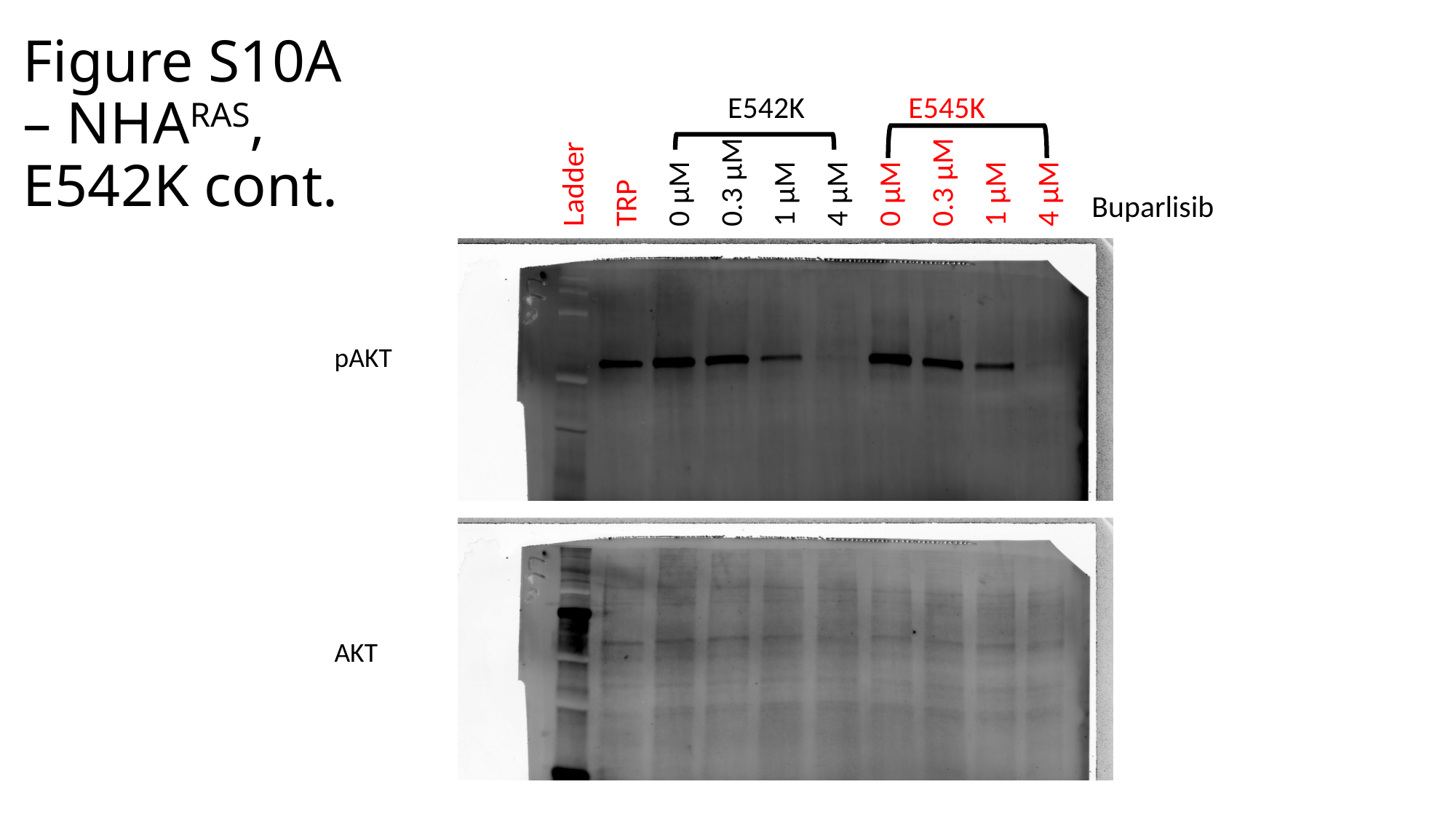

# Figure S10A – NHARAS, E542K cont.
 E542K E545K
Ladder
TRP
0 µM
0.3 µM
1 µM
4 µM
0 µM
0.3 µM
1 µM
4 µM
Buparlisib
pAKT
AKT

## Slide 57
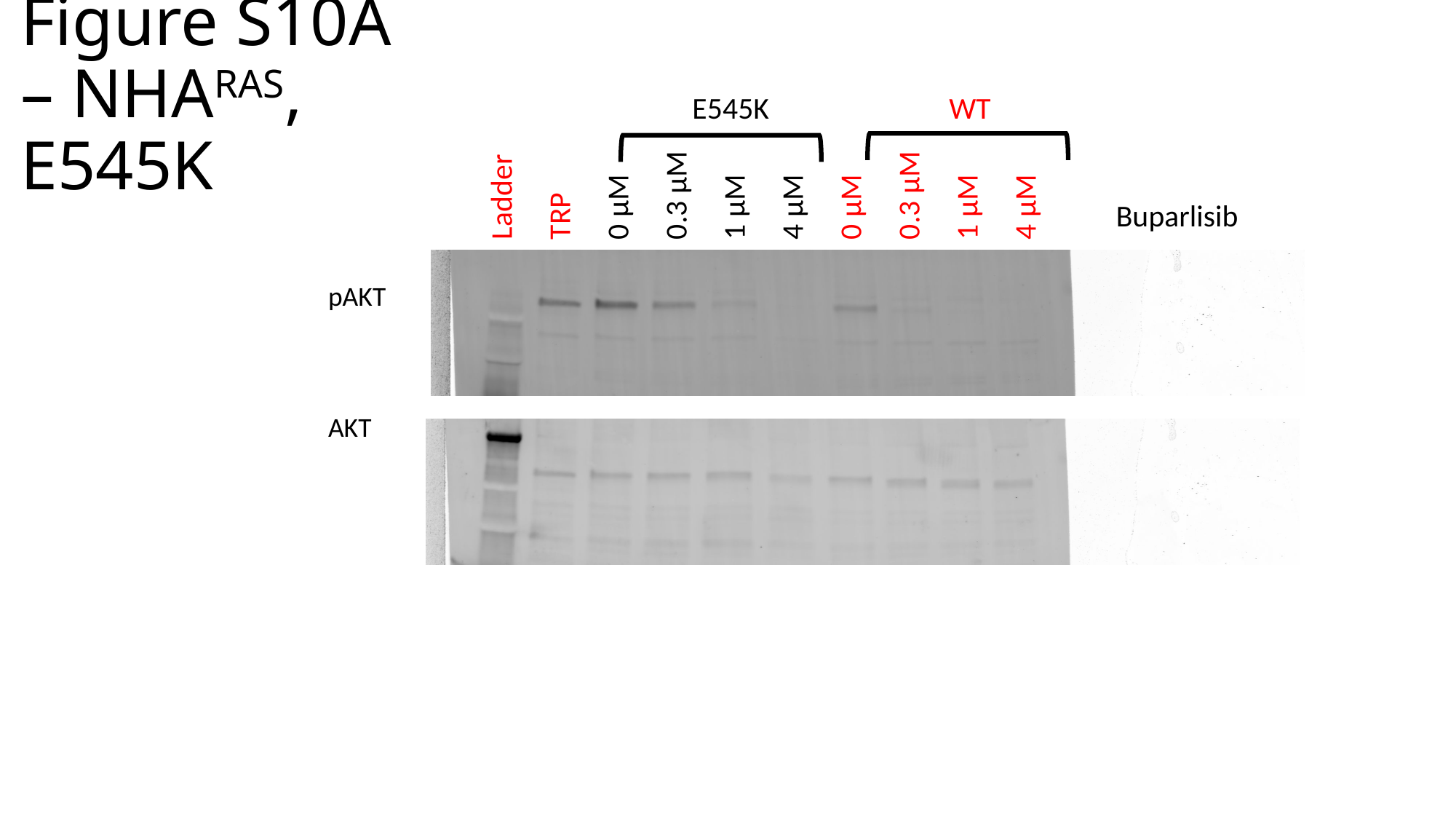

# Figure S10A – NHARAS, E545K
 E545K WT
Ladder
TRP
0 µM
0.3 µM
1 µM
4 µM
0 µM
0.3 µM
1 µM
4 µM
Buparlisib
pAKT
AKT

## Slide 58
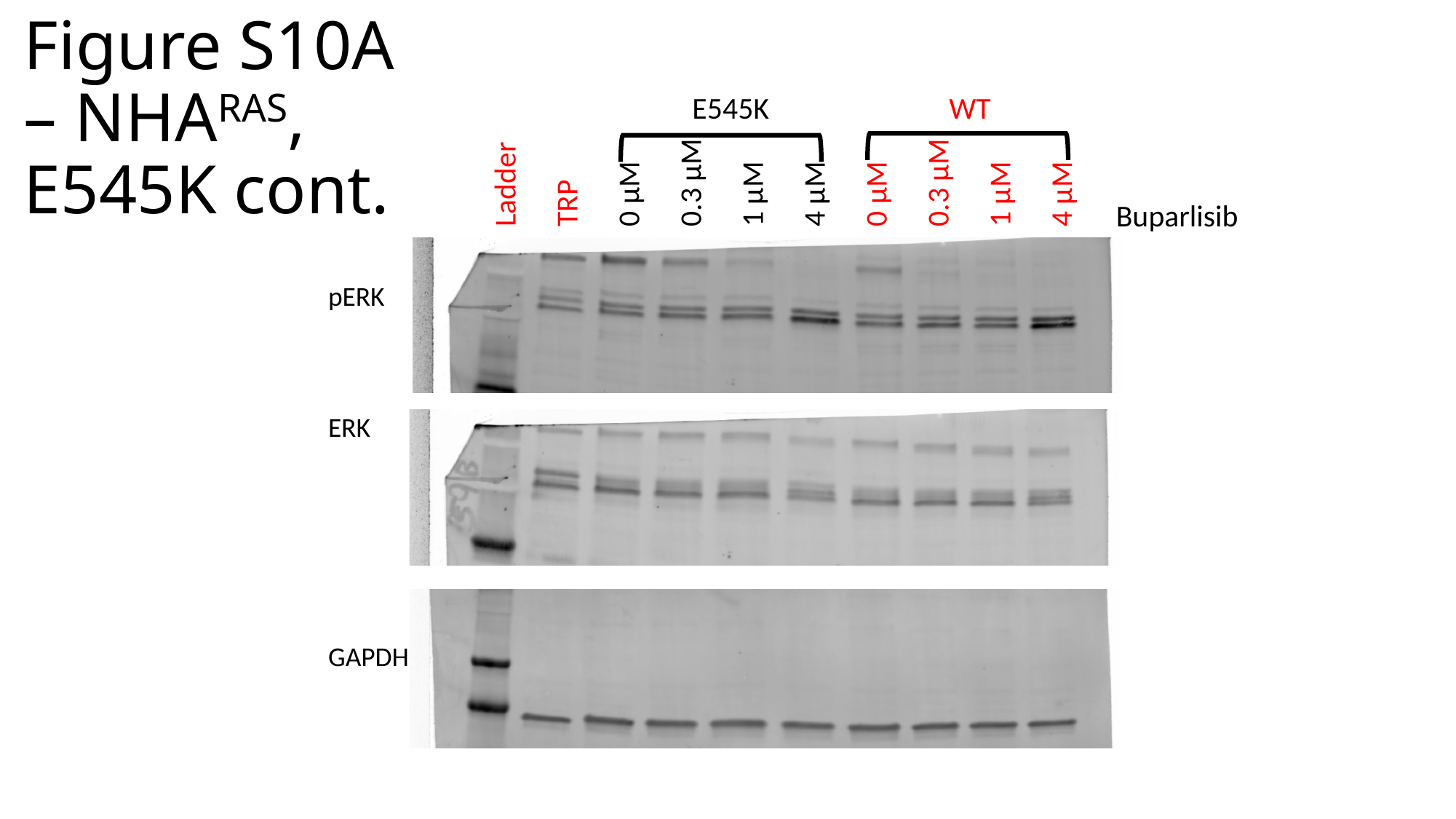

# Figure S10A – NHARAS, E545K cont.
 E545K WT
Ladder
TRP
0 µM
0.3 µM
1 µM
4 µM
0 µM
0.3 µM
1 µM
4 µM
Buparlisib
pERK
ERK
GAPDH

## Slide 59
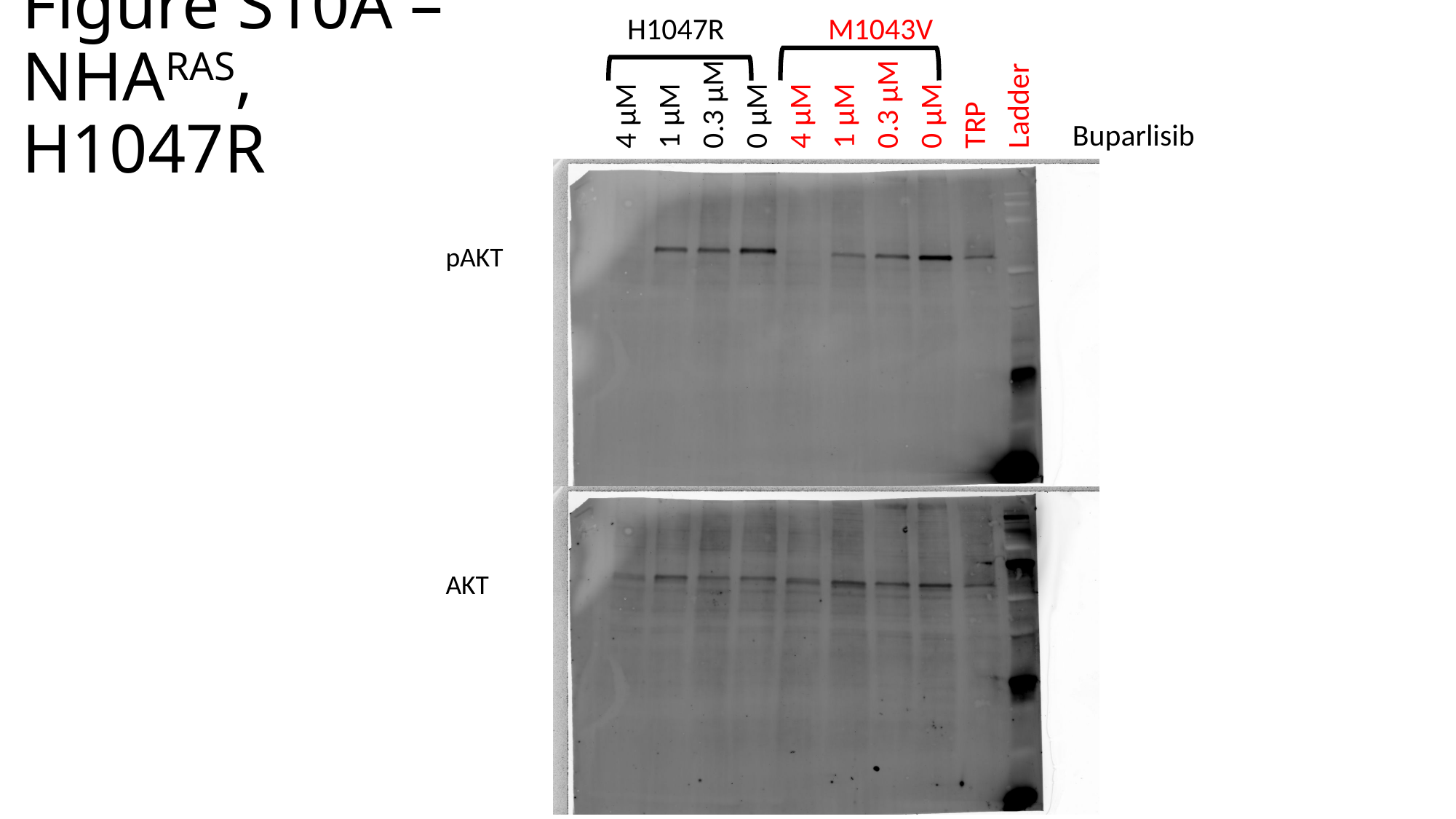

# Figure S10A – NHARAS, H1047R
 H1047R M1043V
4 µM
1 µM
0.3 µM
0 µM
4 µM
1 µM
0.3 µM
0 µM
TRP
Ladder
Buparlisib
pAKT
AKT

## Slide 60
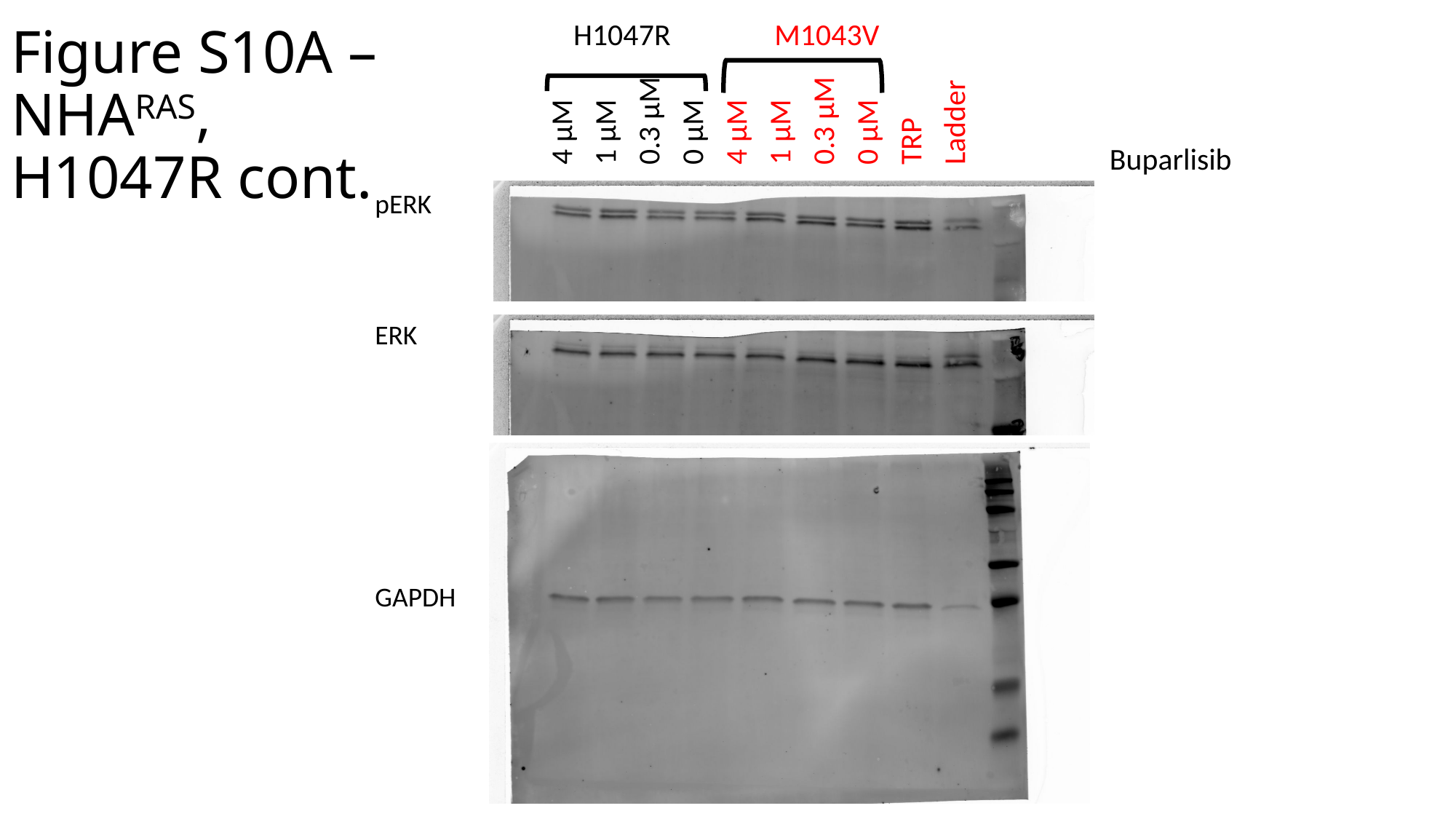

# Figure S10A – NHARAS, H1047R cont.
 H1047R M1043V
4 µM
1 µM
0.3 µM
0 µM
4 µM
1 µM
0.3 µM
0 µM
TRP
Ladder
Buparlisib
pERK
ERK
GAPDH

## Slide 61
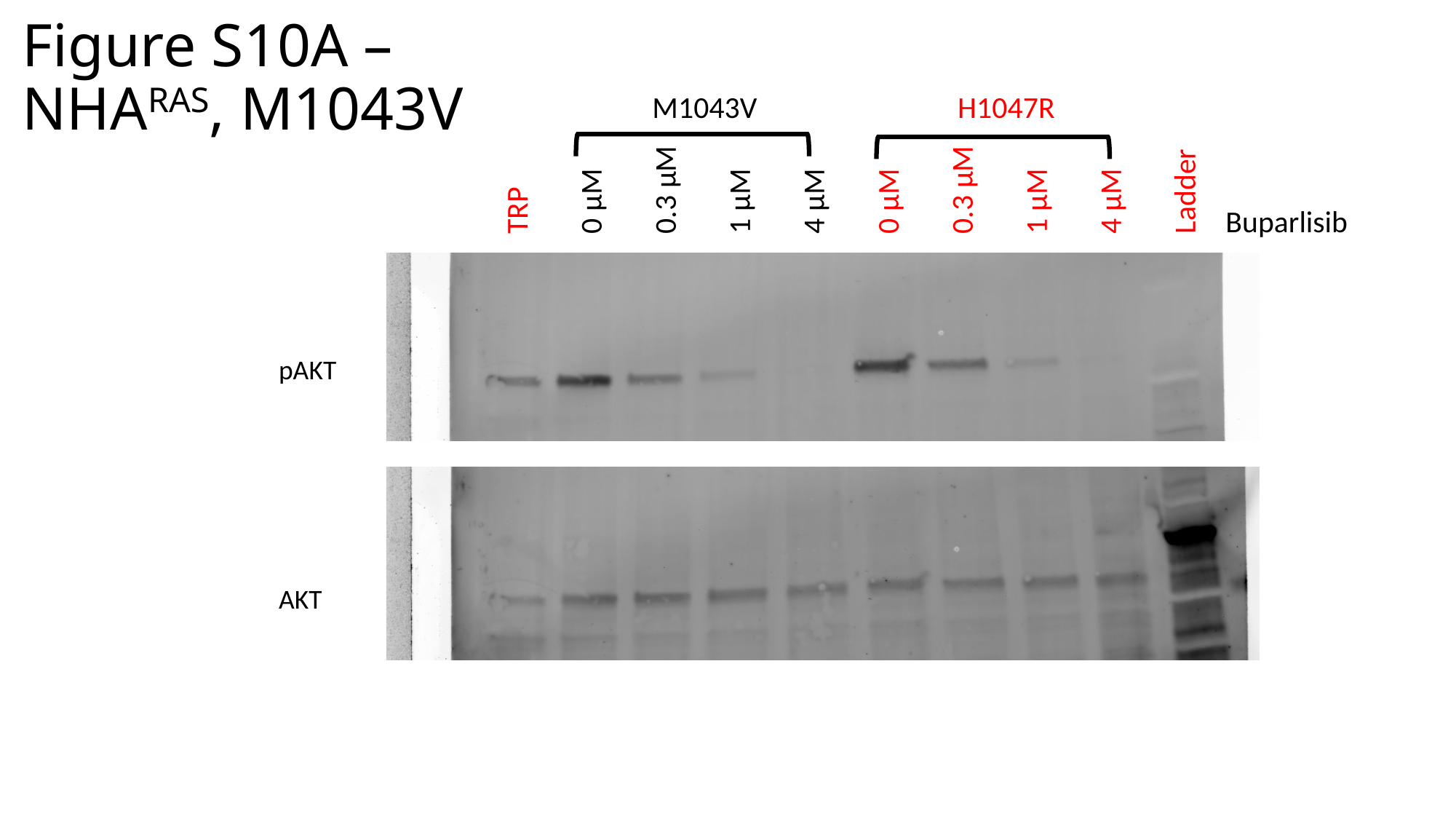

# Figure S10A – NHARAS, M1043V
 M1043V H1047R
TRP
0 µM
0.3 µM
1 µM
4 µM
0 µM
0.3 µM
1 µM
4 µM
Ladder
Buparlisib
pAKT
AKT

## Slide 62
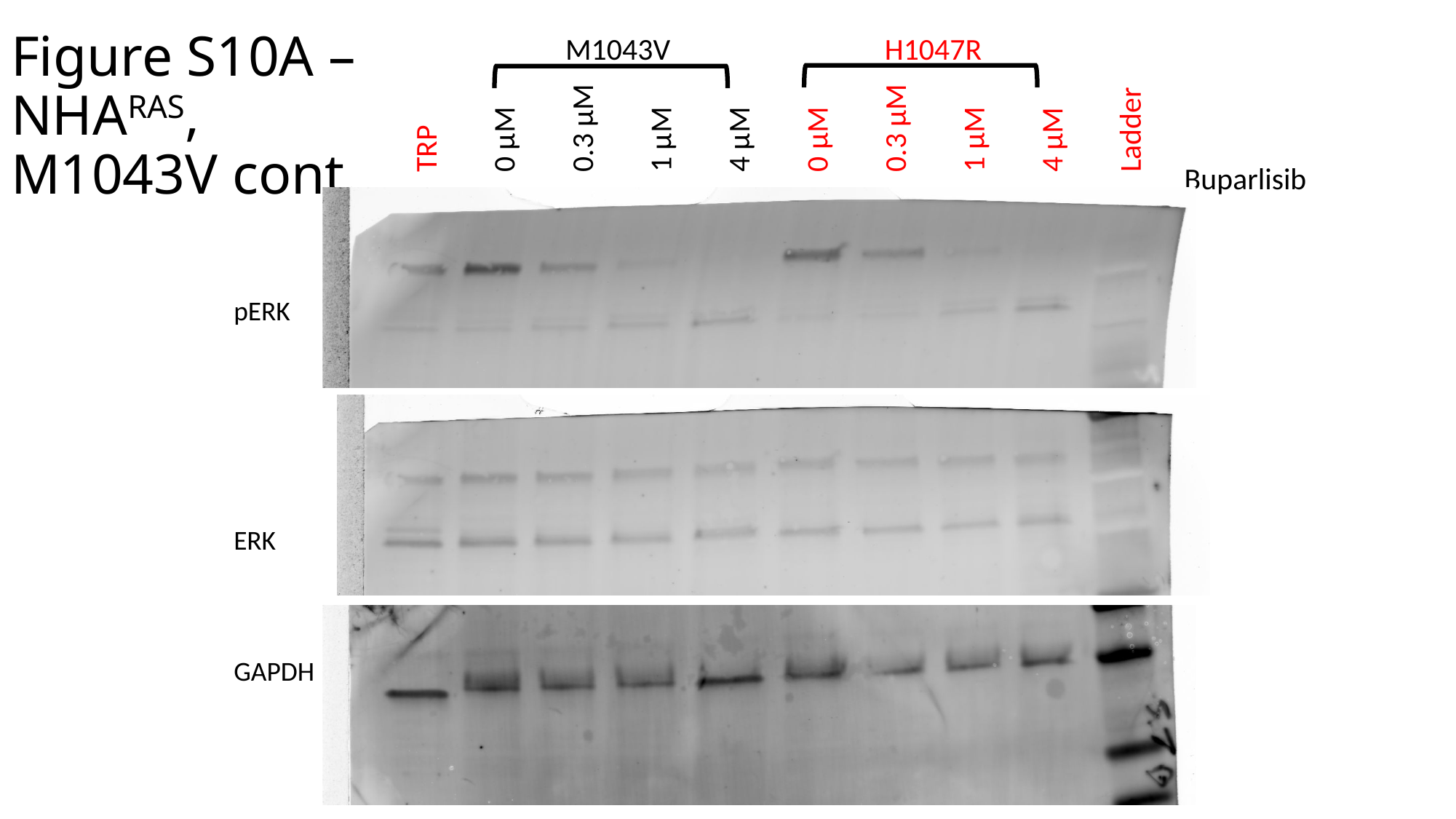

# Figure S10A – NHARAS, M1043V cont.
 M1043V H1047R
TRP
0 µM
0.3 µM
1 µM
4 µM
0 µM
0.3 µM
1 µM
4 µM
Ladder
Buparlisib
pERK
ERK
GAPDH

## Slide 63
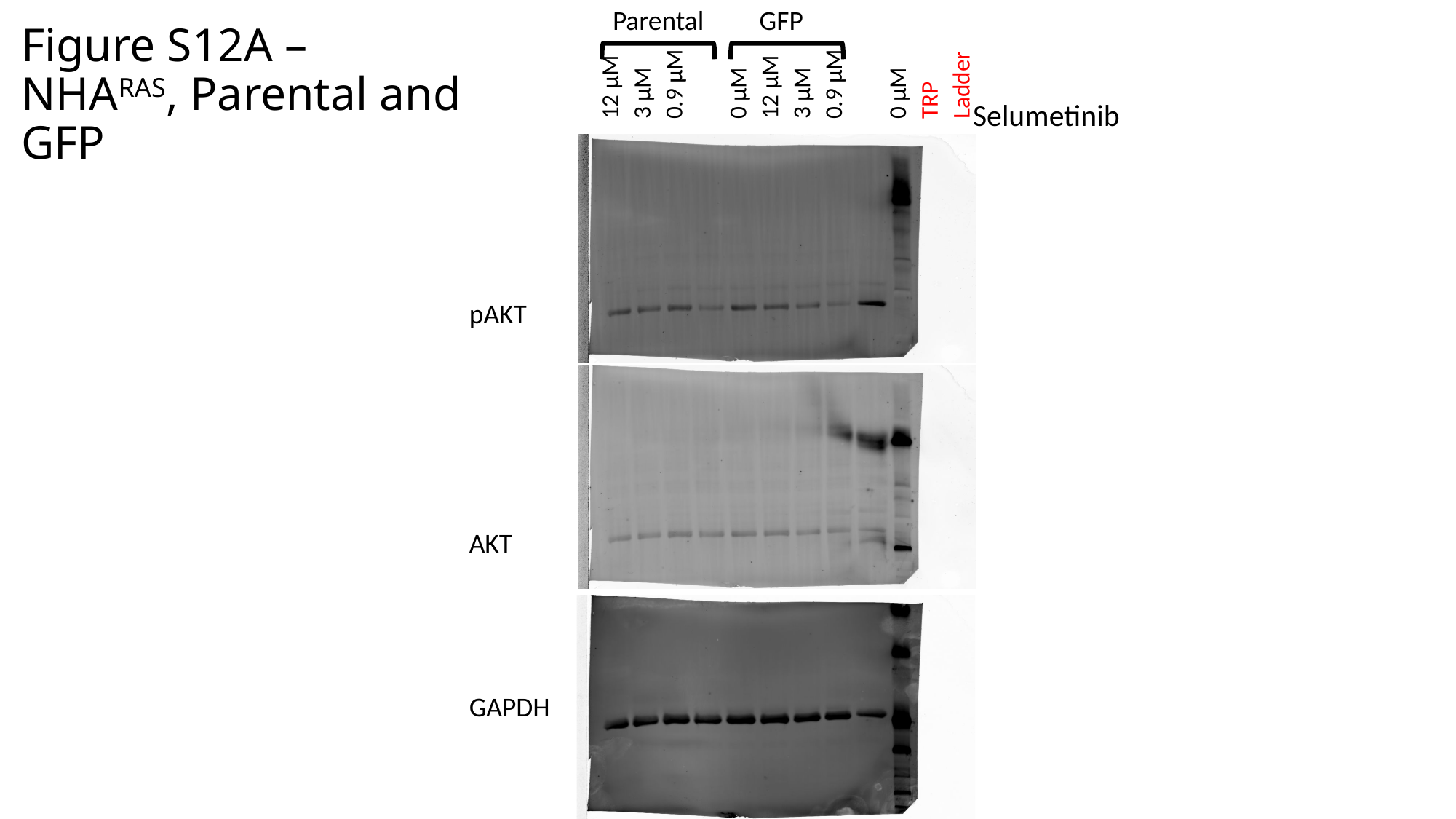

Parental GFP
12 µM
3 µM
0.9 µM
0 µM
12 µM
3 µM
0.9 µM
0 µM
TRP
Ladder
# Figure S12A – NHARAS, Parental and GFP
Selumetinib
pAKT
AKT
GAPDH

## Slide 64
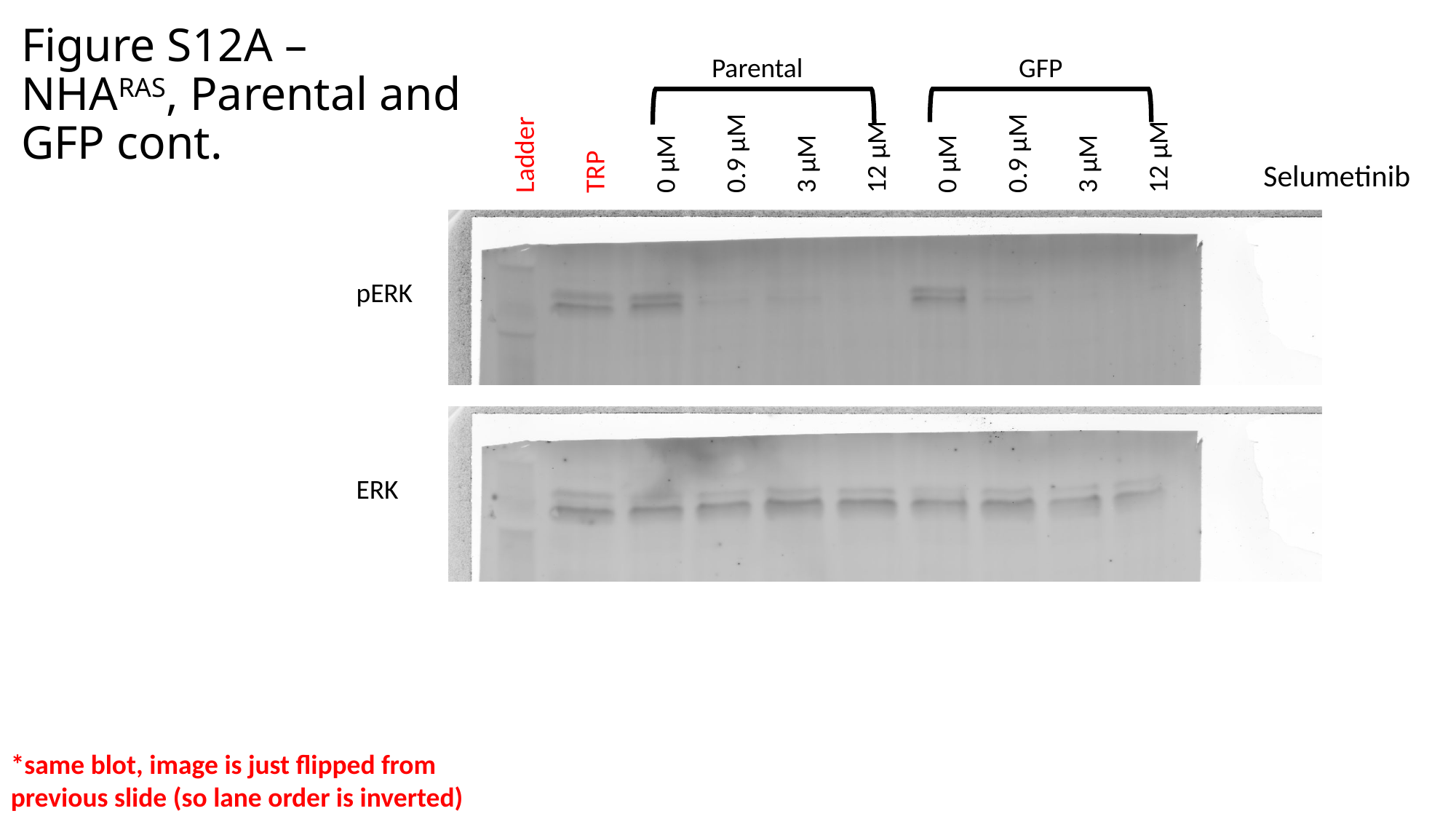

# Figure S12A – NHARAS, Parental and GFP cont.
Parental GFP
Ladder
TRP
0 µM
0.9 µM
3 µM
12 µM
0 µM
0.9 µM
3 µM
12 µM
Selumetinib
pERK
ERK
*same blot, image is just flipped from previous slide (so lane order is inverted)

## Slide 65
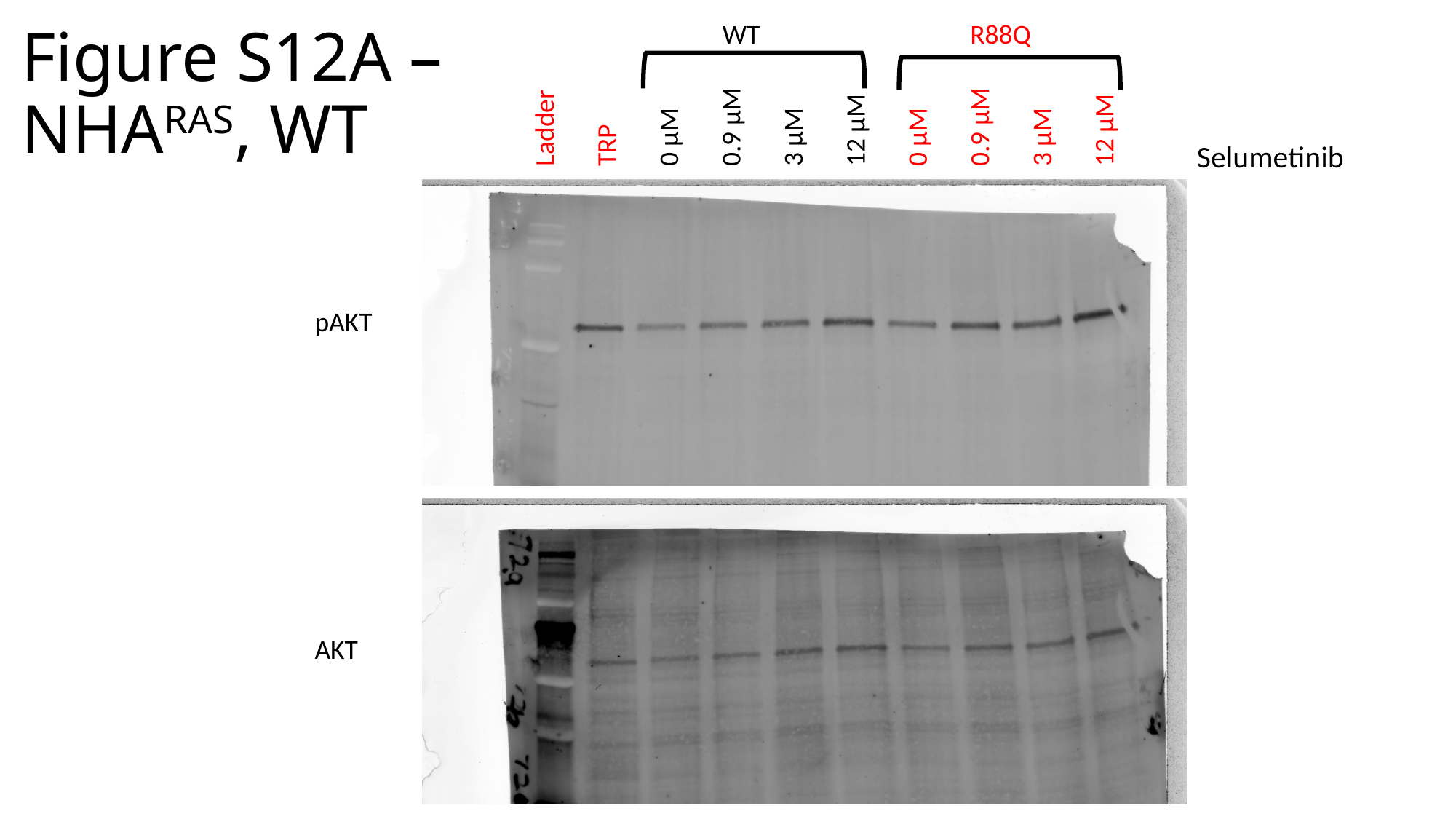

WT R88Q
# Figure S12A – NHARAS, WT
Ladder
TRP
0 µM
0.9 µM
3 µM
12 µM
0 µM
0.9 µM
3 µM
12 µM
Selumetinib
pAKT
AKT

## Slide 66
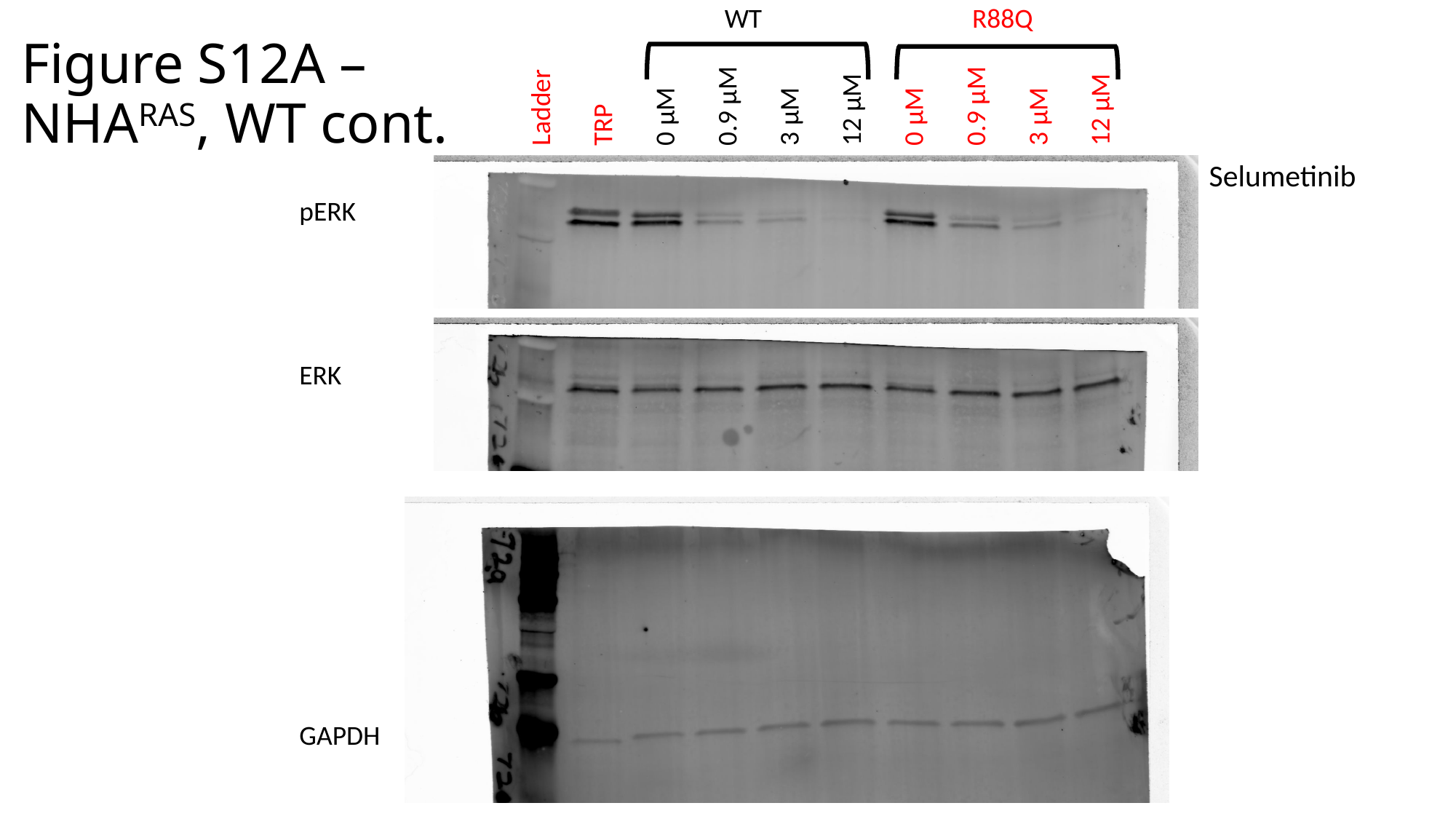

WT R88Q
Ladder
TRP
0 µM
0.9 µM
3 µM
12 µM
0 µM
0.9 µM
3 µM
12 µM
# Figure S12A – NHARAS, WT cont.
Selumetinib
pERK
ERK
GAPDH

## Slide 67
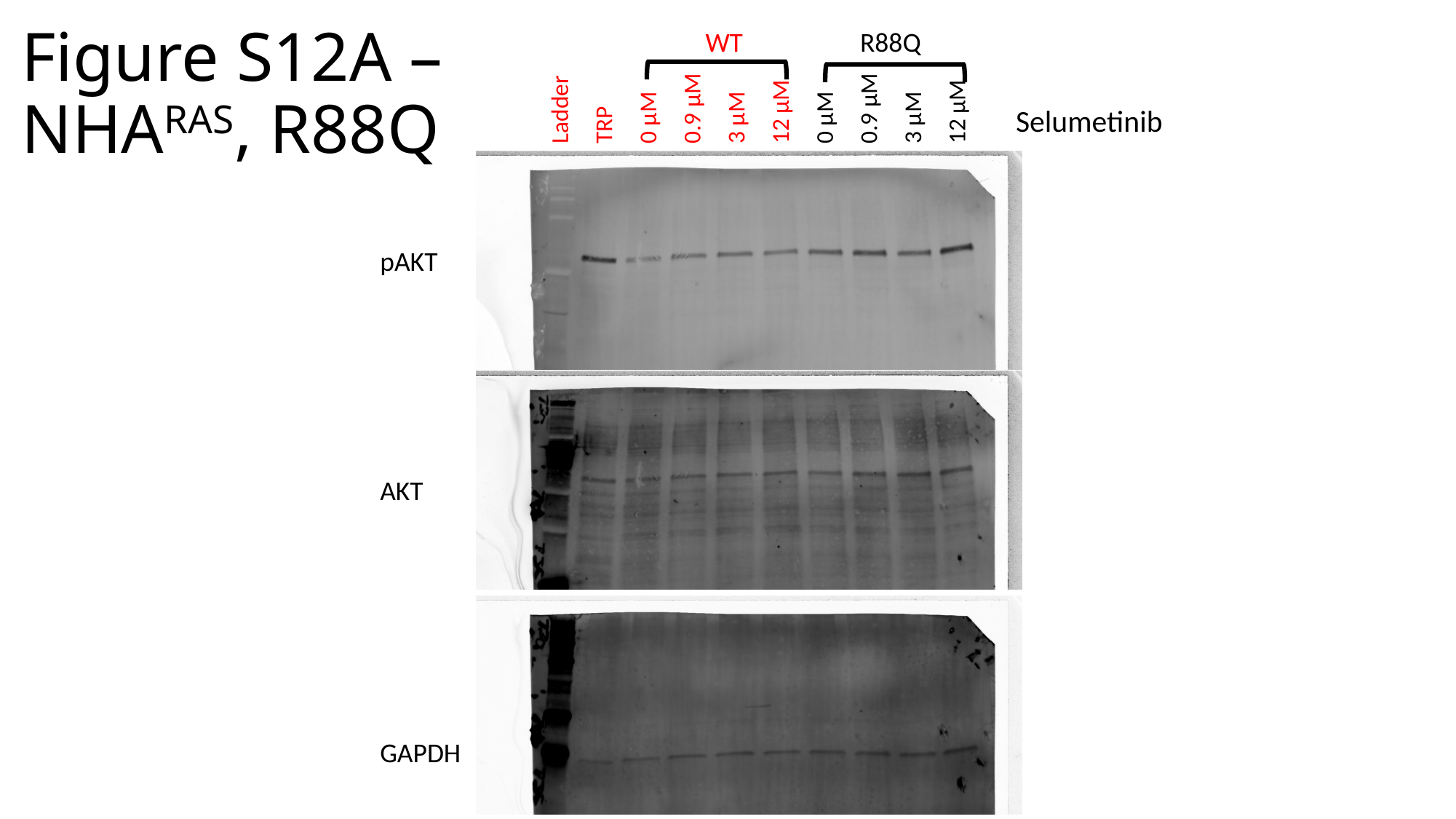

Ladder
TRP
0 µM
0.9 µM
3 µM
12 µM
0 µM
0.9 µM
3 µM
12 µM
# Figure S12A – NHARAS, R88Q
WT R88Q
Selumetinib
pAKT
AKT
GAPDH

## Slide 68
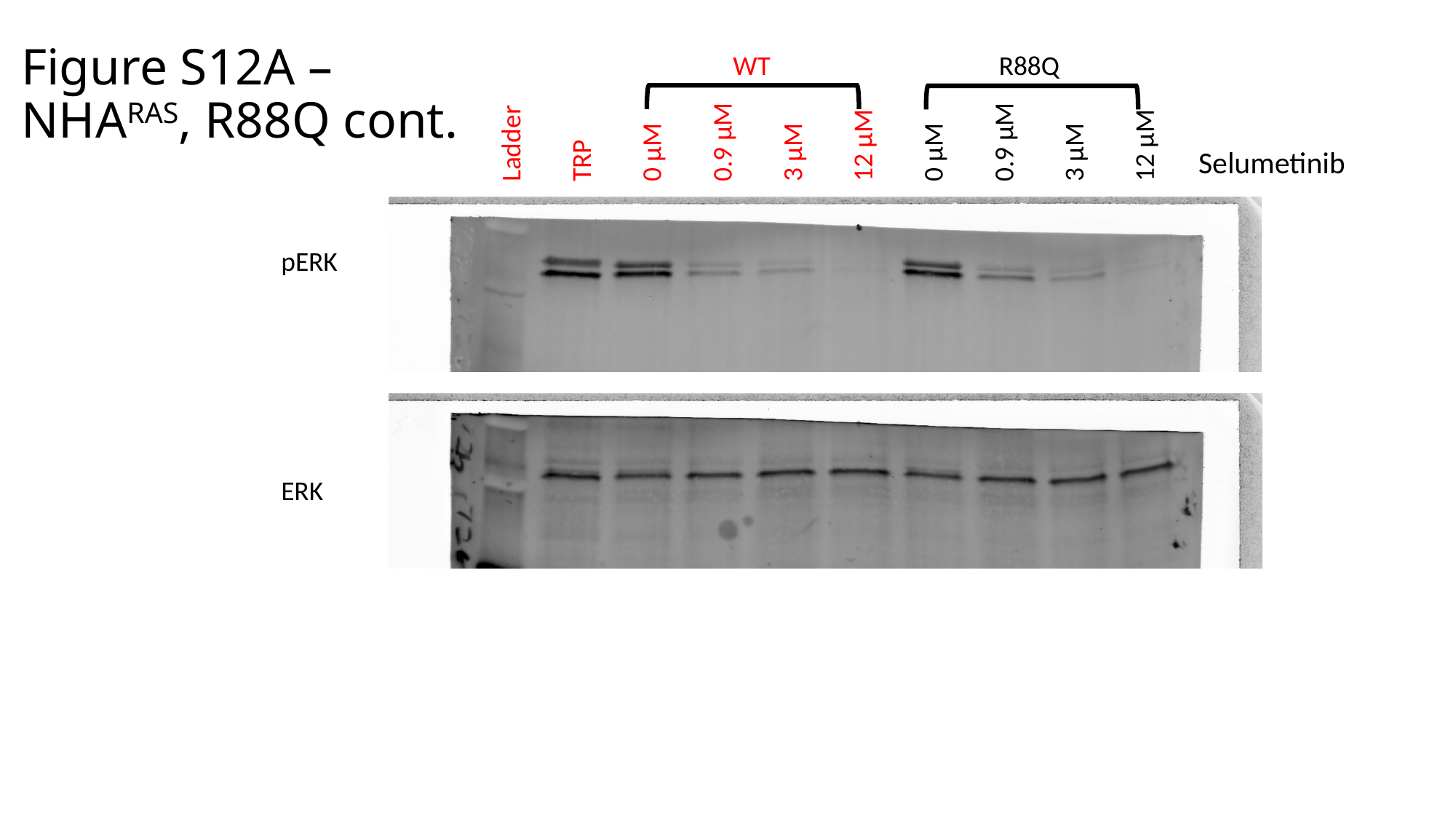

# Figure S12A – NHARAS, R88Q cont.
WT R88Q
Ladder
TRP
0 µM
0.9 µM
3 µM
12 µM
0 µM
0.9 µM
3 µM
12 µM
Selumetinib
pERK
ERK

## Slide 69
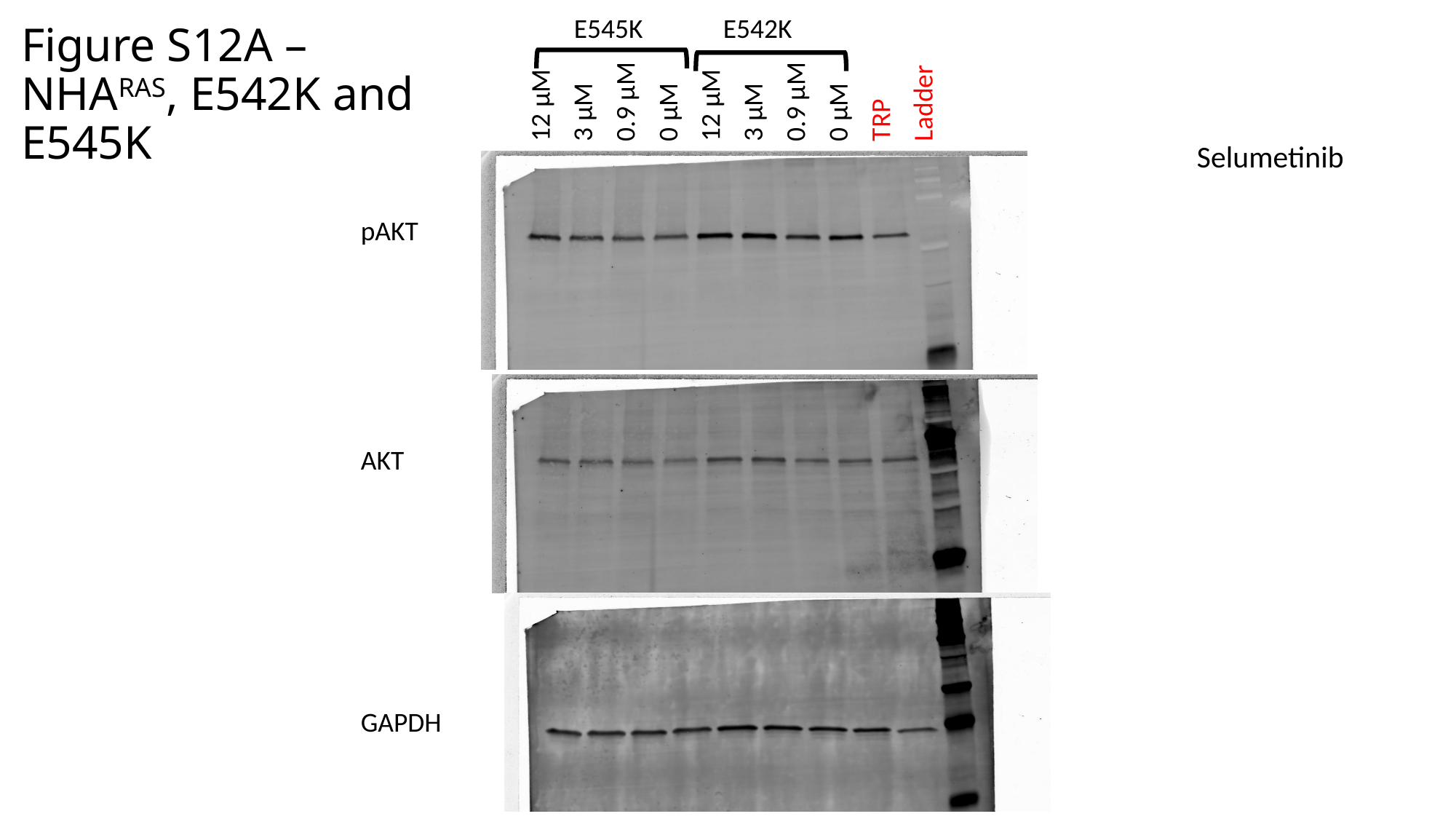

E545K E542K
12 µM
3 µM
0.9 µM
0 µM
12 µM
3 µM
0.9 µM
0 µM
TRP
Ladder
# Figure S12A – NHARAS, E542K and E545K
Selumetinib
pAKT
AKT
GAPDH

## Slide 70
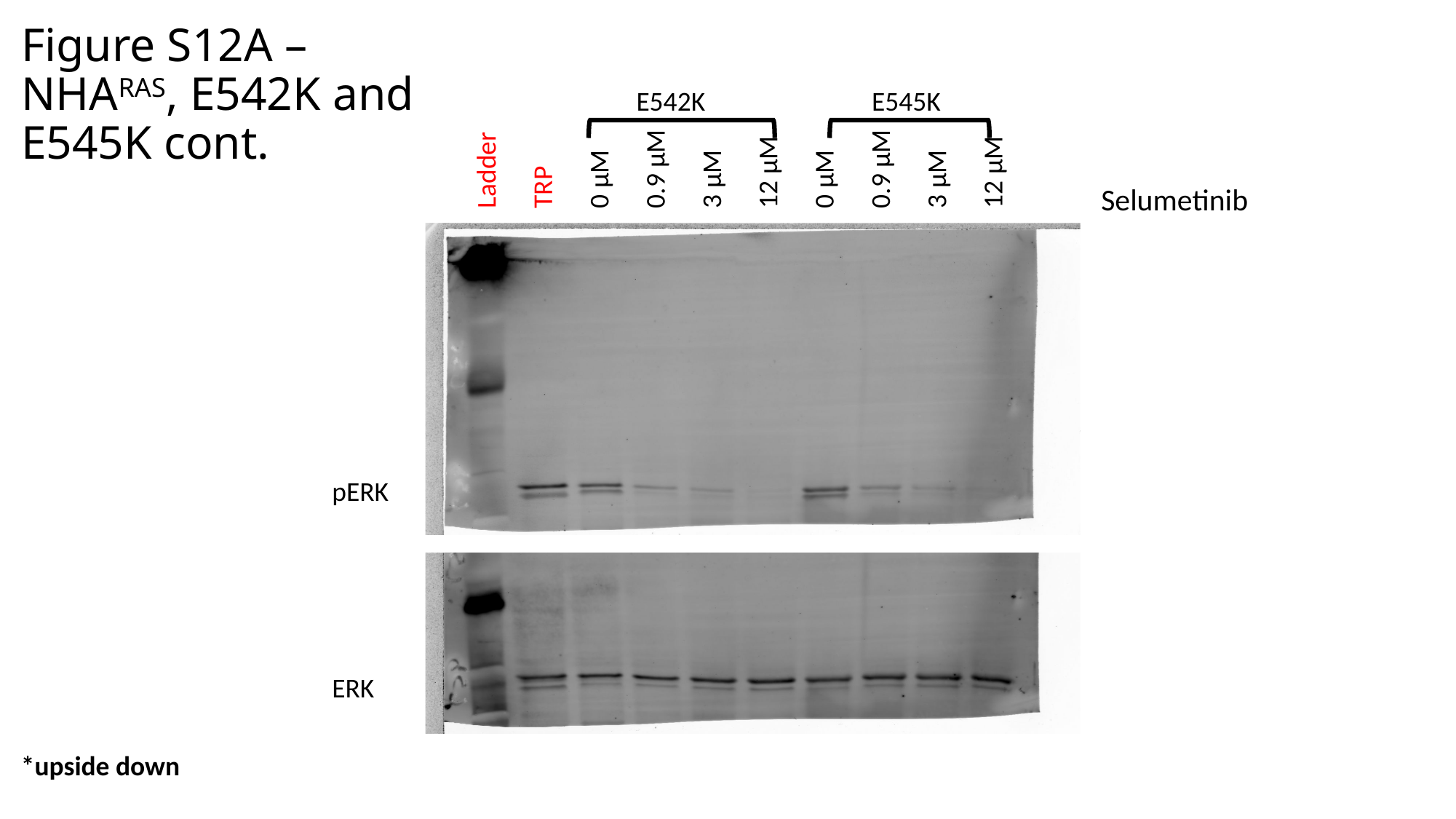

# Figure S12A – NHARAS, E542K and E545K cont.
Ladder
TRP
0 µM
0.9 µM
3 µM
12 µM
0 µM
0.9 µM
3 µM
12 µM
E542K E545K
Selumetinib
pERK
ERK
*upside down

## Slide 71
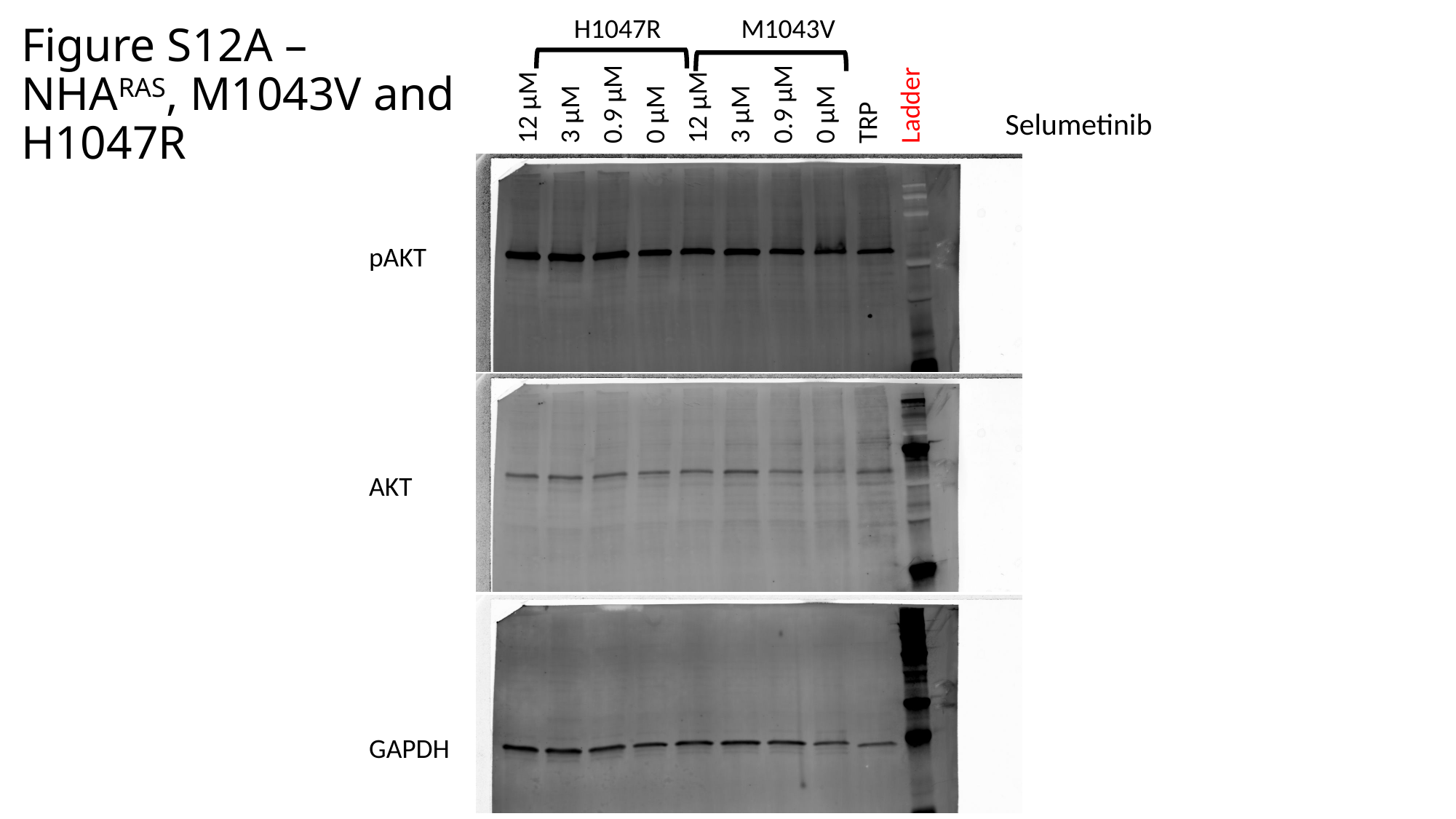

H1047R M1043V
12 µM
3 µM
0.9 µM
0 µM
12 µM
3 µM
0.9 µM
0 µM
TRP
Ladder
# Figure S12A – NHARAS, M1043V and H1047R
Selumetinib
pAKT
AKT
GAPDH

## Slide 72
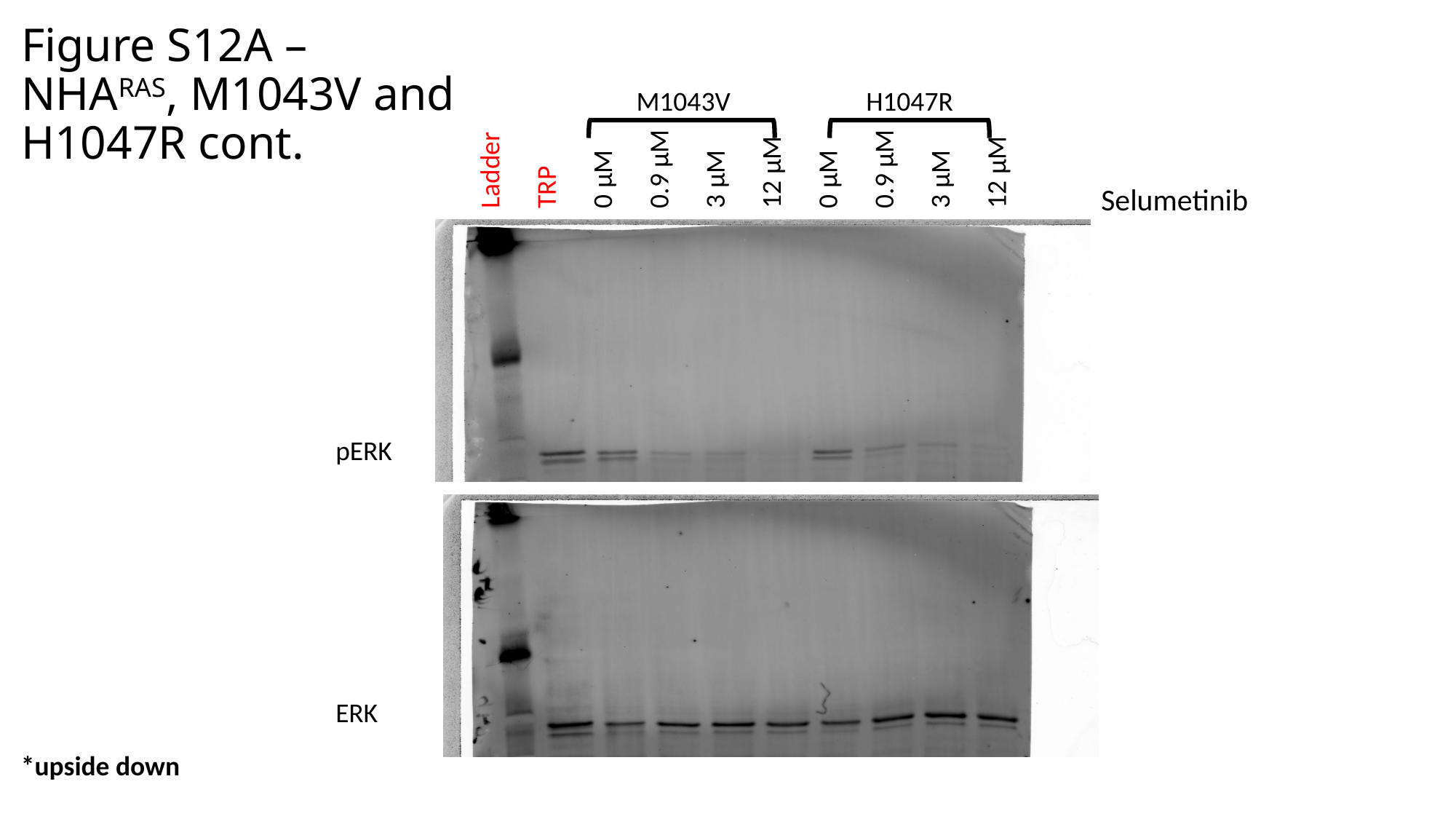

# Figure S12A – NHARAS, M1043V and H1047R cont.
Ladder
TRP
0 µM
0.9 µM
3 µM
12 µM
0 µM
0.9 µM
3 µM
12 µM
M1043V H1047R
Selumetinib
pERK
ERK
*upside down
